# The crystal structures and Hirshfeld surface analyses of four 3,5-diacetyl-2-methyl-2,3-di­hydro-1,3,4-thia­diazol-2-yl derivatives

**DOI:** 10.1107/S2056989019011915

**Published:** 2019-09-10

**Authors:** M. NizamMohideen, S. Syed Abuthahir, V. Viswanathan, D. Velmurugan, M. Karthik Ananth

**Affiliations:** a PG & Research Department of Physics, The New College (Autonomous), University of Madras, Chennai 600 014, Tamil Nadu, India; b Department of Biophysics, All India Institute of Medical Science, New Delhi 110 029, India; c CAS in Crystallography and Biophysics, University of Madras, Chennai 600 025, India; d Department of Food Quality & Safety, Institute for Postharvest and Food Sciences, Volcani Center, ARO, Rishon LeZion 7528809, Israel

**Keywords:** crystal structure, 1,3,4-thia­diazo­les, nitro­gen-containing heterocyclic compounds, acetamide, benzoate, isobutyrate, propionoate, cinnamate, hydrogen bonding, Hirshfeld surface analysis, two-dimensional fingerprint plots

## Abstract

The title compounds, 4-(5-acetamido-3-acetyl-2-methyl-2,3-di­hydro-1,3,4-thia­diazol-2-yl)phenyl benzoate, C_20_H_19_N_3_O_4_S (**I**), 4-(5-acetamido-3-acetyl-2-methyl-2,3-di­hydro-1,3,4-thia­diazol-2-yl)phenyl isobutyrate 0.25-hydrate, C_17_H_21_N_3_O_4_S·0.25H_2_O (**II**), 4-(5-acetamido-3-acetyl-2-methyl-2,3-di­hydro-1,3,4-thia­diazol-2-yl)phenyl propionate, C_16_H_19_N_3_O_4_S (**III**) and 4-(5-acetamido-3-acetyl-2-methyl-2,3-di­hydro-1,3,4-thia­diazol-2-yl)phenyl cinnamate chloro­form hemisolvate, C_22_H_21_N_3_O_4_S·0.5CHCl_3_ (**IV**), all crystallize with two independent mol­ecules (*A* and *B*) in the asymmetric unit in the triclinic *P*  space group. Compound **II** crystallizes as a quaterhydrate, while compound **IV** crystallizes as a chloro­form hemisolvate. In compounds **I**, **II**, **III** (mol­ecules *A* and *B*) and **IV** (mol­ecule *A*) the five-membered thia­diazole ring adopts an envelope conformation, with the tetra­substituted C atom as the flap. In mol­ecule *B* of **IV** this ring is flat (r.m.s. deviation 0.044 Å). The central benzene ring is in general almost normal to the mean plane of the thia­diazole ring in each mol­ecule, with dihedral angles ranging from 75.8 (1) to 85.5 (2)°. In the crystals of all four compounds, the *A* and *B* mol­ecules are linked *via* strong N—H⋯O hydrogen bonds and generate centrosymmetric four-membered *R* ^4^_4_(28) ring motifs. There are C—H⋯O hydrogen bonds present in the crystals of all four compounds, and in **I** and **II** there are also C—H⋯π inter­actions present. The inter­molecular contacts in the crystals of all four compounds were analysed using Hirshfeld surface analysis and two-dimensional fingerprint plots.

## Abstract 2

The crystal structures of four 3,5-diacetyl-2-methyl-2,3-di­hydro-1,3,4-thia­diazol-2-yl derivatives, *viz*. 4-phenyl benzoate, 4-phenyl isobutyrate, 4-phenyl propionate and 4-phenyl cinnamate, are described and the inter­molecular contacts in the crystals are analysed using Hirshfeld surface analysis and two-dimensional fingerprint plots.

## Chemical context   

Nitro­gen-containing heterocyclic compounds are one of the most important classes of biologically active compounds, exhibiting anti­microbial, anti­tumour and anti–inflammatory (Sethuram *et al.*, 2013 ▸; Huq *et al.*, 2010 ▸, Rajkumar *et al.*, 2014 ▸, 2015 ▸; Thirunavukkarsu *et al.*, 2017 ▸; Babu *et al.*, 2014*a* ▸,*b* ▸) activities. Suitably substituted 1,3,4-thia­diazo­les that incorporate the toxiphoric —N=C—S– linkage have attracted great attention owing to their broad spectrum of biological activities, including anti-inflammatory (Udupi *et al.*, 2000 ▸), herbicidal anti­microbial, bactericidal (Tehranchian *et al.*, 2005 ▸), anti­viral and anti-HIV-1 (Invidiata *et al.*, 1996 ▸) properties. Their action depends on the type and location of the polar substituents on the heterocyclic ring. In general, the pharmacological effect of potential drugs depends on the stereochemistry and ring conformations. The amide linkage [–NHC(O)–] is known to be strong enough to form and maintain protein architectures and has been utilized to create various mol­ecular devices for a range of purposes in organic chemistry. Depending on the types of substitution at the α, β and keto C atoms, and the conformational flexibility of the substituent groups, a variety of ss-acetamido ketones offering the possibility of inter­molecular inter­actions can be obtained. Recognizing the importance of such compounds in drug discovery and as part of our ongoing investigation of acetamide derivatives, the promising biological potency of 1,3,4-thia­diazo­les and variously substituted 1,3,4-thia­diazole frameworks, the title compounds have been prepared and their crystal structures are reported on herein.
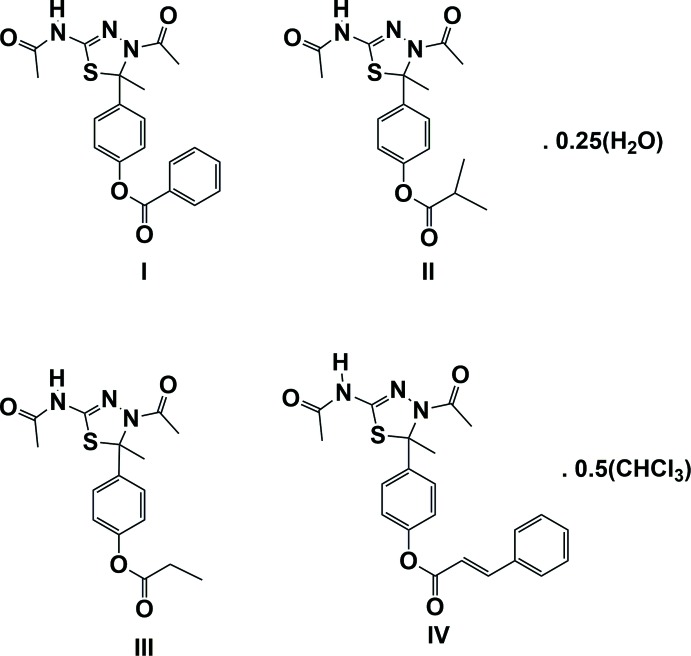


## Structural commentary   

**Figure 1:**
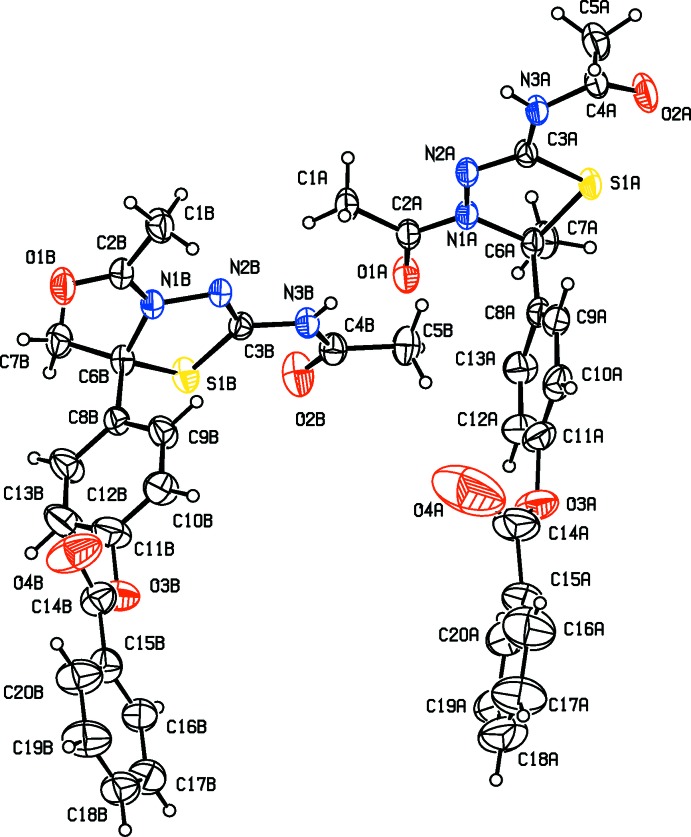
View of the mol­ecular structure of compound **I**, with atom labelling. Displacement ellipsoids are drawn at the 30% probability level. The minor disordered components have been omitted.

The mol­ecular structures and conformations of the two crystallographically independent mol­ecules (*A* and *B*), of compounds **I**, **II**, **III** and **IV** are illustrated in Figs. 1 ▸, 2 ▸, 3 ▸ and 4 ▸, respectively. In all four compounds, the bond lengths and angles in the two independent mol­ecules agree with each other. The normal probability plot analyses (Inter­national Tables for X-ray Crystallography, 1974, Vol. IV, pp. 293–309) for both bond lengths and bond angles show that the differences between the two independent mol­ecules are of a statistical nature. The geometric parameters (bond lengths and bond angles) are very similar to those observed in previously reported structures (Aridoss *et al.*, 2008 ▸).

The dihedral angle between mean plane of the thia­diazole ring [(S1/N1/N2/C3/C6) in **I** and **II**, (S1/N2/N3/C3/C6) in **III** and (S1/N1/N3/C3/C6) in **IV**] and the acetamide side chain (N3/C4/O2/C5) are 17.2 (2) and 17.3 (2)°, for compound **I** (mol­ecules *A* and *B*, respectively). In compounds **II**, **III** and **IV** the corresponding dihedral angles are 11.2 (2) and 19.6 (2)°, 61.4 (1) and 13.4 (1)° and 15.9 (1) and 6.1 (1)°, respectively. The dihedral angle between the mean plane of the thia­diazole ring and the phenyl ring (C8–C13) are respectively, 88.5 (2) and 82.8 (2)°, for mol­ecules *A* and *B* of compound **I**, and 87.8 (2) and 77.0 (1)°, respectively, for compound **II**. The corresponding dihedral angles for mol­ecules *A* and *B* are 77.2 (1) and 75.8 (1) ° in **III**, and 79.9 (1) and 87.0 (1)° in **IV**. The dihedral angle between phenyl ring (C8–C13) and the acetamide side chain (N3/C4/O2/C5) are 86.9 (2) and 80.2 (2)°, for compound **I** (mol­ecules *A* and *B*, respectively). In compound **II**, for mol­ecules *A* and *B*, the corresponding angles are 84.2 (2) and 81.6 (2)°, respectively.

In mol­ecules *A* and *B* of compounds **I**, **II**, **III** and mol­ecule *B* of compound **IV**, the thia­diazole rings (S1/C3/N2/N3/C6) adopt envelope conformations, with atom C6 deviating from the mean plane of the remaining four atoms: by 0.132 (3) and 0.110 (3) Å, for atoms C6*A* and C6*B*, respectively, for **I**, 0.132 (2) and 0.136 (2) Å for **II**, 0.395 (3) and 0.350 (3) Å for **III** and 0.321 (3) Å for mol­ecule *B* of **IV**. In mol­ecule *B* of compound **IV**, this ring is planar (r.m.s. deviation 0.044 Å).

In three of the compounds there is a certain disorder; in compound **I** the phenyl benzoate group is disordered, in compound **II** the methyl propano­ate group is disordered, and in compound **I**II the O atom of the ester group is disordered. The geometries were regularized using soft restraints; see §7, *Refinement*.

## Supra­molecular features   

**Figure 5:**
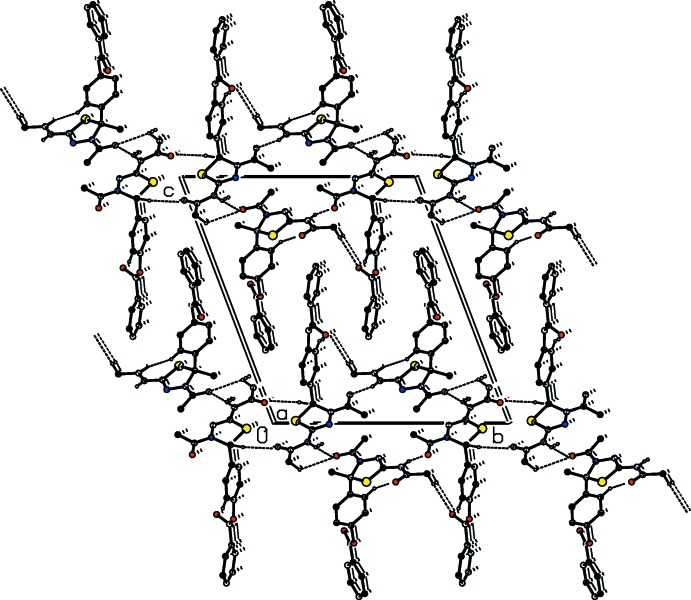
The crystal packing of compound **I**, viewed along the *a* axis. The hydrogen bonds (see Table 1 ▸) are shown as dashed lines. For clarity, the H atoms not involved in the hydrogen bonding have been omitted.

In all compounds, the crystal packing is stabilized by strong N—H⋯O inter­molecular hydrogen bonds (see Tables 1 ▸, 2 ▸, 3 ▸ and 4 ▸, and Figs. 5 ▸, 6 ▸, 7 ▸ and 8 ▸).

In the crystals of all four compounds, the *A* and *B* mol­ecules are linked *via* strong N—H⋯O hydrogen bonds and generate centrosymmetric four-membered 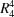
(28) ring motifs. There are C—H⋯O hydrogen bonds present in the crystals of all four compounds. For **I** they link the rings to form layers parallel to the *ab* plane, while for **II** they link the rings, that stack up the *a* axis, to form columns. For **III**, neighbouring rings are linked by C—H⋯O hydrogen bonds to form ribbons propagating along the *b*-axis direction. Finally, for **IV**, the rings that stack up the *b*-axis are linked by C—H⋯O hydrogen bonds to form columns, which are linked by a further C—H⋯O hydrogen bond to form a supra­molecular three-dimensional structure.

In the crystals of **I** and **II**, there are also C—H⋯π inter­actions present. In the former they link the layers, while in the latter they link the columns, to form supra­molecular three-dimensional structures.

## Hirshfeld surface analysis   

A recent article by Tiekink and collaborators (Tan *et al.*, 2019 ▸) reviews and describes the uses and utility of Hirshfeld surface analysis (Spackman & Jayatilaka, 2009 ▸), and the associated two-dimensional fingerprint plots (McKinnon *et al.*, 2007 ▸), to analyse inter­molecular contacts in crystals. The various calculations were performed with *CrystalExplorer17* (Turner *et al.*, 2017 ▸).

**Figure 9:**
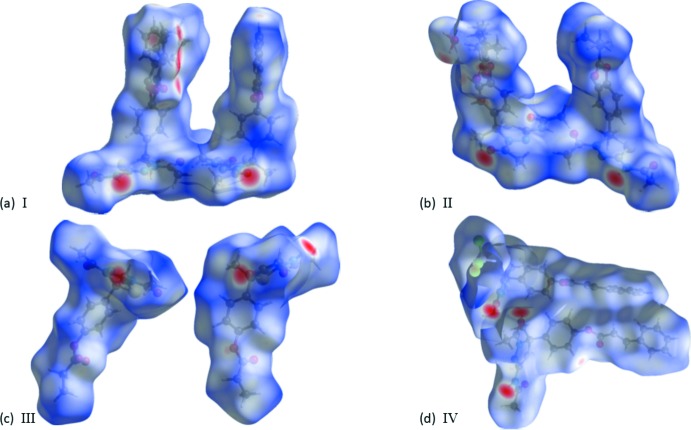
The Hirshfeld surfaces of compounds (*a*) **I**, (*b*) **II**, (*c*) **III** and (*d*) **IV** mapped over *d*_norm_

**Figure 10:**
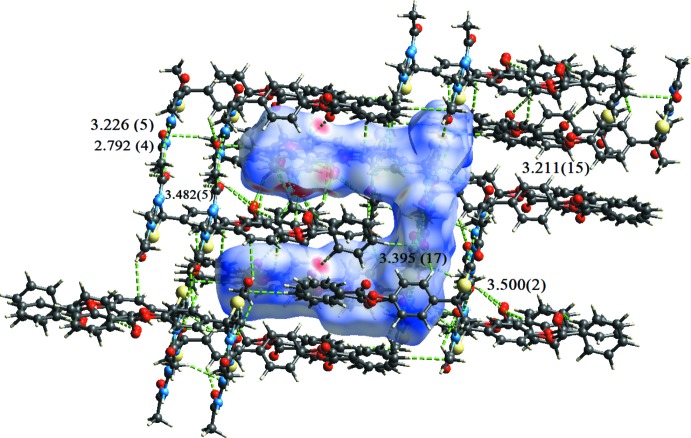
A view of the Hirshfeld surface mapped over *d*_norm_ of compound **I**, showing the various inter­molecular contacts in the crystal.

**Figure 11:**
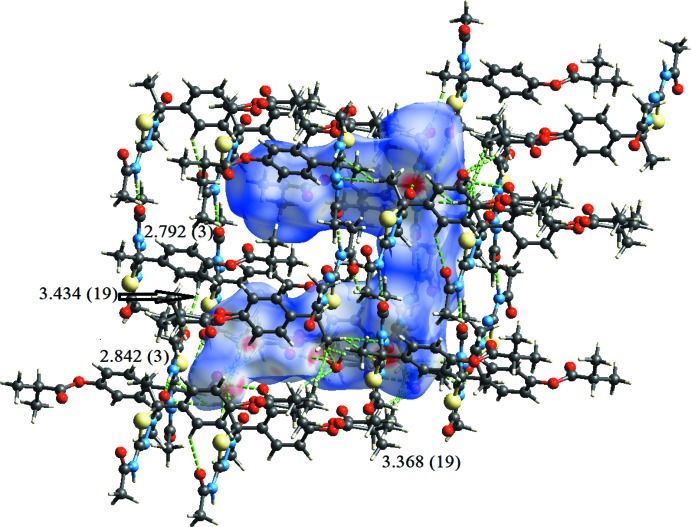
A view of the Hirshfeld surface mapped over *d*_norm_ of compound **II**, showing the various inter­molecular contacts in the crystal.

**Figure 12:**
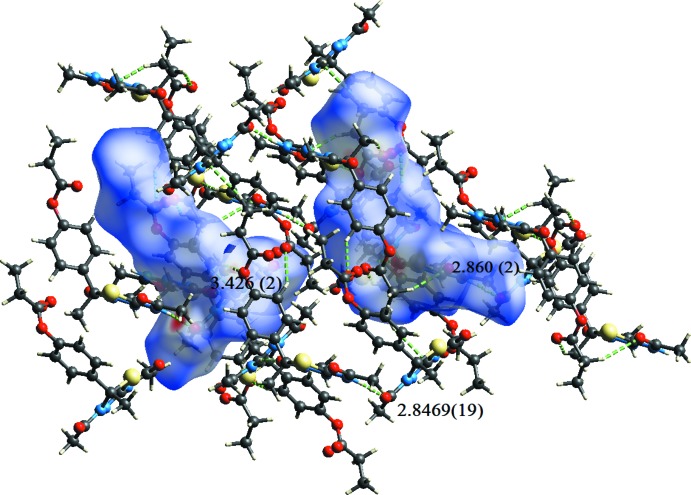
A view of the Hirshfeld surface mapped over *d*_norm_ of compound **III**, showing the various inter­molecular contacts in the crystal.

**Figure 13:**
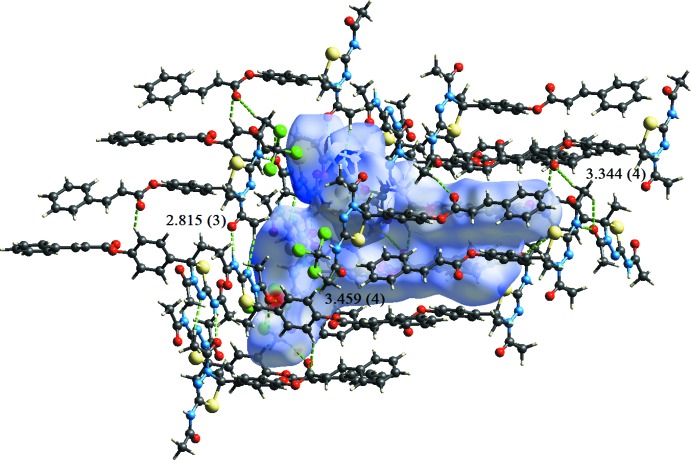
A view of the Hirshfeld surface mapped over *d*_norm_ of compound **IV**, showing the various inter­molecular contacts in the crystal.

The Hirshfeld surfaces of compounds **I**–**IV** mapped over *d*_norm_ are given in Fig. 9 ▸, and the inter­molecular contacts are illustrated in Fig. 10 ▸ for **I**, Fig. 11 ▸ for **II**, Fig. 12 ▸ for **III** and Fig. 13 ▸ for **IV**. They are colour-mapped with the normalized contact distance, *d*_norm_, ranging from red (distances shorter than the sum of the van der Waals radii) through white to blue (distances longer than the sum of the van der Waals radii). The *d*_norm_ surface was mapped over a fixed colour scale of −0.763 (red) to 1.539 (blue) for compound **I**, −0.593 (red) to 1.357 (blue) for compound **II**, −0.593 (red) to 1.607 (blue) for compound **III** and −0.617 (red) to 2.422 (blue) for compound **IV**, where the red spots indicate the inter­molecular contacts involved in the hydrogen bonding.

**Figure 14:**
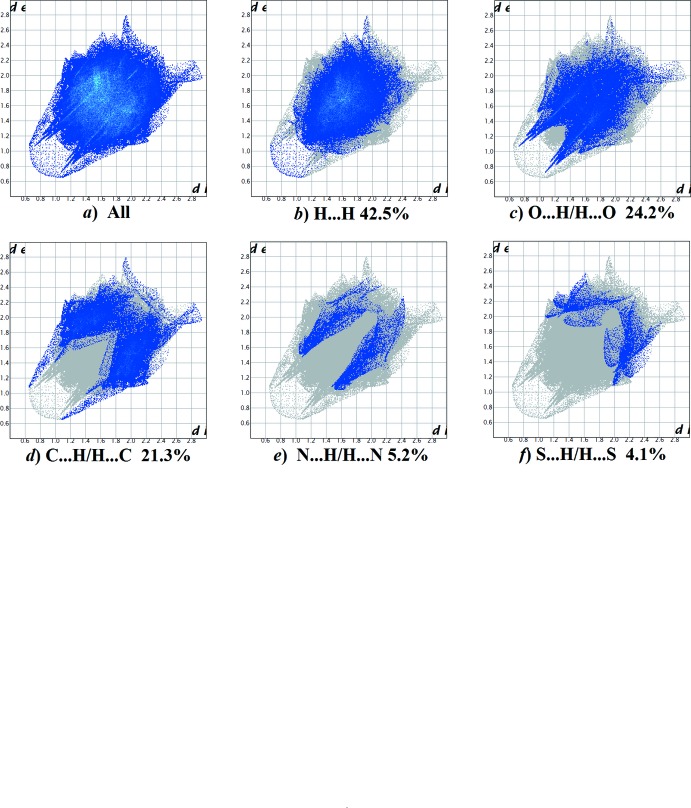
The full two-dimensional fingerprint plot for compound **I**, and fingerprint plots delineated into (*b*) H⋯H, (*c*) O⋯H/H⋯O, (*d*) C⋯H/H⋯C (*e*) N⋯H/H⋯N and (*f*) S⋯H/H⋯S contacts.

**Figure 15:**
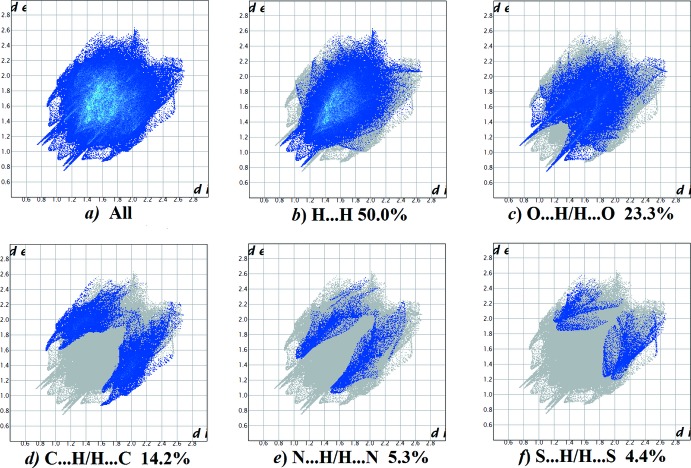
The full two-dimensional fingerprint plot for compound **II**, and fingerprint plots delineated into (*b*) H⋯H, (*c*) O⋯H/H⋯O, (*d*) C⋯H/H⋯C (*e*) N⋯H/H⋯N and (*f*) S⋯H/H⋯S contacts.

**Figure 16:**
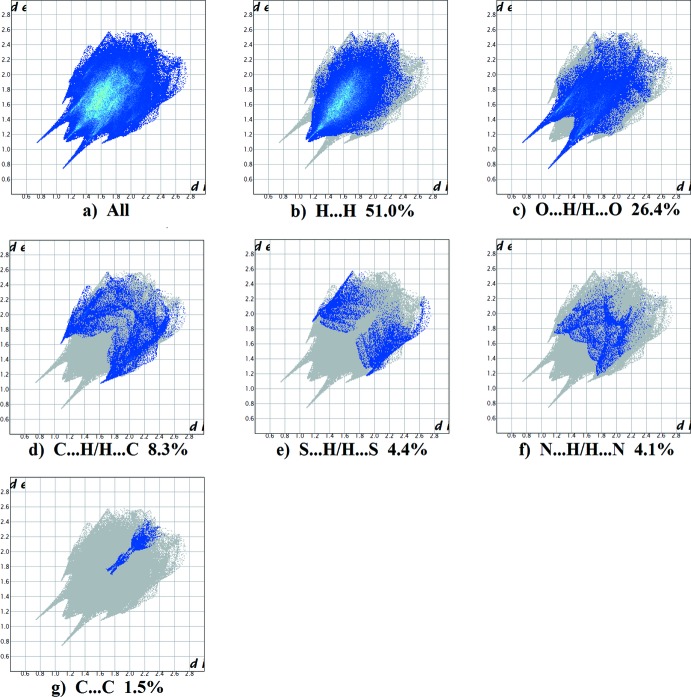
The full two-dimensional fingerprint plot for compound **III**, and fingerprint plots delineated into (*b*) H⋯H, (*c*) O⋯H/H⋯O, (*d*) C⋯H/H⋯C (*e*) S⋯H/H⋯S, (*f*) N⋯H/H⋯N and (*g*) C⋯C contacts.

**Figure 17:**
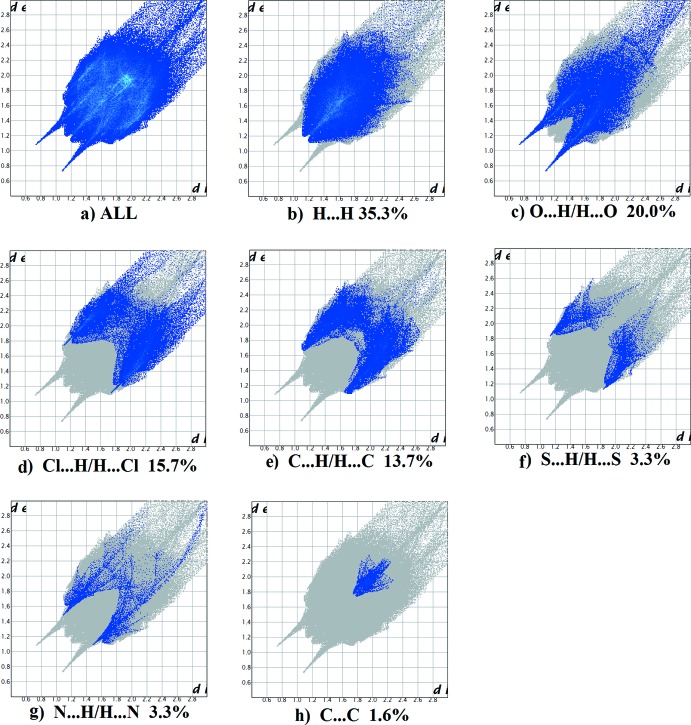
The full two-dimensional fingerprint plot for compound **IV**, and fingerprint plots delineated into (*b*) H⋯H, (*c*) O⋯H/H⋯O, (*d*) Cl⋯H/H⋯Cl, (*e*) C⋯H/H⋯C, (*f*) S⋯H/H⋯S, (*g*) N⋯·H/H⋯N and (*h*) C⋯C contacts.

The fingerprint plots are given in Figs. 14 ▸, 15 ▸, 16 ▸ and 17 ▸, revealing similar trends for the principal inter­molecular contacts. For compound **I**, they reveal that the principal inter­molecular contacts are H⋯H at 42.5% (Fig. 14 ▸ *b*), O⋯H/H⋯O at 24.2% (Fig. 14 ▸ *c*), C⋯H/H⋯C contacts at 21.3% (Fig. 14 ▸ *d*) and N⋯H/H⋯N at 5.2% (Fig. 14 ▸ *e*), followed by the S⋯H/H⋯S at 4.1% (Fig. 14 ▸ *f*). For compound **II**, the principal inter­molecular contacts are H⋯H at 50.0% (Fig. 15 ▸ *b*), O⋯H/H⋯O at 23.3% (Fig. 15 ▸ *c*), C⋯H/H⋯C contacts at 14.2% (Fig. 15 ▸ *d*) and N⋯H/H⋯N at 5.3% (Fig. 15 ▸ *e*) followed by the S⋯H/H⋯S at 4.4% (Fig. 15 ▸ *f*). For compound **III**, the principal inter­molecular contacts are H⋯H at 51.0% (Fig. 16 ▸ *b*), O⋯H/H⋯O at 26.4% (Fig. 16 ▸ *c*), C⋯H/H⋯C contacts at 8.3% (Fig. 16 ▸ *d*) and S⋯H/H⋯S at 4.4% (Fig. 15 ▸ *e*) followed by the N⋯H/H⋯N at 4.1% (Fig. 15 ▸ *f*) and C⋯ C contacts at 1.5%. For compound **IV**, the principal inter­molecular contacts are H⋯H at 35.3% (Fig. 17 ▸ *b*), O⋯H/H⋯O at 20.0% (Fig. 17 ▸ *c*), Cl⋯H/H⋯Cl at 15.7% (Fig. 17 ▸ *d*), C⋯H/H⋯C at 13.7% (Fig. 17 ▸ *e*), S⋯H/H⋯S at 3.3% (Fig. 17 ▸ *f*), N⋯H/H⋯N at 3.3% (Fig. 17 ▸ *c*) followed by the C⋯C contacts at 1.6% (Fig. 17 ▸ *h*). In all compounds, the H⋯H inter­molecular contacts predominate, followed by the O⋯H/H⋯O contacts.

## Database survey   

A search of the Cambridge Structural Database (Version 5.40, last update May 2019; Groom *et al.*, 2016 ▸) for (5-acetamido-3-acetyl-2-methyl-2,3-di­hydro-1,3,4-thia­diazol-2-yl)phenyl rev­ealed the presence of three relevant compounds, *viz. N*-(4-acetyl-5-(4-fluoro­phen­yl)-4,5-di­hydro-1,3,4-thia­diazol-2-yl) acetamide (CSD refcode IDOFOY; Kavitha *et al.*, 2013 ▸), *N*-(4-acetyl-5-(3-meth­oxy­phen­yl)-4,5-di­hydro-1,3,4-thia­diazol-2-yl) acetamide (IGAREO; Aridoss *et al.*, 2008 ▸), that crystallized in space group *P*2_1_ with two independent mol­ecules in the asymmetric unit, and 2-acetyl­amino-4-acetyl-5-phenyl-Δ^2^-1,3,4-thia­diazo­line (YOLKAL; Usova *et al.*, 1994 ▸). Here, the mean plane of the thia­diazole ring is almost normal to the 5-phenyl ring with dihedral angles of *ca* 86.82, 88.50 (68.46) and 84.06°, respectively. This situation is very similar to that in the title compounds where this dihedral angle varies from 75.8 (1) to 85.5 (2)°.

## Synthesis and crystallization   

**Synthesis of 4-(3,5-diacetyl-2-methyl-2,3-di­hydro-1,3,4-thia­diazol-2-yl)phenyl benzoate (I)** To a clean and dry 250 ml two-neck round-bottom flask fitted with condenser and addition funnel containing 4-hy­droxy aceto­phenone (0.5 mol) was added chloro­form (200 ml) under continuous stirring and the reaction mixture was cooled to 288–293 K. Benzoyl chloride (0.5 mol) was added dropwise and stirring continued for a further 15 min and then potassium carbonate (0.5 mol) was slowly added. The reaction continued for another 4 h, monitored using TLC. The reaction mass was transferred into a 1 l beaker and washed twice with water (2 × 250 ml). The chloro­form layer was separated and washed with a 10% NaOH solution (2 × 250 ml) and dried with anhydrous sodium sulfate followed by concentration under reduced pressure using rotary vacuum before being cooled and hexane added. Thio­semicarbazide (0.1 mol) dissolved in 20 ml of 1 *N* hydro­chloric acid was added slowly under stirring to 4-acetyl­phenyl benzoate (0.1 mol) dissolved in 50 ml of ethanol. After the addition of thio­semicarbazide, 4-[(1-(2-carbamo­thio­ylhydrazinyl­idene)eth­yl]phenyl benzoate (in solid form) was formed within 4 min. The precipitate was filtered and washed with water, followed by hexane. 4-[(1-(2-Carbamo­thio­ylhydrazinyl­idene)eth­yl]phenyl benzoate (0.5 mol) was dissolved in 10 ml of acetic anhydride and the mixture heated at 383 K for 3 h with magnetic stirring. The reaction was monitored using TLC, and once complete the reaction mass was quenched in crushed ice with stirring. The solid product obtained was filtered, washed with cold water followed by hexane and then air-dried. Recrystallization using chloro­form yielded colourless block-like crystals of compound **I**.

**Synthesis of 4-(3,5-diacetyl-2-methyl-2,3-di­hydro-1,3,4-thia­diazol-2-yl)phenyl isobutyrate (II)** To a clean and dry 250 ml two-neck round-bottom flask fitted with condenser and addition funnel containing 4-hy­droxy aceto­phenone (0.5 mol) was added chloro­form (200 ml) under continuous stirring and the reaction mixture was cooled to 288–293 K. Isobutyryl chloride (0.5 mol) was added dropwise and stirring continued for a further 15 min and then potassium carbonate (0.5 mol) was slowly added. The reaction continued for another 4 h, monitored using TLC. The reaction mass was then transferred into a 1 l beaker and washed twice with water (2 × 250 ml). The chloro­form layer was separated and washed with a 10% NaOH solution (2 × 250 ml) and dried with anhydrous sodium sulfate then concentrated under reduced pressure using a rotary vacuum, cooled and hexane was added. Thio­semicarbazide (0.91 g, 0.01 mol) was added to a 50 ml ethano­lic solution of 4-acetyl­phenyl isobutyrate (0.01 mol) under continuous stirring. The resulting mixture refluxed at 333 K and the purity of the products as well as the composition of the reaction was monitored by TLC using ethyl acetate: hexane (3:7). The reaction mixture was cooled to room temperature and the separated product was filtered. 4-[(1-(2-Carbamo­thio­yl­hydra­zinyl­idene)eth­yl]phenyl 2-methyl­prop­an­o­ate (0.5 mol) was dissolved in 10 ml of acetic anhydride and the mixture was heated at 383 K for 3 h under magnetic stirring. The reaction was monitored using TLC, and once complete the reaction mass was quenched in crushed ice cubes with stirring. The solid product obtained was filtered, washed with cold water followed by hexane and then air-dried. Recrystallization using chloro­form yielded colourless block-like crystals of compound **II**.

**Synthesis of 4-(3,5-diacetyl-2-methyl-2,3-di­hydro-1,3,4-thia­diazol-2-yl)phenyl propionate (III)** To a clean and dry 250 ml two-neck round-bottom flask fitted with condenser and addition funnel containing 4-hy­droxy aceto­phenone (0.5 mol) was added chloro­form (200 ml) under continuous stirring and the reaction mixture was cooled to 288–293 K. Propanoyl chloride (0.5 mol) was then added dropwise. Stirring continued for another 15 min and then potassium carbonate (0.5 mol) was slowly added. The reaction was continued for another 4 h and monitored using TLC. The reaction mass was transferred into a 1 l beaker and washed twice with water (2 × 250 ml). The chloro­form layer was separated and washed with a 10% NaOH solution (2 × 250 ml) and dried with anhydrous sodium sulfate followed by concentration under reduced pressure using a rotary vacuum, cooled and hexane was added to it. Thio­semicarbazide (0.91g, 0.01 mol) was added to 50 ml of an ethano­lic solution of 4-acetyl­phenyl propionate (0.01 mol) under continuous stirring. The resulting mixture was refluxed at 333 K and the purity of the products as well as composition of the reaction was monitored by TLC using ethyl acetate:hexane (3:7). The reaction mixture was cooled to room temperature and the separated product was filtered. 4-[(1-(2 Carbamo­thioyl hydrazinyl­idene)eth­yl]phenyl propano­ate (0.5 mol) was dissolved in 10 ml of acetic anhydride and the mixture was heated at 383 K for 3 h under magnetic stirring. The reaction was monitored using TLC, and once complete the mass was quenched in crushed ice under stirring. The solid product obtained was filtered, washed with cold water followed by hexane and then air-dried. Recrystallization using chloro­form yielded colourless block-like crystals of compound **III**.

**Synthesis of 4-(3,5-diacetyl-2-methyl-2,3-di­hydro-1,3,4-thia­diazol-2-yl)phenyl cinnamate (IV)** To a clean and dry 250 ml two-neck round-bottom flask fitted with condenser and addition funnel containing 4-hy­droxy aceto­phenone (0.5 mol) was added chloro­form (200 ml) under continuous stirring and the reaction mixture was cooled to 288–293 K. Cinnamoyl chloride (0.5 mol) was then added dropwise. Stirring continued for another 15 min and potassium carbonate (0.5 mol) was slowly added. The reaction continued for another 4 h and was monitored using TLC. The reaction mass was transferred into a 1 l beaker and washed twice with water (2 × 250 ml). The chloro­form layer separated and was washed with a 10% NaOH solution (2 × 250 ml) and dried with anhydrous sodium sulfate followed by concentration under reduced pressure using a rotary vacuum, cooled and hexane added. Thio­semicarbazide (0.1 mol) dissolved in 20 ml of 1 *N* hydro­chloric acid was added slowly under stirring to 4-acetyl­phenyl cinnamate (0.1 mol) dissolved in 50 ml of ethanol. After the addition of thio­semicarbazide, 4-[(1-(2-carbamo­thio­ylhydrazinyl­idene)eth­yl]phenyl benzoate (in solid form) was formed within 4 min. The precipitate was filtered off and washed with water, followed by hexane. 4-[(1-(2-Carbamo­thio­ylhydrazinyl­idene)eth­yl]phenyl-3-phenyl­prop-2-enoate (0.5 mol) was dissolved in 10 ml of acetic anhydride and the mixture was heated at 383 K for 3 h under magnetic stirring. The reaction was monitored using TLC, and once complete the reaction mass was quenched in crushed ice under stirring. The solid product obtained was filtered, washed with cold water followed by hexane and then air-dried. Recrystallization using chloro­form yielded colourless block-like crystals of compound **IV**.

## Refinement   

Crystal data, data collection and structure refinement details are summarized in Table 5 ▸. For compounds **I** and **II**, the NH H atoms were located in difference-Fourier maps and freely refined. For compounds **III** and **IV** they were included in calculated positions and refined as riding: N—H = 0.93 Å with *U*_iso_(H) = 1.2*U*_eq_(N). All C-bound H atoms were positioned geometrically and constrained to ride on their parent atoms: C—H = 0.93–0.98 Å with *U*_iso_(H) = 1.5*U*_eq_(C-meth­yl) and 1.2*U*_eq_(C) for other H atoms. In compound **I**, the phenyl benzoate group is disordered [occupancy ratios of 0.553 (5): 0.447 (5) and 0.661 (6):0.339 (6) in mol­ecules *A* and *B*, respectively]. In compound **II**, the methyl propano­ate group in mol­ecule *B* is disordered [occupancy ratio 0.723 (5):0.277 (5)]. In compound **III**, the O atom of the ester group of mol­ecule *B* is disordered [occupancy ratio of 0.68 (6):0.32 (6)]. The geometries were regularized using soft restraints.

## Supplementary Material

Crystal structure: contains datablock(s) global, I, II, III, IV. DOI: 10.1107/S2056989019011915/su5508sup1.cif

Structure factors: contains datablock(s) I. DOI: 10.1107/S2056989019011915/su5508Isup5.hkl

Structure factors: contains datablock(s) III. DOI: 10.1107/S2056989019011915/su5508IIIsup7.hkl

Structure factors: contains datablock(s) IV. DOI: 10.1107/S2056989019011915/su5508IVsup8.hkl

Supporting information file. DOI: 10.1107/S2056989019011915/su5508Isup5.cml

Supporting information file. DOI: 10.1107/S2056989019011915/su5508IIsup6.cml

Supporting information file. DOI: 10.1107/S2056989019011915/su5508IIIsup7.cml

Supporting information file. DOI: 10.1107/S2056989019011915/su5508IVsup8.cml

CCDC references: 1909902, 1911310, 1909897, 1909898

Additional supporting information:  crystallographic information; 3D view; checkCIF report

## Figures and Tables

**Figure 2:**
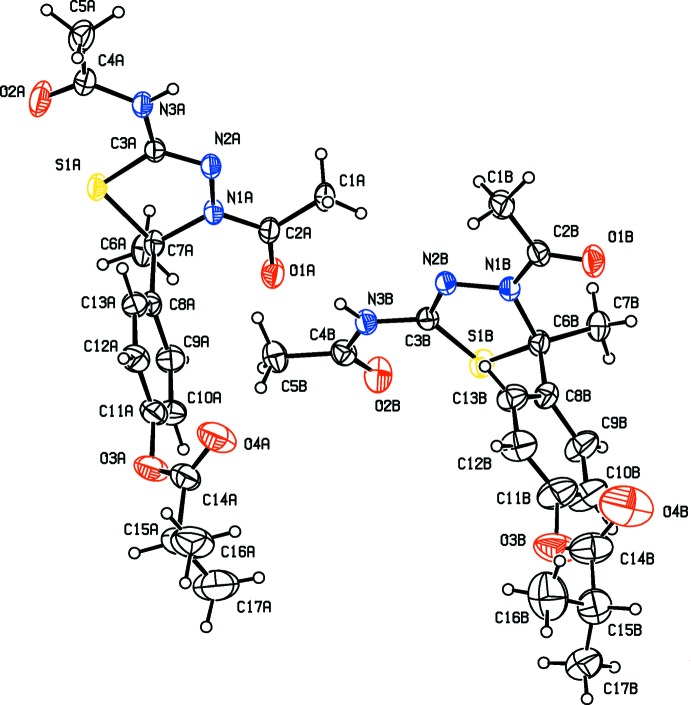
View of the mol­ecular structure of compound **II**, with atom labelling. Displacement ellipsoids are drawn at the 30% probability level. The solvent water mol­ecule and the minor disordered component have been omitted.

**Figure 3:**
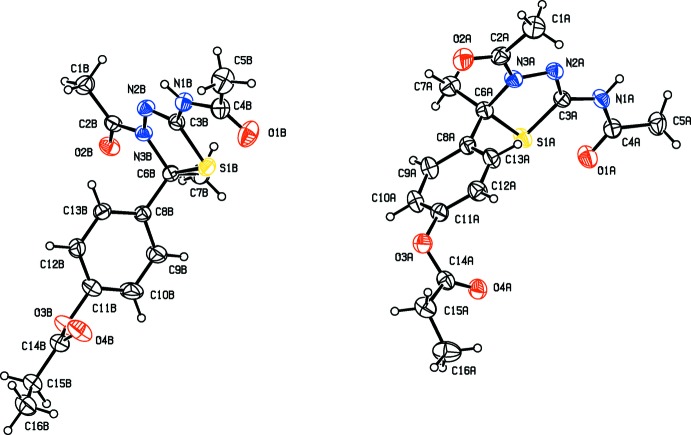
View of the mol­ecular structure of compound **III**, with atom labelling. Displacement ellipsoids are drawn at the 30% probability level. The minor disordered component has been omitted.

**Figure 4:**
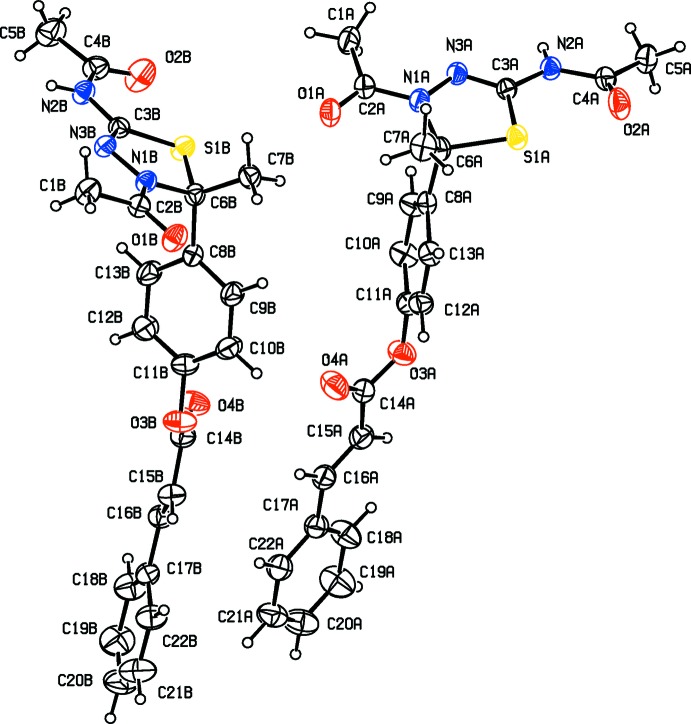
View of the mol­ecular structure of compound **IV**, with atom labelling. Displacement ellipsoids are drawn at the 30% probability level. The solvent CHCl_3_ mol­ecule has been omitted.

**Figure 6:**
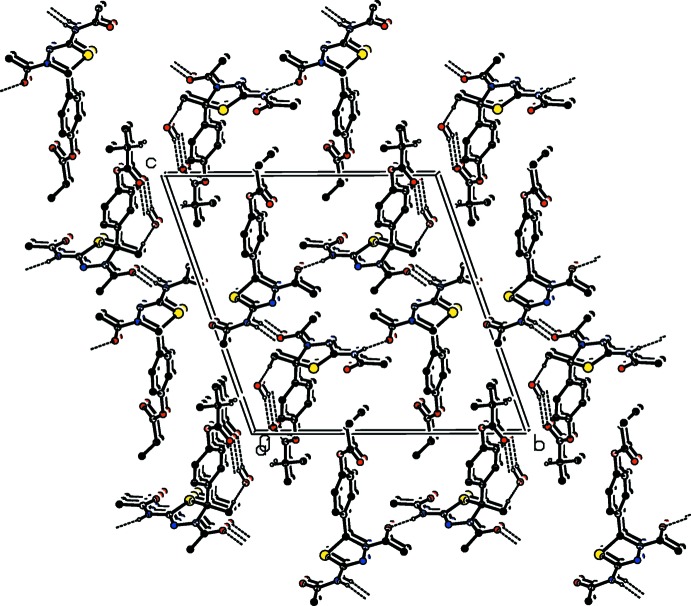
Part of the crystal structure of **II**, viewed along the *a* axis. The hydrogen bonds (see Table 2 ▸) are shown as dashed lines. For clarity, the H atoms not involved in the hydrogen bonding have been omitted.

**Figure 7:**
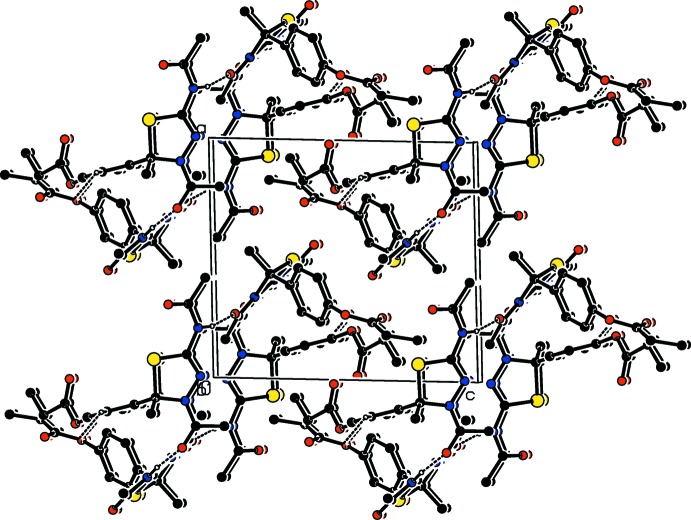
The crystal packing of compound **III**, viewed along the *b* axis. The hydrogen bonds (see Table 3 ▸) are shown as dashed lines. For clarity, the H atoms not involved in the hydrogen bonding have been omitted.

**Figure 8:**
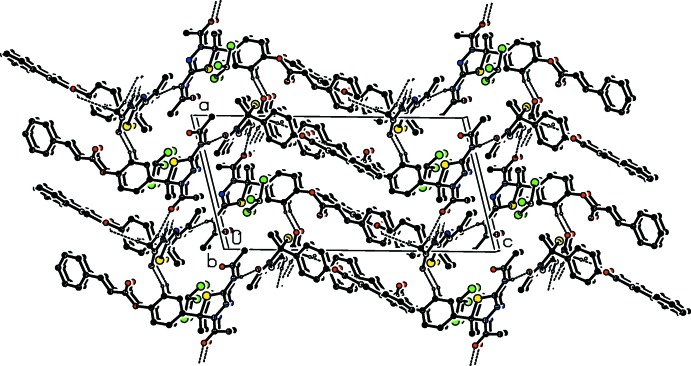
The crystal packing of compound **IV**, viewed along the *b* axis. The hydrogen bonds (see Table 4 ▸) are shown as dashed lines. For clarity, the H atoms not involved in the hydrogen bonding have been omitted.

**Table 1:** Hydrogen-bond geometry (Å, °) for **I[Chem scheme1]** *Cg*2, *Cg*3 and *Cg*6 are the centroids of the C8*A*–C13*A*, C15*A*–C20*A* and C8*B*–C13*B* rings, respectively.

*D*—H⋯*A*	*D*—H	H⋯*A*	*D*⋯*A*	*D*—H⋯*A*
N3*A*—H3*A*⋯O1*B* ^i^	0.84 (4)	1.96 (3)	2.792 (4)	175 (3)
N3*B*—H3*B*⋯O1*A*	0.84 (4)	1.99 (5)	2.801 (4)	163 (4)
C5*A*—H5*A*2⋯O1*B* ^i^	0.96	2.59	3.226 (5)	124
C7*A*—H7*A*2⋯O2*A* ^ii^	0.96	2.54	3.482 (5)	168
C9*B*—H9*B*⋯O2*B* ^iii^	0.93	2.58	3.303 (5)	135
C5*B*—H5*B*1⋯O4*A* ^iv^	0.96	2.59	3.50 (2)	158
C5*B*—H5*B*1⋯O4*C* ^iv^	0.96	2.45	3.395 (17)	166
C12*A*—H12*A*⋯O4*C* ^iv^	0.93	2.58	3.211 (15)	125
C17*B*—H17*B*⋯*Cg*2^v^	0.93	2.91	3.664 (8)	139
C17*C*—H17*C*⋯*Cg*6^v^	0.93	2.98	3.776 (10)	145
C20*C*—H20*C*⋯*Cg*3^vi^	0.93	2.64	3.521 (11)	159

Symmetry codes: (i) 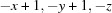; (ii) 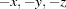; (iii) 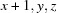; (iv) 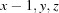; (v) 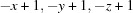; (vi) 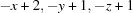.

**Table 2:** Hydrogen-bond geometry (Å, °) for **II[Chem scheme1]** *Cg*2 and *Cg*4 are the centroids of the C8*B*–C13*B* and C8*A*–C13*A* rings, respectively.

*D*—H⋯*A*	*D*—H	H⋯*A*	*D*⋯*A*	*D*—H⋯*A*
O1—H1*B*⋯O4*B* ^i^	0.88 (11)	2.32 (10)	3.111 (16)	149 (12)
N3*A*—H3*A*⋯O1*B* ^ii^	0.86	1.99	2.842 (3)	171
N3*B*—H3*B*⋯O1*A*	0.86	1.94	2.792 (3)	171
C15*B*—H15*B*⋯O1	0.98	2.46	3.368 (19)	154
C7*B*—H7*B*2⋯O1^iii^	0.96	2.49	3.434 (19)	168
C15*A*—H15*A*⋯*Cg*2^iv^	0.98	2.99	3.959 (4)	168
C17*B*—H17*B*⋯*Cg*4^iv^	0.96	2.98	3.864 (9)	153
C17′—H17*H*⋯*Cg*4^iv^	0.96	2.93	3.81 (3)	154

Symmetry codes: (i) 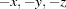; (ii) 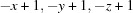; (iii) 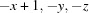; (iv) 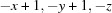.

**Table 3:** Hydrogen-bond geometry (Å, °) for **III[Chem scheme1]**

*D*—H⋯*A*	*D*—H	H⋯*A*	*D*⋯*A*	*D*—H⋯*A*
N1*A*—H1*A*⋯O2*B* ^i^	0.86	1.99	2.8469 (19)	174
N1*B*—H1*B*⋯O2*A* ^ii^	0.86	2.04	2.860 (2)	160
C9*B*—H9*B*⋯O3*A* ^iii^	0.93	2.60	3.426 (2)	148

Symmetry codes: (i) 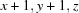; (ii) 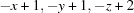; (iii) 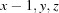.

**Table 4:** Hydrogen-bond geometry (Å, °) for **IV[Chem scheme1]**

*D*—H⋯*A*	*D*—H	H⋯*A*	*D*⋯*A*	*D*—H⋯*A*
N2*A*—H2*A*⋯O1*B* ^i^	0.86	1.96	2.815 (3)	172
N2*B*—H2*B*⋯O1*A* ^ii^	0.86	1.96	2.810 (3)	169
C5*A*—H5*A*2⋯O1*B* ^i^	0.96	2.56	3.344 (4)	139
C5*A*—H5*A*3⋯O4*A* ^i^	0.96	2.54	3.477 (4)	164
C12*B*—H12*B*⋯O2*A* ^iii^	0.93	2.56	3.459 (4)	161

Symmetry codes: (i) 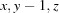; (ii) 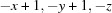; (iii) 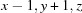.

**Table 5:** Experimental details

	**I**	**II**	**III**	**IV**
Crystal data				
Chemical formula	C_20_H_19_N_3_O_4_S	C_17_H_21_N_3_O_4_S·0.25H_2_O	C_16_H_19_N_3_O_4_S	C_22_H_21_N_3_O_4_S·0.5CHCl_3_
*M*_r_	397.44	367.93	349.40	483.16
Crystal system, space group	Triclinic, *P* 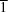	Triclinic, *P* 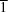	Triclinic, *P* 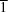	Triclinic, *P* 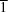
Temperature (K)	293	293	293	293
*a*, *b*, *c* (Å)	6.7559 (1), 16.9258 (2), 19.0611 (3)	6.7802 (1), 17.2671 (4), 17.3089 (4)	11.4150 (3), 12.4021 (3), 13.2305 (3)	10.7427 (1), 11.0828 (2), 20.8969 (3)
α, β, γ (°)	110.447 (1), 96.854 (2), 93.370 (1)	108.224 (1), 99.084 (1), 96.720 (1)	71.982 (1), 89.829 (1), 83.114 (1)	93.186 (1), 103.945 (4), 98.489 (2)
*V* (Å^3^)	2015.84 (5)	1870.50 (7)	1767.18 (8)	2377.39 (7)
*Z*	4	4	4	4
Radiation type	Mo *K*α	Mo *K*α	Mo *K*α	Mo *K*α
μ (mm^−1^)	0.19	0.20	0.21	0.34
Crystal size (mm)	0.30 × 0.25 × 0.20	0.30 × 0.25 × 0.20	0.25 × 0.24 × 0.20	0.30 × 0.25 × 0.20

Data collection				
Diffractometer	Bruker Kappa APEXII CCD	Bruker Kappa APEXII CCD	Bruker Kappa APEXII CCD	Bruker Kappa APEXII CCD
Absorption correction	Multi-scan (*SADABS*; Bruker, 2008 ▸)	Multi-scan (*SADABS*; Bruker, 2008 ▸)	Multi-scan (*SADABS*; Bruker, 2008 ▸)	Multi-scan (*SADABS*; Bruker, 2008 ▸)
*T*_min_, *T*_max_	0.648, 0.763	0.660, 0.746	0.756, 0.824	0.741, 0.856
No. of measured, independent and observed [*I* > 2σ(*I*)] reflections	27547, 7061, 4821	27060, 7680, 5737	26933, 7257, 5869	31719, 8335, 6495
*R*_int_	0.029	0.030	0.022	0.027
(sin θ/λ)_max_ (Å^−1^)	0.595	0.627	0.627	0.595

Refinement				
*R*[*F* ^2^ > 2σ(*F* ^2^)], *wR*(*F* ^2^), *S*	0.060, 0.226, 0.83	0.053, 0.169, 1.04	0.037, 0.106, 1.03	0.058, 0.195, 1.09
No. of reflections	7061	7680	7257	8335
No. of parameters	635	525	451	583
No. of restraints	523	242	0	0
H-atom treatment	H atoms treated by a mixture of independent and constrained refinement	H atoms treated by a mixture of independent and constrained refinement	H-atom parameters constrained	H-atom parameters constrained
Δρ_max_, Δρ_min_ (e Å^−3^)	0.38, −0.56	0.48, −0.38	0.24, −0.33	0.54, −0.60

Computer programs: *APEX2* and *SAINT* (Bruker, 2008 ▸), *SHELXS2018*/3 (Sheldrick, 2008 ▸), *SHELXL2018*/3 (Sheldrick, 2015 ▸), *ORTEP-3 for Windows* and *WinGX* (Farrugia, 2012 ▸), *Mercury* (Macrae *et al.*, 2008 ▸), *PLATON* (Spek, 2009 ▸) and *publCIF* (Westrip, 2010 ▸).

## supplementary crystallographic information

### 4-(5-Acetamido-3-acetyl-2-methyl-2,3-dihydro-1,3,4-thiadiazol-2-yl)phenyl benzoate (I) Crystal data

**Table:**

C_20_H_19_N_3_O_4_S	*Z* = 4
*M**_r_* = 397.44	*F*(000) = 832
Triclinic, *P*1	*D*_x_ = 1.310 Mg m^−^^3^
*a* = 6.7559 (1) Å	Mo *K*α radiation, λ = 0.71073 Å
*b* = 16.9258 (2) Å	Cell parameters from 7061 reflections
*c* = 19.0611 (3) Å	θ = 1.2–25.0°
α = 110.447 (1)°	µ = 0.19 mm^−^^1^
β = 96.854 (2)°	*T* = 293 K
γ = 93.370 (1)°	Block, colourless
*V* = 2015.84 (5) Å^3^	0.30 × 0.25 × 0.20 mm

### 4-(5-Acetamido-3-acetyl-2-methyl-2,3-dihydro-1,3,4-thiadiazol-2-yl)phenyl benzoate (I) Data collection

**Table:**

Bruker Kappa APEXII CCD diffractometer	4821 reflections with *I* > 2σ(*I*)
ω and φ scans	*R*_int_ = 0.029
Absorption correction: multi-scan (*SADABS*; Bruker, 2008)	θ_max_ = 25.0°, θ_min_ = 1.2°
*T*_min_ = 0.648, *T*_max_ = 0.763	*h* = −8→8
27547 measured reflections	*k* = −17→20
7061 independent reflections	*l* = −22→22

### 4-(5-Acetamido-3-acetyl-2-methyl-2,3-dihydro-1,3,4-thiadiazol-2-yl)phenyl benzoate (I) Refinement

**Table:**

Refinement on *F*^2^	Primary atom site location: structure-invariant direct methods
Least-squares matrix: full	Secondary atom site location: difference Fourier map
*R*[*F*^2^ > 2σ(*F*^2^)] = 0.060	Hydrogen site location: mixed
*wR*(*F*^2^) = 0.226	H atoms treated by a mixture of independent and constrained refinement
*S* = 0.83	*w* = 1/[σ^2^(*F*_o_^2^) + (0.173*P*)^2^ + 1.4455*P*] where *P* = (*F*_o_^2^ + 2*F*_c_^2^)/3
7061 reflections	(Δ/σ)_max_ < 0.001
635 parameters	Δρ_max_ = 0.38 e Å^−^^3^
523 restraints	Δρ_min_ = −0.56 e Å^−^^3^

### 4-(5-Acetamido-3-acetyl-2-methyl-2,3-dihydro-1,3,4-thiadiazol-2-yl)phenyl benzoate (I) Special details

**Table:**

Geometry. All esds (except the esd in the dihedral angle between two l.s. planes) are estimated using the full covariance matrix. The cell esds are taken into account individually in the estimation of esds in distances, angles and torsion angles; correlations between esds in cell parameters are only used when they are defined by crystal symmetry. An approximate (isotropic) treatment of cell esds is used for estimating esds involving l.s. planes.

### 4-(5-Acetamido-3-acetyl-2-methyl-2,3-dihydro-1,3,4-thiadiazol-2-yl)phenyl benzoate (I) Fractional atomic coordinates and isotropic or equivalent isotropic displacement parameters (Å^2^)

**Table:**

	*x*	*y*	*z*	*U*_iso_*/*U*_eq_	Occ. (<1)
C1A	0.2219 (6)	0.4068 (2)	0.0385 (2)	0.0656 (9)	
H1A1	0.177958	0.462086	0.058777	0.098*	
H1A2	0.184605	0.384459	−0.015565	0.098*	
H1A3	0.364971	0.410730	0.050786	0.098*	
C2A	0.1252 (5)	0.34900 (19)	0.07183 (18)	0.0543 (8)	
C6A	0.0955 (5)	0.20448 (19)	0.08168 (18)	0.0520 (7)	
C3A	0.3164 (4)	0.16342 (18)	−0.02111 (17)	0.0491 (7)	
C4A	0.4572 (5)	0.0414 (2)	−0.10248 (19)	0.0609 (9)	
C5A	0.6199 (7)	0.0180 (2)	−0.1495 (2)	0.0814 (11)	
H5A1	0.746604	0.029517	−0.117187	0.122*	
H5A2	0.621092	0.050733	−0.181743	0.122*	
H5A3	0.597016	−0.041245	−0.180053	0.122*	
C7A	−0.1299 (5)	0.1951 (2)	0.0779 (2)	0.0701 (10)	
H7A1	−0.169166	0.243320	0.116042	0.105*	
H7A2	−0.171080	0.144398	0.086486	0.105*	
H7A3	−0.192434	0.191664	0.028849	0.105*	
C8A	0.2146 (5)	0.22651 (19)	0.16091 (18)	0.0541 (8)	
C9A	0.4050 (5)	0.2035 (2)	0.1704 (2)	0.0637 (9)	
H9A	0.458678	0.170827	0.128259	0.076*	
C10A	0.5187 (7)	0.2281 (3)	0.2417 (3)	0.0815 (12)	
H10A	0.646125	0.211083	0.247511	0.098*	
C11A	0.4406 (9)	0.2779 (3)	0.3036 (2)	0.0942 (15)	
C12A	0.2516 (9)	0.3016 (3)	0.2952 (3)	0.1005 (15)	
H12A	0.198292	0.334662	0.337239	0.121*	
C13A	0.1417 (7)	0.2764 (3)	0.2247 (2)	0.0800 (11)	
H13A	0.014074	0.293392	0.219469	0.096*	
C5B	−0.3939 (6)	0.4189 (2)	0.1997 (3)	0.0781 (11)	
H5B1	−0.316132	0.410587	0.241635	0.117*	
H5B2	−0.362202	0.379959	0.153106	0.117*	
H5B3	−0.534022	0.409008	0.201844	0.117*	
C4B	−0.3463 (5)	0.5073 (2)	0.2036 (2)	0.0600 (8)	
C3B	−0.1030 (5)	0.60085 (19)	0.17747 (17)	0.0491 (7)	
C6B	0.0218 (5)	0.75618 (19)	0.21718 (18)	0.0544 (8)	
C7B	−0.0680 (6)	0.8261 (2)	0.1948 (2)	0.0736 (10)	
H7B1	0.037946	0.865664	0.193445	0.110*	
H7B2	−0.150634	0.855154	0.231292	0.110*	
H7B3	−0.147948	0.801636	0.145728	0.110*	
C8B	0.1840 (5)	0.78728 (19)	0.28650 (17)	0.0544 (8)	
C13B	0.2002 (7)	0.8682 (3)	0.3381 (2)	0.0900 (14)	
H13B	0.116198	0.906477	0.329469	0.108*	
C12B	0.3399 (9)	0.8936 (3)	0.4029 (3)	0.119 (2)	
H12B	0.347492	0.948328	0.438345	0.143*	
C11B	0.4662 (7)	0.8387 (3)	0.4147 (2)	0.0940 (14)	
C10B	0.4553 (6)	0.7583 (3)	0.3638 (2)	0.0811 (11)	
H10B	0.541883	0.720683	0.372222	0.097*	
C9B	0.3147 (6)	0.7332 (2)	0.2996 (2)	0.0699 (10)	
H9B	0.308075	0.678469	0.264329	0.084*	
C2B	0.2436 (5)	0.7107 (2)	0.11765 (19)	0.0609 (8)	
C1B	0.3273 (6)	0.6394 (2)	0.0626 (2)	0.0734 (10)	
H1B1	0.415555	0.661491	0.036299	0.110*	
H1B2	0.219721	0.601912	0.026726	0.110*	
H1B3	0.400488	0.608835	0.089090	0.110*	
N1A	0.1674 (4)	0.26824 (15)	0.04924 (14)	0.0506 (6)	
N2A	0.3109 (4)	0.24387 (15)	0.00068 (14)	0.0499 (6)	
N3A	0.4450 (4)	0.12653 (16)	−0.07072 (15)	0.0528 (6)	
H3A	0.515 (5)	0.152 (2)	−0.0914 (18)	0.051 (9)*	
N1B	0.0966 (4)	0.69192 (15)	0.15279 (14)	0.0532 (6)	
N2B	0.0369 (4)	0.60619 (15)	0.13948 (14)	0.0529 (6)	
N3B	−0.1812 (4)	0.52161 (17)	0.17374 (16)	0.0549 (7)	
H3B	−0.117 (6)	0.482 (3)	0.151 (2)	0.080 (13)*	
O1A	0.0081 (4)	0.37406 (15)	0.11807 (15)	0.0726 (7)	
O2A	0.3441 (5)	−0.00950 (15)	−0.09099 (17)	0.0862 (8)	
O1B	0.3065 (4)	0.78560 (15)	0.13218 (16)	0.0791 (8)	
O2B	−0.4457 (4)	0.56429 (18)	0.23256 (19)	0.0913 (9)	
S1A	0.15994 (13)	0.10508 (5)	0.01356 (5)	0.0586 (3)	
S1B	−0.18822 (13)	0.69320 (5)	0.23393 (6)	0.0656 (3)	
C14A	0.697 (2)	0.3397 (10)	0.4045 (6)	0.121 (3)	0.553 (5)
C15A	0.7452 (13)	0.3657 (5)	0.4903 (3)	0.113 (3)	0.553 (5)
C16A	0.9402 (12)	0.3664 (6)	0.5236 (5)	0.175 (4)	0.553 (5)
H16A	1.032983	0.338544	0.494131	0.210*	0.553 (5)
C17A	0.9967 (13)	0.4088 (7)	0.6011 (5)	0.181 (5)	0.553 (5)
H17A	1.127142	0.409308	0.623366	0.217*	0.553 (5)
C18A	0.8581 (17)	0.4505 (6)	0.6452 (3)	0.172 (5)	0.553 (5)
H18A	0.895807	0.478825	0.696988	0.207*	0.553 (5)
C19A	0.6630 (15)	0.4497 (6)	0.6119 (4)	0.155 (4)	0.553 (5)
H19A	0.570311	0.477579	0.641375	0.185*	0.553 (5)
C20A	0.6066 (11)	0.4073 (5)	0.5344 (4)	0.138 (4)	0.553 (5)
H20A	0.476148	0.406815	0.512140	0.165*	0.553 (5)
O3A	0.5068 (13)	0.3112 (7)	0.3822 (5)	0.102 (3)	0.553 (5)
O4A	0.754 (3)	0.3795 (13)	0.3656 (12)	0.280 (10)	0.553 (5)
C14C	0.799 (2)	0.3142 (11)	0.3741 (8)	0.121 (4)	0.447 (5)
C15C	0.8754 (14)	0.3580 (5)	0.4579 (4)	0.120 (3)	0.447 (5)
C16C	0.8530 (11)	0.3003 (5)	0.4941 (4)	0.091 (3)	0.447 (5)
H16C	0.754803	0.254222	0.473476	0.110*	0.447 (5)
C17C	0.9775 (14)	0.3113 (6)	0.5611 (4)	0.117 (4)	0.447 (5)
H17C	0.962484	0.272708	0.585356	0.141*	0.447 (5)
C18C	1.1243 (15)	0.3801 (7)	0.5920 (4)	0.157 (5)	0.447 (5)
H18C	1.207516	0.387540	0.636820	0.189*	0.447 (5)
C19C	1.1466 (16)	0.4379 (6)	0.5558 (6)	0.176 (5)	0.447 (5)
H19C	1.244869	0.483887	0.576403	0.212*	0.447 (5)
C20C	1.0222 (17)	0.4268 (6)	0.4887 (5)	0.162 (5)	0.447 (5)
H20C	1.037190	0.465403	0.464522	0.194*	0.447 (5)
O3C	0.5988 (14)	0.2887 (7)	0.3676 (6)	0.088 (3)	0.447 (5)
O4C	0.825 (2)	0.3587 (10)	0.3401 (9)	0.173 (6)	0.447 (5)
C14B	0.7656 (13)	0.8772 (6)	0.4908 (5)	0.083 (2)	0.661 (6)
C15B	0.8793 (11)	0.8962 (5)	0.5669 (3)	0.073 (2)	0.661 (6)
C16B	0.7909 (7)	0.8815 (4)	0.6240 (5)	0.085 (2)	0.661 (6)
H16B	0.656588	0.860085	0.614983	0.103*	0.661 (6)
C17B	0.9034 (15)	0.8987 (4)	0.6947 (4)	0.095 (2)	0.661 (6)
H17B	0.844281	0.888860	0.732942	0.114*	0.661 (6)
C18B	1.1042 (14)	0.9306 (5)	0.7082 (3)	0.092 (3)	0.661 (6)
H18B	1.179435	0.942175	0.755513	0.110*	0.661 (6)
C19B	1.1925 (6)	0.9453 (6)	0.6511 (5)	0.120 (3)	0.661 (6)
H19B	1.326900	0.966716	0.660124	0.144*	0.661 (6)
C20B	1.0801 (13)	0.9281 (6)	0.5804 (4)	0.110 (3)	0.661 (6)
H20B	1.139211	0.937942	0.542164	0.132*	0.661 (6)
O3B	0.5721 (8)	0.8573 (4)	0.4871 (3)	0.0906 (17)	0.661 (6)
O4B	0.8318 (9)	0.8830 (5)	0.4386 (3)	0.149 (3)	0.661 (6)
C14D	0.6548 (19)	0.8629 (9)	0.5293 (7)	0.083 (3)	0.339 (6)
C15D	0.8356 (19)	0.8907 (10)	0.5852 (9)	0.072 (3)	0.339 (6)
C16D	0.8392 (18)	0.8858 (10)	0.6566 (11)	0.093 (4)	0.339 (6)
H16D	0.725040	0.863845	0.669272	0.112*	0.339 (6)
C17D	1.013 (3)	0.9138 (10)	0.7091 (6)	0.093 (4)	0.339 (6)
H17D	1.015712	0.910545	0.756882	0.112*	0.339 (6)
C18D	1.1839 (19)	0.9466 (10)	0.6901 (8)	0.104 (5)	0.339 (6)
H18D	1.300469	0.965352	0.725262	0.124*	0.339 (6)
C19D	1.1804 (18)	0.9515 (11)	0.6187 (11)	0.113 (5)	0.339 (6)
H19D	1.294556	0.973460	0.606031	0.136*	0.339 (6)
C20D	1.006 (3)	0.9235 (12)	0.5662 (6)	0.096 (4)	0.339 (6)
H20D	1.003885	0.926762	0.518420	0.115*	0.339 (6)
O3D	0.6689 (19)	0.8790 (7)	0.4661 (6)	0.079 (3)	0.339 (6)
O4D	0.5080 (18)	0.8298 (9)	0.5407 (6)	0.139 (5)	0.339 (6)

### 4-(5-Acetamido-3-acetyl-2-methyl-2,3-dihydro-1,3,4-thiadiazol-2-yl)phenyl benzoate (I) Atomic displacement parameters (Å^2^)

**Table:**

	*U*^11^	*U*^22^	*U*^33^	*U*^12^	*U*^13^	*U*^23^
C1A	0.082 (2)	0.0410 (17)	0.081 (2)	0.0079 (16)	0.0187 (19)	0.0279 (16)
C2A	0.0599 (19)	0.0410 (17)	0.0619 (19)	0.0019 (14)	0.0021 (16)	0.0213 (14)
C6A	0.0518 (17)	0.0407 (16)	0.0654 (19)	−0.0048 (13)	0.0018 (14)	0.0252 (14)
C3A	0.0515 (17)	0.0377 (15)	0.0538 (17)	−0.0053 (13)	−0.0075 (13)	0.0176 (13)
C4A	0.075 (2)	0.0353 (16)	0.067 (2)	0.0000 (15)	−0.0002 (17)	0.0158 (14)
C5A	0.098 (3)	0.045 (2)	0.099 (3)	0.0096 (19)	0.028 (2)	0.0174 (19)
C7A	0.0519 (19)	0.062 (2)	0.097 (3)	−0.0067 (16)	0.0034 (18)	0.034 (2)
C8A	0.0619 (19)	0.0423 (16)	0.0616 (19)	−0.0037 (14)	0.0047 (15)	0.0261 (14)
C9A	0.065 (2)	0.057 (2)	0.072 (2)	−0.0024 (16)	−0.0038 (17)	0.0322 (17)
C10A	0.080 (3)	0.074 (3)	0.092 (3)	−0.016 (2)	−0.024 (2)	0.047 (2)
C11A	0.125 (4)	0.093 (3)	0.062 (3)	−0.035 (3)	−0.020 (3)	0.042 (2)
C12A	0.134 (5)	0.099 (4)	0.061 (3)	−0.004 (3)	0.009 (3)	0.023 (2)
C13A	0.084 (3)	0.082 (3)	0.073 (3)	0.008 (2)	0.013 (2)	0.027 (2)
C5B	0.073 (2)	0.064 (2)	0.107 (3)	−0.0018 (18)	0.020 (2)	0.043 (2)
C4B	0.0521 (18)	0.056 (2)	0.073 (2)	0.0013 (15)	0.0055 (16)	0.0262 (16)
C3B	0.0529 (17)	0.0436 (16)	0.0526 (16)	0.0054 (13)	0.0018 (14)	0.0212 (13)
C6B	0.0614 (19)	0.0404 (16)	0.0644 (19)	0.0082 (14)	0.0101 (15)	0.0217 (14)
C7B	0.090 (3)	0.052 (2)	0.083 (2)	0.0200 (18)	0.004 (2)	0.0297 (18)
C8B	0.0645 (19)	0.0435 (17)	0.0562 (17)	0.0099 (14)	0.0134 (15)	0.0171 (14)
C13B	0.109 (3)	0.061 (2)	0.081 (3)	0.032 (2)	−0.011 (2)	0.006 (2)
C12B	0.151 (5)	0.076 (3)	0.089 (3)	0.040 (3)	−0.028 (3)	−0.010 (2)
C11B	0.104 (3)	0.091 (3)	0.066 (2)	0.024 (3)	−0.011 (2)	0.007 (2)
C10B	0.082 (3)	0.079 (3)	0.077 (2)	0.027 (2)	−0.002 (2)	0.024 (2)
C9B	0.077 (2)	0.050 (2)	0.074 (2)	0.0149 (17)	0.0007 (18)	0.0138 (17)
C2B	0.077 (2)	0.0447 (18)	0.066 (2)	0.0031 (16)	0.0133 (17)	0.0249 (15)
C1B	0.085 (3)	0.054 (2)	0.084 (2)	0.0020 (18)	0.032 (2)	0.0234 (18)
N1A	0.0557 (15)	0.0389 (13)	0.0590 (15)	−0.0014 (11)	0.0023 (12)	0.0229 (11)
N2A	0.0564 (15)	0.0360 (13)	0.0583 (14)	0.0016 (11)	0.0047 (12)	0.0199 (11)
N3A	0.0611 (16)	0.0358 (13)	0.0615 (16)	0.0002 (12)	0.0074 (13)	0.0189 (12)
N1B	0.0659 (16)	0.0385 (13)	0.0576 (15)	0.0036 (11)	0.0121 (12)	0.0196 (11)
N2B	0.0680 (17)	0.0386 (13)	0.0544 (14)	0.0030 (12)	0.0096 (13)	0.0200 (11)
N3B	0.0613 (17)	0.0411 (14)	0.0644 (16)	0.0038 (12)	0.0119 (13)	0.0210 (12)
O1A	0.0828 (17)	0.0490 (13)	0.0951 (18)	0.0128 (12)	0.0308 (15)	0.0305 (12)
O2A	0.106 (2)	0.0383 (13)	0.108 (2)	−0.0094 (13)	0.0260 (17)	0.0185 (13)
O1B	0.1010 (19)	0.0472 (14)	0.0968 (19)	0.0003 (13)	0.0338 (15)	0.0304 (13)
O2B	0.0716 (17)	0.0696 (18)	0.141 (3)	0.0123 (14)	0.0387 (17)	0.0395 (18)
S1A	0.0671 (5)	0.0373 (4)	0.0682 (5)	−0.0104 (3)	0.0025 (4)	0.0201 (4)
S1B	0.0620 (5)	0.0485 (5)	0.0884 (7)	0.0101 (4)	0.0214 (5)	0.0231 (4)
C14A	0.124 (7)	0.141 (7)	0.074 (6)	−0.007 (6)	−0.023 (5)	0.021 (5)
C15A	0.120 (7)	0.113 (6)	0.078 (5)	−0.017 (5)	−0.029 (5)	0.020 (5)
C16A	0.181 (7)	0.174 (7)	0.131 (6)	−0.015 (7)	−0.035 (6)	0.029 (6)
C17A	0.193 (10)	0.194 (9)	0.106 (7)	−0.027 (8)	−0.068 (7)	0.029 (7)
C18A	0.220 (12)	0.186 (11)	0.090 (7)	−0.049 (10)	−0.044 (8)	0.058 (7)
C19A	0.208 (11)	0.159 (9)	0.089 (6)	−0.044 (8)	−0.003 (7)	0.053 (6)
C20A	0.188 (10)	0.130 (8)	0.089 (6)	−0.027 (7)	0.001 (6)	0.046 (6)
O3A	0.097 (6)	0.141 (7)	0.063 (4)	−0.025 (5)	−0.008 (4)	0.041 (4)
O4A	0.241 (16)	0.301 (16)	0.181 (14)	−0.081 (14)	−0.011 (12)	−0.026 (13)
C14C	0.113 (8)	0.165 (9)	0.072 (7)	−0.030 (7)	−0.020 (6)	0.043 (7)
C15C	0.143 (8)	0.130 (7)	0.075 (6)	−0.036 (6)	−0.035 (5)	0.046 (5)
C16C	0.100 (6)	0.097 (6)	0.072 (5)	0.004 (5)	−0.015 (5)	0.033 (5)
C17C	0.145 (8)	0.136 (8)	0.078 (6)	0.008 (7)	−0.018 (6)	0.059 (6)
C18C	0.195 (11)	0.151 (9)	0.113 (7)	−0.007 (8)	−0.052 (8)	0.059 (7)
C19C	0.221 (10)	0.163 (9)	0.125 (8)	−0.058 (8)	−0.062 (8)	0.067 (7)
C20C	0.199 (10)	0.142 (9)	0.126 (8)	−0.065 (8)	−0.077 (8)	0.071 (7)
O3C	0.087 (6)	0.116 (6)	0.063 (5)	−0.021 (5)	−0.014 (4)	0.047 (4)
O4C	0.114 (8)	0.234 (14)	0.110 (9)	−0.007 (8)	−0.009 (7)	−0.001 (9)
C14B	0.079 (5)	0.095 (4)	0.079 (5)	0.005 (4)	0.006 (4)	0.038 (4)
C15B	0.059 (4)	0.073 (4)	0.083 (4)	−0.001 (3)	0.001 (3)	0.027 (3)
C16B	0.088 (4)	0.085 (4)	0.077 (5)	0.000 (4)	−0.010 (4)	0.029 (4)
C17B	0.089 (6)	0.099 (5)	0.086 (5)	−0.009 (4)	−0.013 (4)	0.030 (4)
C18B	0.085 (6)	0.105 (5)	0.078 (4)	0.004 (5)	−0.004 (4)	0.031 (4)
C19B	0.085 (5)	0.163 (6)	0.092 (7)	−0.002 (5)	−0.014 (5)	0.033 (6)
C20B	0.082 (6)	0.150 (6)	0.087 (5)	−0.008 (5)	−0.009 (4)	0.039 (5)
O3B	0.076 (3)	0.119 (4)	0.062 (3)	0.006 (3)	0.000 (3)	0.018 (3)
O4B	0.100 (4)	0.248 (8)	0.107 (4)	−0.043 (4)	−0.012 (3)	0.092 (5)
C14D	0.078 (6)	0.089 (6)	0.078 (6)	0.006 (6)	0.014 (6)	0.027 (5)
C15D	0.078 (6)	0.074 (6)	0.069 (6)	0.007 (6)	0.002 (6)	0.034 (5)
C16D	0.083 (7)	0.100 (7)	0.089 (8)	−0.006 (6)	−0.026 (7)	0.039 (7)
C17D	0.085 (9)	0.103 (7)	0.087 (6)	−0.009 (8)	−0.011 (7)	0.041 (6)
C18D	0.082 (8)	0.134 (9)	0.082 (9)	0.008 (8)	−0.012 (8)	0.031 (8)
C19D	0.088 (8)	0.151 (8)	0.084 (9)	0.002 (7)	−0.019 (7)	0.034 (8)
C20D	0.069 (8)	0.123 (7)	0.085 (6)	0.007 (7)	−0.009 (6)	0.030 (6)
O3D	0.069 (7)	0.095 (6)	0.071 (6)	−0.004 (5)	0.003 (5)	0.033 (5)
O4D	0.114 (8)	0.198 (12)	0.100 (8)	−0.038 (8)	0.002 (6)	0.062 (8)

### 4-(5-Acetamido-3-acetyl-2-methyl-2,3-dihydro-1,3,4-thiadiazol-2-yl)phenyl benzoate (I) Geometric parameters (Å, º)

**Table:**

C1A—C2A	1.499 (5)	C2B—C1B	1.488 (5)
C1A—H1A1	0.9600	C1B—H1B1	0.9600
C1A—H1A2	0.9600	C1B—H1B2	0.9600
C1A—H1A3	0.9600	C1B—H1B3	0.9600
C2A—O1A	1.236 (4)	N1A—N2A	1.400 (4)
C2A—N1A	1.341 (4)	N3A—H3A	0.84 (3)
C6A—N1A	1.503 (4)	N1B—N2B	1.408 (3)
C6A—C7A	1.512 (5)	N3B—H3B	0.85 (4)
C6A—C8A	1.533 (4)	C14A—O4A	1.239 (16)
C6A—S1A	1.844 (3)	C14A—O3A	1.312 (13)
C3A—N2A	1.282 (4)	C14A—C15A	1.526 (11)
C3A—N3A	1.373 (4)	C15A—C16A	1.3900
C3A—S1A	1.740 (3)	C15A—C20A	1.3900
C4A—O2A	1.212 (4)	C16A—C17A	1.3900
C4A—N3A	1.366 (4)	C16A—H16A	0.9300
C4A—C5A	1.485 (5)	C17A—C18A	1.3900
C5A—H5A1	0.9600	C17A—H17A	0.9300
C5A—H5A2	0.9600	C18A—C19A	1.3900
C5A—H5A3	0.9600	C18A—H18A	0.9300
C7A—H7A1	0.9600	C19A—C20A	1.3900
C7A—H7A2	0.9600	C19A—H19A	0.9300
C7A—H7A3	0.9600	C20A—H20A	0.9300
C8A—C9A	1.376 (5)	C14C—O4C	1.171 (15)
C8A—C13A	1.382 (5)	C14C—O3C	1.370 (15)
C9A—C10A	1.389 (5)	C14C—C15C	1.517 (13)
C9A—H9A	0.9300	C15C—C16C	1.3900
C10A—C11A	1.374 (7)	C15C—C20C	1.3900
C10A—H10A	0.9300	C16C—C17C	1.3900
C11A—C12A	1.369 (7)	C16C—H16C	0.9300
C11A—O3A	1.407 (10)	C17C—C18C	1.3900
C11A—O3C	1.473 (11)	C17C—H17C	0.9300
C12A—C13A	1.364 (6)	C18C—C19C	1.3900
C12A—H12A	0.9300	C18C—H18C	0.9300
C13A—H13A	0.9300	C19C—C20C	1.3900
C5B—C4B	1.487 (5)	C19C—H19C	0.9300
C5B—H5B1	0.9600	C20C—H20C	0.9300
C5B—H5B2	0.9600	C14B—O4B	1.170 (9)
C5B—H5B3	0.9600	C14B—O3B	1.318 (9)
C4B—O2B	1.216 (4)	C14B—C15B	1.476 (9)
C4B—N3B	1.357 (4)	C15B—C16B	1.3900
C3B—N2B	1.275 (4)	C15B—C20B	1.3900
C3B—N3B	1.387 (4)	C16B—C17B	1.3900
C3B—S1B	1.735 (3)	C16B—H16B	0.9300
C6B—N1B	1.492 (4)	C17B—C18B	1.3900
C6B—C8B	1.525 (5)	C17B—H17B	0.9300
C6B—C7B	1.525 (4)	C18B—C19B	1.3900
C6B—S1B	1.849 (3)	C18B—H18B	0.9300
C7B—H7B1	0.9600	C19B—C20B	1.3900
C7B—H7B2	0.9600	C19B—H19B	0.9300
C7B—H7B3	0.9600	C20B—H20B	0.9300
C8B—C13B	1.368 (5)	C14D—O4D	1.190 (13)
C8B—C9B	1.372 (5)	C14D—O3D	1.336 (13)
C13B—C12B	1.380 (6)	C14D—C15D	1.456 (13)
C13B—H13B	0.9300	C15D—C16D	1.3900
C12B—C11B	1.355 (6)	C15D—C20D	1.3900
C12B—H12B	0.9300	C16D—C17D	1.3900
C11B—C10B	1.360 (6)	C16D—H16D	0.9300
C11B—O3B	1.396 (7)	C17D—C18D	1.3900
C11B—O3D	1.551 (13)	C17D—H17D	0.9300
C10B—C9B	1.376 (5)	C18D—C19D	1.3900
C10B—H10B	0.9300	C18D—H18D	0.9300
C9B—H9B	0.9300	C19D—C20D	1.3900
C2B—O1B	1.236 (4)	C19D—H19D	0.9300
C2B—N1B	1.344 (4)	C20D—H20D	0.9300
			
C2A—C1A—H1A1	109.5	H1B2—C1B—H1B3	109.5
C2A—C1A—H1A2	109.5	C2A—N1A—N2A	118.7 (2)
H1A1—C1A—H1A2	109.5	C2A—N1A—C6A	124.3 (3)
C2A—C1A—H1A3	109.5	N2A—N1A—C6A	116.3 (2)
H1A1—C1A—H1A3	109.5	C3A—N2A—N1A	110.0 (2)
H1A2—C1A—H1A3	109.5	C4A—N3A—C3A	125.3 (3)
O1A—C2A—N1A	120.5 (3)	C4A—N3A—H3A	109 (2)
O1A—C2A—C1A	121.6 (3)	C3A—N3A—H3A	125 (2)
N1A—C2A—C1A	117.9 (3)	C2B—N1B—N2B	118.8 (3)
N1A—C6A—C7A	112.8 (3)	C2B—N1B—C6B	123.3 (3)
N1A—C6A—C8A	108.2 (2)	N2B—N1B—C6B	116.6 (2)
C7A—C6A—C8A	115.0 (3)	C3B—N2B—N1B	109.9 (2)
N1A—C6A—S1A	101.4 (2)	C4B—N3B—C3B	124.4 (3)
C7A—C6A—S1A	106.6 (2)	C4B—N3B—H3B	122 (3)
C8A—C6A—S1A	112.1 (2)	C3B—N3B—H3B	114 (3)
N2A—C3A—N3A	119.0 (3)	C3A—S1A—C6A	89.90 (14)
N2A—C3A—S1A	118.5 (2)	C3B—S1B—C6B	89.66 (14)
N3A—C3A—S1A	122.5 (2)	O4A—C14A—O3A	110.2 (13)
O2A—C4A—N3A	121.1 (3)	O4A—C14A—C15A	126.8 (15)
O2A—C4A—C5A	124.0 (3)	O3A—C14A—C15A	109.7 (10)
N3A—C4A—C5A	114.8 (3)	C16A—C15A—C20A	120.0
C4A—C5A—H5A1	109.5	C16A—C15A—C14A	119.7 (7)
C4A—C5A—H5A2	109.5	C20A—C15A—C14A	118.4 (8)
H5A1—C5A—H5A2	109.5	C15A—C16A—C17A	120.0
C4A—C5A—H5A3	109.5	C15A—C16A—H16A	120.0
H5A1—C5A—H5A3	109.5	C17A—C16A—H16A	120.0
H5A2—C5A—H5A3	109.5	C18A—C17A—C16A	120.0
C6A—C7A—H7A1	109.5	C18A—C17A—H17A	120.0
C6A—C7A—H7A2	109.5	C16A—C17A—H17A	120.0
H7A1—C7A—H7A2	109.5	C17A—C18A—C19A	120.0
C6A—C7A—H7A3	109.5	C17A—C18A—H18A	120.0
H7A1—C7A—H7A3	109.5	C19A—C18A—H18A	120.0
H7A2—C7A—H7A3	109.5	C20A—C19A—C18A	120.0
C9A—C8A—C13A	117.6 (3)	C20A—C19A—H19A	120.0
C9A—C8A—C6A	121.0 (3)	C18A—C19A—H19A	120.0
C13A—C8A—C6A	121.1 (3)	C19A—C20A—C15A	120.0
C8A—C9A—C10A	121.2 (4)	C19A—C20A—H20A	120.0
C8A—C9A—H9A	119.4	C15A—C20A—H20A	120.0
C10A—C9A—H9A	119.4	C14A—O3A—C11A	117.1 (8)
C11A—C10A—C9A	119.2 (4)	O4C—C14C—O3C	111.6 (14)
C11A—C10A—H10A	120.4	O4C—C14C—C15C	111.8 (14)
C9A—C10A—H10A	120.4	O3C—C14C—C15C	107.0 (12)
C12A—C11A—C10A	120.3 (4)	C16C—C15C—C20C	120.0
C12A—C11A—O3A	104.9 (6)	C16C—C15C—C14C	109.1 (8)
C10A—C11A—O3A	134.7 (6)	C20C—C15C—C14C	125.1 (7)
C12A—C11A—O3C	136.1 (6)	C15C—C16C—C17C	120.0
C10A—C11A—O3C	103.5 (6)	C15C—C16C—H16C	120.0
C13A—C12A—C11A	119.7 (5)	C17C—C16C—H16C	120.0
C13A—C12A—H12A	120.2	C16C—C17C—C18C	120.0
C11A—C12A—H12A	120.2	C16C—C17C—H17C	120.0
C12A—C13A—C8A	121.9 (4)	C18C—C17C—H17C	120.0
C12A—C13A—H13A	119.0	C17C—C18C—C19C	120.0
C8A—C13A—H13A	119.0	C17C—C18C—H18C	120.0
C4B—C5B—H5B1	109.5	C19C—C18C—H18C	120.0
C4B—C5B—H5B2	109.5	C20C—C19C—C18C	120.0
H5B1—C5B—H5B2	109.5	C20C—C19C—H19C	120.0
C4B—C5B—H5B3	109.5	C18C—C19C—H19C	120.0
H5B1—C5B—H5B3	109.5	C19C—C20C—C15C	120.0
H5B2—C5B—H5B3	109.5	C19C—C20C—H20C	120.0
O2B—C4B—N3B	121.3 (3)	C15C—C20C—H20C	120.0
O2B—C4B—C5B	122.8 (3)	C14C—O3C—C11A	127.5 (9)
N3B—C4B—C5B	115.8 (3)	O4B—C14B—O3B	121.2 (8)
N2B—C3B—N3B	119.3 (3)	O4B—C14B—C15B	125.6 (8)
N2B—C3B—S1B	119.1 (2)	O3B—C14B—C15B	113.1 (7)
N3B—C3B—S1B	121.5 (2)	C16B—C15B—C20B	120.0
N1B—C6B—C8B	110.0 (2)	C16B—C15B—C14B	121.5 (8)
N1B—C6B—C7B	112.3 (3)	C20B—C15B—C14B	118.5 (8)
C8B—C6B—C7B	114.9 (3)	C17B—C16B—C15B	120.0
N1B—C6B—S1B	102.0 (2)	C17B—C16B—H16B	120.0
C8B—C6B—S1B	110.0 (2)	C15B—C16B—H16B	120.0
C7B—C6B—S1B	106.8 (2)	C16B—C17B—C18B	120.0
C6B—C7B—H7B1	109.5	C16B—C17B—H17B	120.0
C6B—C7B—H7B2	109.5	C18B—C17B—H17B	120.0
H7B1—C7B—H7B2	109.5	C19B—C18B—C17B	120.0
C6B—C7B—H7B3	109.5	C19B—C18B—H18B	120.0
H7B1—C7B—H7B3	109.5	C17B—C18B—H18B	120.0
H7B2—C7B—H7B3	109.5	C18B—C19B—C20B	120.0
C13B—C8B—C9B	118.3 (3)	C18B—C19B—H19B	120.0
C13B—C8B—C6B	121.5 (3)	C20B—C19B—H19B	120.0
C9B—C8B—C6B	120.2 (3)	C19B—C20B—C15B	120.0
C8B—C13B—C12B	120.7 (4)	C19B—C20B—H20B	120.0
C8B—C13B—H13B	119.6	C15B—C20B—H20B	120.0
C12B—C13B—H13B	119.6	C14B—O3B—C11B	112.7 (7)
C11B—C12B—C13B	119.8 (4)	O4D—C14D—O3D	124.1 (14)
C11B—C12B—H12B	120.1	O4D—C14D—C15D	121.6 (14)
C13B—C12B—H12B	120.1	O3D—C14D—C15D	114.3 (13)
C12B—C11B—C10B	120.7 (4)	C16D—C15D—C20D	120.0
C12B—C11B—O3B	118.9 (4)	C16D—C15D—C14D	121.3 (16)
C10B—C11B—O3B	118.4 (5)	C20D—C15D—C14D	118.7 (16)
C12B—C11B—O3D	116.0 (6)	C17D—C16D—C15D	120.0
C10B—C11B—O3D	118.4 (6)	C17D—C16D—H16D	120.0
C11B—C10B—C9B	119.1 (4)	C15D—C16D—H16D	120.0
C11B—C10B—H10B	120.4	C16D—C17D—C18D	120.0
C9B—C10B—H10B	120.4	C16D—C17D—H17D	120.0
C8B—C9B—C10B	121.4 (3)	C18D—C17D—H17D	120.0
C8B—C9B—H9B	119.3	C19D—C18D—C17D	120.0
C10B—C9B—H9B	119.3	C19D—C18D—H18D	120.0
O1B—C2B—N1B	119.7 (3)	C17D—C18D—H18D	120.0
O1B—C2B—C1B	122.1 (3)	C18D—C19D—C20D	120.0
N1B—C2B—C1B	118.2 (3)	C18D—C19D—H19D	120.0
C2B—C1B—H1B1	109.5	C20D—C19D—H19D	120.0
C2B—C1B—H1B2	109.5	C19D—C20D—C15D	120.0
H1B1—C1B—H1B2	109.5	C19D—C20D—H20D	120.0
C2B—C1B—H1B3	109.5	C15D—C20D—H20D	120.0
H1B1—C1B—H1B3	109.5	C14D—O3D—C11B	104.3 (10)
			
N1A—C6A—C8A—C9A	−80.2 (3)	N3A—C3A—S1A—C6A	170.4 (3)
C7A—C6A—C8A—C9A	152.7 (3)	N1A—C6A—S1A—C3A	15.58 (19)
S1A—C6A—C8A—C9A	30.8 (3)	C7A—C6A—S1A—C3A	133.8 (2)
N1A—C6A—C8A—C13A	94.1 (4)	C8A—C6A—S1A—C3A	−99.6 (2)
C7A—C6A—C8A—C13A	−33.0 (4)	N2B—C3B—S1B—C6B	−9.2 (3)
S1A—C6A—C8A—C13A	−155.0 (3)	N3B—C3B—S1B—C6B	169.8 (3)
C13A—C8A—C9A—C10A	1.2 (5)	N1B—C6B—S1B—C3B	13.5 (2)
C6A—C8A—C9A—C10A	175.7 (3)	C8B—C6B—S1B—C3B	−103.2 (2)
C8A—C9A—C10A—C11A	−1.4 (6)	C7B—C6B—S1B—C3B	131.5 (2)
C9A—C10A—C11A—C12A	1.2 (6)	O4A—C14A—C15A—C16A	−68 (2)
C9A—C10A—C11A—O3A	177.2 (7)	O3A—C14A—C15A—C16A	155.3 (9)
C9A—C10A—C11A—O3C	178.7 (5)	O4A—C14A—C15A—C20A	96 (2)
C10A—C11A—C12A—C13A	−0.8 (7)	O3A—C14A—C15A—C20A	−40.2 (14)
O3A—C11A—C12A—C13A	−177.9 (6)	C20A—C15A—C16A—C17A	0.0
O3C—C11A—C12A—C13A	−177.4 (7)	C14A—C15A—C16A—C17A	164.3 (10)
C11A—C12A—C13A—C8A	0.7 (7)	C15A—C16A—C17A—C18A	0.0
C9A—C8A—C13A—C12A	−0.9 (6)	C16A—C17A—C18A—C19A	0.0
C6A—C8A—C13A—C12A	−175.4 (4)	C17A—C18A—C19A—C20A	0.0
N1B—C6B—C8B—C13B	148.5 (4)	C18A—C19A—C20A—C15A	0.0
C7B—C6B—C8B—C13B	20.6 (5)	C16A—C15A—C20A—C19A	0.0
S1B—C6B—C8B—C13B	−100.0 (4)	C14A—C15A—C20A—C19A	−164.5 (9)
N1B—C6B—C8B—C9B	−33.4 (4)	O4A—C14A—O3A—C11A	41 (2)
C7B—C6B—C8B—C9B	−161.2 (3)	C15A—C14A—O3A—C11A	−174.9 (9)
S1B—C6B—C8B—C9B	78.1 (4)	C12A—C11A—O3A—C14A	−143.6 (10)
C9B—C8B—C13B—C12B	−2.2 (7)	C10A—C11A—O3A—C14A	40.0 (14)
C6B—C8B—C13B—C12B	175.9 (5)	O4C—C14C—C15C—C16C	−175.6 (14)
C8B—C13B—C12B—C11B	1.7 (9)	O3C—C14C—C15C—C16C	61.9 (14)
C13B—C12B—C11B—C10B	−0.7 (10)	O4C—C14C—C15C—C20C	−23 (2)
C13B—C12B—C11B—O3B	−164.8 (6)	O3C—C14C—C15C—C20C	−145.4 (9)
C13B—C12B—C11B—O3D	154.3 (7)	C20C—C15C—C16C—C17C	0.0
C12B—C11B—C10B—C9B	0.3 (8)	C14C—C15C—C16C—C17C	154.3 (10)
O3B—C11B—C10B—C9B	164.5 (5)	C15C—C16C—C17C—C18C	0.0
O3D—C11B—C10B—C9B	−154.2 (6)	C16C—C17C—C18C—C19C	0.0
C13B—C8B—C9B—C10B	1.8 (6)	C17C—C18C—C19C—C20C	0.0
C6B—C8B—C9B—C10B	−176.4 (4)	C18C—C19C—C20C—C15C	0.0
C11B—C10B—C9B—C8B	−0.8 (7)	C16C—C15C—C20C—C19C	0.0
O1A—C2A—N1A—N2A	−175.8 (3)	C14C—C15C—C20C—C19C	−149.9 (12)
C1A—C2A—N1A—N2A	5.0 (4)	O4C—C14C—O3C—C11A	29 (2)
O1A—C2A—N1A—C6A	−5.8 (5)	C15C—C14C—O3C—C11A	151.5 (11)
C1A—C2A—N1A—C6A	175.1 (3)	C12A—C11A—O3C—C14C	−132.4 (13)
C7A—C6A—N1A—C2A	55.1 (4)	C10A—C11A—O3C—C14C	50.7 (15)
C8A—C6A—N1A—C2A	−73.2 (4)	O4B—C14B—C15B—C16B	−175.2 (9)
S1A—C6A—N1A—C2A	168.7 (2)	O3B—C14B—C15B—C16B	8.9 (10)
C7A—C6A—N1A—N2A	−134.7 (3)	O4B—C14B—C15B—C20B	3.8 (13)
C8A—C6A—N1A—N2A	97.0 (3)	O3B—C14B—C15B—C20B	−172.2 (6)
S1A—C6A—N1A—N2A	−21.0 (3)	C20B—C15B—C16B—C17B	0.0
N3A—C3A—N2A—N1A	178.5 (2)	C14B—C15B—C16B—C17B	178.9 (8)
S1A—C3A—N2A—N1A	−1.5 (3)	C15B—C16B—C17B—C18B	0.0
C2A—N1A—N2A—C3A	−173.3 (3)	C16B—C17B—C18B—C19B	0.0
C6A—N1A—N2A—C3A	15.9 (3)	C17B—C18B—C19B—C20B	0.0
O2A—C4A—N3A—C3A	3.5 (5)	C18B—C19B—C20B—C15B	0.0
C5A—C4A—N3A—C3A	−175.3 (3)	C16B—C15B—C20B—C19B	0.0
N2A—C3A—N3A—C4A	−175.4 (3)	C14B—C15B—C20B—C19B	−178.9 (7)
S1A—C3A—N3A—C4A	4.6 (4)	O4B—C14B—O3B—C11B	4.4 (12)
O1B—C2B—N1B—N2B	−175.6 (3)	C15B—C14B—O3B—C11B	−179.4 (6)
C1B—C2B—N1B—N2B	3.8 (5)	C12B—C11B—O3B—C14B	−110.0 (7)
O1B—C2B—N1B—C6B	−8.8 (5)	C10B—C11B—O3B—C14B	85.6 (8)
C1B—C2B—N1B—C6B	170.5 (3)	O4D—C14D—C15D—C16D	−5 (2)
C8B—C6B—N1B—C2B	−67.7 (4)	O3D—C14D—C15D—C16D	173.9 (11)
C7B—C6B—N1B—C2B	61.6 (4)	O4D—C14D—C15D—C20D	175.6 (14)
S1B—C6B—N1B—C2B	175.6 (3)	O3D—C14D—C15D—C20D	−5.3 (17)
C8B—C6B—N1B—N2B	99.4 (3)	C20D—C15D—C16D—C17D	0.0
C7B—C6B—N1B—N2B	−131.4 (3)	C14D—C15D—C16D—C17D	−179.2 (15)
S1B—C6B—N1B—N2B	−17.4 (3)	C15D—C16D—C17D—C18D	0.0
N3B—C3B—N2B—N1B	−178.7 (3)	C16D—C17D—C18D—C19D	0.0
S1B—C3B—N2B—N1B	0.3 (3)	C17D—C18D—C19D—C20D	0.0
C2B—N1B—N2B—C3B	179.9 (3)	C18D—C19D—C20D—C15D	0.0
C6B—N1B—N2B—C3B	12.3 (4)	C16D—C15D—C20D—C19D	0.0
O2B—C4B—N3B—C3B	4.4 (5)	C14D—C15D—C20D—C19D	179.2 (15)
C5B—C4B—N3B—C3B	−174.5 (3)	O4D—C14D—O3D—C11B	−4.6 (19)
N2B—C3B—N3B—C4B	−171.1 (3)	C15D—C14D—O3D—C11B	176.4 (11)
S1B—C3B—N3B—C4B	10.0 (4)	C12B—C11B—O3D—C14D	110.4 (10)
N2A—C3A—S1A—C6A	−9.6 (2)	C10B—C11B—O3D—C14D	−94.0 (10)

### 4-(5-Acetamido-3-acetyl-2-methyl-2,3-dihydro-1,3,4-thiadiazol-2-yl)phenyl benzoate (I) Hydrogen-bond geometry (Å, º)

*Cg*2, *Cg*3 and *Cg*6 are the centroids of the C8*A*–C13*A*, C15*A*–C20*A* and C8*B*–C13*B* rings, respectively.

**Table:**

*D*—H···*A*	*D*—H	H···*A*	*D*···*A*	*D*—H···*A*
N3*A*—H3*A*···O1*B*^i^	0.84 (4)	1.96 (3)	2.792 (4)	175 (3)
N3*B*—H3*B*···O1*A*	0.84 (4)	1.99 (5)	2.801 (4)	163 (4)
C5*A*—H5*A*2···O1*B*^i^	0.96	2.59	3.226 (5)	124
C7*A*—H7*A*2···O2*A*^ii^	0.96	2.54	3.482 (5)	168
C9*B*—H9*B*···O2*B*^iii^	0.93	2.58	3.303 (5)	135
C5*B*—H5*B*1···O4*A*^iv^	0.96	2.59	3.50 (2)	158
C5*B*—H5*B*1···O4*C*^iv^	0.96	2.45	3.395 (17)	166
C12*A*—H12*A*···O4*C*^iv^	0.93	2.58	3.211 (15)	125
C17*B*—H17*B*···*Cg*2^v^	0.93	2.91	3.664 (8)	139
C17*C*—H17*C*···*Cg*6^v^	0.93	2.98	3.776 (10)	145
C20*C*—H20*C*···*Cg*3^vi^	0.93	2.64	3.521 (11)	159

Symmetry codes: (i) −*x*+1, −*y*+1, −*z*; (ii) −*x*, −*y*, −*z*; (iii) *x*+1, *y*, *z*; (iv) *x*−1, *y*, *z*; (v) −*x*+1, −*y*+1, −*z*+1; (vi) −*x*+2, −*y*+1, −*z*+1.

### 4-(5-Acetamido-3-acetyl-2-methyl-2,3-dihydro-1,3,4-thiadiazol-2-yl)phenyl 2-methylpropanoate 0.25-hydrate (II) Crystal data

**Table:**

C_17_H_21_N_3_O_4_S·0.25H_2_O	*Z* = 4
*M**_r_* = 367.93	*F*(000) = 778
Triclinic, *P*1	*D*_x_ = 1.307 Mg m^−^^3^
*a* = 6.7802 (1) Å	Mo *K*α radiation, λ = 0.71073 Å
*b* = 17.2671 (4) Å	Cell parameters from 7680 reflections
*c* = 17.3089 (4) Å	θ = 2.4–25.5°
α = 108.224 (1)°	µ = 0.20 mm^−^^1^
β = 99.084 (1)°	*T* = 293 K
γ = 96.720 (1)°	BLOCK, colourless
*V* = 1870.50 (7) Å^3^	0.30 × 0.25 × 0.20 mm

### 4-(5-Acetamido-3-acetyl-2-methyl-2,3-dihydro-1,3,4-thiadiazol-2-yl)phenyl 2-methylpropanoate 0.25-hydrate (II) Data collection

**Table:**

Bruker Kappa APEXII CCD diffractometer	7680 independent reflections
Radiation source: fine-focus sealed tube	5737 reflections with *I* > 2σ(*I*)
Graphite monochromator	*R*_int_ = 0.030
ω and φ scans	θ_max_ = 26.5°, θ_min_ = 2.4°
Absorption correction: multi-scan (*SADABS*; Bruker, 2008)	*h* = −7→8
*T*_min_ = 0.660, *T*_max_ = 0.746	*k* = −21→21
27060 measured reflections	*l* = −21→21

### 4-(5-Acetamido-3-acetyl-2-methyl-2,3-dihydro-1,3,4-thiadiazol-2-yl)phenyl 2-methylpropanoate 0.25-hydrate (II) Refinement

**Table:**

Refinement on *F*^2^	Primary atom site location: structure-invariant direct methods
Least-squares matrix: full	Secondary atom site location: difference Fourier map
*R*[*F*^2^ > 2σ(*F*^2^)] = 0.053	Hydrogen site location: mixed
*wR*(*F*^2^) = 0.169	H atoms treated by a mixture of independent and constrained refinement
*S* = 1.04	*w* = 1/[σ^2^(*F*_o_^2^) + (0.0901*P*)^2^ + 0.734*P*] where *P* = (*F*_o_^2^ + 2*F*_c_^2^)/3
7680 reflections	(Δ/σ)_max_ = 0.002
525 parameters	Δρ_max_ = 0.48 e Å^−^^3^
242 restraints	Δρ_min_ = −0.38 e Å^−^^3^

### 4-(5-Acetamido-3-acetyl-2-methyl-2,3-dihydro-1,3,4-thiadiazol-2-yl)phenyl 2-methylpropanoate 0.25-hydrate (II) Special details

**Table:**

Geometry. All esds (except the esd in the dihedral angle between two l.s. planes) are estimated using the full covariance matrix. The cell esds are taken into account individually in the estimation of esds in distances, angles and torsion angles; correlations between esds in cell parameters are only used when they are defined by crystal symmetry. An approximate (isotropic) treatment of cell esds is used for estimating esds involving l.s. planes.

### 4-(5-Acetamido-3-acetyl-2-methyl-2,3-dihydro-1,3,4-thiadiazol-2-yl)phenyl 2-methylpropanoate 0.25-hydrate (II) Fractional atomic coordinates and isotropic or equivalent isotropic displacement parameters (Å^2^)

**Table:**

	*x*	*y*	*z*	*U*_iso_*/*U*_eq_	Occ. (<1)
C10B	0.6406 (7)	0.1228 (2)	0.0560 (2)	0.1066 (13)	
H10B	0.6521	0.0767	0.0124	0.128*	
C11B	0.4800 (6)	0.1636 (3)	0.0486 (2)	0.1023 (12)	
C12B	0.4677 (5)	0.2347 (2)	0.1099 (2)	0.0826 (9)	
H12B	0.3620	0.2633	0.1030	0.099*	
C13B	0.6142 (4)	0.26296 (18)	0.18174 (17)	0.0650 (7)	
H13B	0.6094	0.3123	0.2227	0.078*	
C1B	0.6525 (4)	0.36366 (16)	0.43978 (17)	0.0646 (7)	
H1B1	0.5739	0.3916	0.4095	0.097*	
H1B2	0.7689	0.4020	0.4766	0.097*	
H1B3	0.5709	0.3422	0.4715	0.097*	
C1A	0.7780 (4)	0.59164 (14)	0.45336 (16)	0.0571 (6)	
H1A1	0.8151	0.5379	0.4347	0.086*	
H1A2	0.6334	0.5865	0.4382	0.086*	
H1A3	0.8207	0.6154	0.5127	0.086*	
C2A	0.8791 (4)	0.64657 (13)	0.41369 (14)	0.0474 (5)	
C2B	0.7206 (4)	0.29410 (15)	0.38025 (15)	0.0528 (6)	
C3A	0.7214 (3)	0.82955 (12)	0.50833 (13)	0.0411 (5)	
C3B	1.0722 (3)	0.40326 (13)	0.31134 (13)	0.0420 (5)	
C4A	0.6056 (4)	0.95076 (14)	0.59064 (16)	0.0562 (6)	
C4B	1.3254 (4)	0.49275 (15)	0.27918 (15)	0.0517 (5)	
C5A	0.4525 (5)	0.97664 (17)	0.6418 (2)	0.0774 (8)	
H5A1	0.4990	1.0326	0.6787	0.116*	
H5A2	0.4339	0.9407	0.6736	0.116*	
H5A3	0.3259	0.9732	0.6060	0.116*	
C5B	1.3775 (4)	0.57778 (17)	0.27520 (19)	0.0656 (7)	
H5B1	1.5152	0.5870	0.2685	0.098*	
H5B2	1.3627	0.6181	0.3257	0.098*	
H5B3	1.2880	0.5828	0.2289	0.098*	
C6B	0.9311 (4)	0.25124 (13)	0.27334 (14)	0.0477 (5)	
C6A	0.9260 (3)	0.78654 (13)	0.39637 (14)	0.0440 (5)	
C7A	1.1542 (4)	0.79518 (16)	0.40080 (18)	0.0608 (6)	
H7A1	1.2227	0.8052	0.4569	0.091*	
H7A2	1.1997	0.8407	0.3836	0.091*	
H7A3	1.1839	0.7450	0.3648	0.091*	
C7B	1.0076 (5)	0.18272 (16)	0.30150 (18)	0.0659 (7)	
H7B1	1.0912	0.2066	0.3563	0.099*	
H7B2	1.0858	0.1542	0.2637	0.099*	
H7B3	0.8940	0.1443	0.3022	0.099*	
C8B	0.7685 (4)	0.21984 (14)	0.19451 (15)	0.0530 (6)	
C8A	0.7970 (3)	0.76385 (13)	0.30896 (13)	0.0435 (5)	
C9A	0.8586 (4)	0.71431 (17)	0.23993 (16)	0.0621 (6)	
H9A	0.9834	0.6973	0.2465	0.075*	
C9B	0.7846 (6)	0.1514 (2)	0.1291 (2)	0.0855 (10)	
H9B	0.8940	0.1244	0.1346	0.103*	
C10A	0.7363 (5)	0.69025 (18)	0.16185 (17)	0.0704 (8)	
H10A	0.7798	0.6576	0.1161	0.085*	
C11A	0.5527 (4)	0.71394 (18)	0.15148 (16)	0.0634 (7)	
C12A	0.4859 (4)	0.76212 (17)	0.21818 (16)	0.0623 (6)	
H12A	0.3599	0.7781	0.2108	0.075*	
C13A	0.6080 (4)	0.78648 (15)	0.29646 (15)	0.0523 (5)	
H13A	0.5626	0.8188	0.3418	0.063*	
C14A	0.2528 (5)	0.6466 (2)	0.04872 (17)	0.0773 (9)	
C15A	0.1621 (6)	0.6282 (2)	−0.04174 (18)	0.0909 (11)	
H15A	0.2016	0.6777	−0.0559	0.109*	
C17A	0.2475 (9)	0.5587 (3)	−0.0944 (3)	0.1381 (18)	
H17D	0.1983	0.5081	−0.0860	0.207*	
H17E	0.3930	0.5707	−0.0789	0.207*	
H17F	0.2059	0.5528	−0.1519	0.207*	
C16A	−0.0658 (7)	0.6106 (3)	−0.0561 (2)	0.1227 (16)	
H16D	−0.1225	0.6019	−0.1132	0.184*	
H16E	−0.1123	0.6567	−0.0209	0.184*	
H16F	−0.1081	0.5618	−0.0432	0.184*	
N1B	0.8656 (3)	0.31381 (11)	0.34072 (11)	0.0465 (4)	
N1A	0.8489 (3)	0.72603 (10)	0.43526 (11)	0.0431 (4)	
N2A	0.7133 (3)	0.75104 (10)	0.48764 (11)	0.0432 (4)	
N2B	0.9297 (3)	0.39715 (11)	0.35052 (11)	0.0442 (4)	
N3B	1.1558 (3)	0.47934 (11)	0.31041 (11)	0.0463 (4)	
H3B	1.0984	0.5209	0.3305	0.056*	
N3A	0.6051 (3)	0.86705 (11)	0.56172 (11)	0.0462 (4)	
H3A	0.5258	0.8359	0.5784	0.055*	
O1B	0.6499 (3)	0.22122 (11)	0.36685 (13)	0.0733 (6)	
O1A	0.9864 (3)	0.62179 (10)	0.36345 (12)	0.0633 (5)	
O2A	0.7210 (4)	0.99870 (11)	0.57378 (15)	0.0877 (7)	
O2B	1.4220 (3)	0.43814 (12)	0.25612 (14)	0.0745 (5)	
O3A	0.4407 (4)	0.69246 (15)	0.07026 (11)	0.0851 (7)	
O4A	0.1729 (4)	0.62528 (18)	0.09728 (14)	0.1077 (9)	
S1A	0.88298 (9)	0.88522 (3)	0.46752 (4)	0.04905 (17)	
S1B	1.15307 (10)	0.31302 (4)	0.25781 (4)	0.05540 (19)	
C14B	0.1788 (9)	0.0980 (5)	−0.0412 (4)	0.1041 (19)	0.723 (5)
C15B	0.0485 (11)	0.0684 (5)	−0.1256 (5)	0.097 (2)	0.723 (5)
H15B	−0.0377	0.0159	−0.1331	0.117*	0.723 (5)
C16B	−0.0847 (12)	0.1334 (5)	−0.1207 (5)	0.143 (3)	0.723 (5)
H16A	−0.1765	0.1199	−0.1732	0.214*	0.723 (5)
H16B	−0.1609	0.1350	−0.0781	0.214*	0.723 (5)
H16C	−0.0015	0.1866	−0.1077	0.214*	0.723 (5)
C17B	0.1577 (13)	0.0551 (5)	−0.1955 (4)	0.115 (2)	0.723 (5)
H17A	0.0609	0.0361	−0.2473	0.173*	0.723 (5)
H17B	0.2408	0.1062	−0.1901	0.173*	0.723 (5)
H17C	0.2417	0.0143	−0.1943	0.173*	0.723 (5)
O3B	0.3507 (7)	0.1413 (3)	−0.0316 (3)	0.1161 (16)	0.723 (5)
O4B	0.1403 (8)	0.0725 (5)	0.0133 (4)	0.171 (3)	0.723 (5)
C14'	0.262 (2)	0.1068 (12)	−0.0760 (10)	0.104 (4)	0.277 (5)
C15'	0.056 (3)	0.0920 (18)	−0.1333 (12)	0.117 (6)	0.277 (5)
H15'	0.0300	0.1483	−0.1257	0.140*	0.277 (5)
C16'	−0.155 (3)	0.0433 (17)	−0.1396 (14)	0.177 (8)	0.277 (5)
H16G	−0.1870	0.0565	−0.0853	0.266*	0.277 (5)
H16H	−0.2556	0.0582	−0.1755	0.266*	0.277 (5)
H16I	−0.1551	−0.0150	−0.1619	0.266*	0.277 (5)
C17'	0.054 (4)	0.0580 (17)	−0.2240 (12)	0.138 (8)	0.277 (5)
H17G	−0.0814	0.0508	−0.2550	0.208*	0.277 (5)
H17H	0.1433	0.0958	−0.2388	0.208*	0.277 (5)
H17I	0.0985	0.0055	−0.2365	0.208*	0.277 (5)
O3B'	0.275 (2)	0.1204 (10)	0.0005 (7)	0.138 (4)	0.277 (5)
O4B'	0.418 (2)	0.1056 (11)	−0.1017 (8)	0.163 (6)	0.277 (5)
O1	−0.348 (3)	−0.0897 (8)	−0.1929 (9)	0.240 (5)*	0.5
H1A	−0.36 (3)	−0.138 (5)	−0.231 (7)	0.288*	0.5
H1B	−0.33 (3)	−0.100 (10)	−0.146 (5)	0.288*	0.5

### 4-(5-Acetamido-3-acetyl-2-methyl-2,3-dihydro-1,3,4-thiadiazol-2-yl)phenyl 2-methylpropanoate 0.25-hydrate (II) Atomic displacement parameters (Å^2^)

**Table:**

	*U*^11^	*U*^22^	*U*^33^	*U*^12^	*U*^13^	*U*^23^
C10B	0.116 (3)	0.092 (3)	0.081 (2)	0.028 (2)	0.005 (2)	−0.0110 (19)
C11B	0.090 (3)	0.110 (3)	0.073 (2)	0.010 (2)	−0.0138 (18)	0.001 (2)
C12B	0.0668 (19)	0.102 (2)	0.0749 (19)	0.0302 (17)	0.0075 (15)	0.0218 (18)
C13B	0.0642 (16)	0.0689 (17)	0.0586 (15)	0.0225 (13)	0.0118 (12)	0.0135 (13)
C1B	0.0747 (17)	0.0555 (15)	0.0730 (17)	0.0097 (13)	0.0389 (14)	0.0239 (13)
C1A	0.0748 (16)	0.0351 (11)	0.0678 (15)	0.0116 (11)	0.0221 (13)	0.0220 (11)
C2A	0.0535 (13)	0.0343 (11)	0.0538 (12)	0.0069 (9)	0.0090 (11)	0.0157 (9)
C2B	0.0623 (15)	0.0467 (13)	0.0574 (13)	0.0080 (11)	0.0211 (11)	0.0249 (11)
C3A	0.0437 (11)	0.0347 (10)	0.0442 (11)	0.0033 (8)	0.0052 (9)	0.0153 (8)
C3B	0.0472 (12)	0.0401 (11)	0.0413 (10)	0.0118 (9)	0.0097 (9)	0.0157 (9)
C4A	0.0701 (16)	0.0366 (12)	0.0609 (14)	0.0078 (11)	0.0166 (12)	0.0142 (10)
C4B	0.0469 (13)	0.0553 (14)	0.0533 (13)	0.0037 (11)	0.0098 (10)	0.0210 (11)
C5A	0.099 (2)	0.0481 (15)	0.092 (2)	0.0249 (15)	0.0407 (18)	0.0182 (14)
C5B	0.0621 (16)	0.0624 (16)	0.0813 (18)	0.0029 (12)	0.0224 (14)	0.0360 (14)
C6B	0.0559 (13)	0.0365 (11)	0.0554 (13)	0.0132 (10)	0.0182 (10)	0.0172 (10)
C6A	0.0427 (11)	0.0345 (10)	0.0570 (12)	0.0038 (8)	0.0137 (10)	0.0180 (9)
C7A	0.0438 (13)	0.0513 (14)	0.0886 (18)	0.0026 (10)	0.0184 (12)	0.0252 (13)
C7B	0.0865 (19)	0.0470 (14)	0.0730 (17)	0.0270 (13)	0.0168 (14)	0.0268 (12)
C8B	0.0578 (14)	0.0434 (12)	0.0559 (13)	0.0124 (10)	0.0138 (11)	0.0119 (10)
C8A	0.0469 (12)	0.0365 (10)	0.0525 (12)	0.0031 (9)	0.0166 (10)	0.0207 (9)
C9A	0.0616 (15)	0.0651 (16)	0.0653 (16)	0.0164 (12)	0.0255 (13)	0.0219 (13)
C9B	0.093 (2)	0.0699 (19)	0.076 (2)	0.0291 (17)	0.0075 (17)	0.0004 (16)
C10A	0.086 (2)	0.0720 (18)	0.0532 (15)	0.0108 (15)	0.0282 (14)	0.0155 (13)
C11A	0.0727 (18)	0.0674 (16)	0.0514 (14)	−0.0018 (13)	0.0130 (12)	0.0269 (12)
C12A	0.0606 (15)	0.0692 (17)	0.0592 (15)	0.0112 (13)	0.0083 (12)	0.0267 (13)
C13A	0.0548 (14)	0.0513 (13)	0.0532 (13)	0.0116 (11)	0.0147 (11)	0.0186 (10)
C14A	0.095 (2)	0.081 (2)	0.0536 (15)	−0.0028 (17)	0.0060 (15)	0.0302 (15)
C15A	0.117 (3)	0.092 (2)	0.0532 (16)	−0.005 (2)	0.0021 (17)	0.0273 (16)
C17A	0.174 (5)	0.154 (5)	0.076 (3)	0.041 (4)	0.028 (3)	0.019 (3)
C16A	0.130 (4)	0.154 (4)	0.071 (2)	0.014 (3)	−0.008 (2)	0.039 (2)
N1B	0.0592 (11)	0.0358 (9)	0.0488 (10)	0.0088 (8)	0.0192 (9)	0.0160 (8)
N1A	0.0471 (10)	0.0328 (9)	0.0525 (10)	0.0058 (7)	0.0130 (8)	0.0180 (8)
N2A	0.0491 (10)	0.0340 (9)	0.0493 (10)	0.0057 (7)	0.0127 (8)	0.0173 (8)
N2B	0.0555 (11)	0.0366 (9)	0.0461 (9)	0.0097 (8)	0.0189 (8)	0.0171 (8)
N3B	0.0512 (11)	0.0400 (10)	0.0536 (10)	0.0098 (8)	0.0201 (8)	0.0188 (8)
N3A	0.0525 (11)	0.0343 (9)	0.0537 (10)	0.0056 (8)	0.0151 (8)	0.0164 (8)
O1B	0.0928 (14)	0.0464 (10)	0.0925 (14)	0.0058 (9)	0.0455 (11)	0.0293 (9)
O1A	0.0766 (12)	0.0427 (9)	0.0826 (12)	0.0184 (8)	0.0391 (10)	0.0242 (8)
O2A	0.1157 (17)	0.0349 (9)	0.1144 (17)	0.0019 (10)	0.0567 (14)	0.0157 (10)
O2B	0.0606 (11)	0.0644 (12)	0.1115 (16)	0.0192 (9)	0.0414 (11)	0.0334 (11)
O3A	0.0955 (16)	0.1057 (17)	0.0484 (10)	−0.0107 (13)	0.0080 (10)	0.0317 (11)
O4A	0.1061 (19)	0.139 (2)	0.0681 (14)	−0.0272 (16)	−0.0003 (13)	0.0480 (15)
S1A	0.0540 (3)	0.0317 (3)	0.0599 (3)	−0.0010 (2)	0.0144 (3)	0.0152 (2)
S1B	0.0551 (4)	0.0462 (3)	0.0695 (4)	0.0163 (3)	0.0258 (3)	0.0170 (3)
C14B	0.076 (3)	0.140 (5)	0.077 (3)	0.007 (3)	0.012 (3)	0.016 (3)
C15B	0.087 (4)	0.096 (5)	0.092 (4)	−0.003 (3)	−0.015 (3)	0.031 (3)
C16B	0.119 (5)	0.144 (6)	0.183 (7)	0.049 (5)	0.010 (5)	0.080 (5)
C17B	0.143 (7)	0.097 (4)	0.082 (4)	−0.004 (5)	0.015 (4)	0.010 (3)
O3B	0.096 (3)	0.168 (4)	0.060 (2)	−0.020 (3)	−0.009 (2)	0.033 (2)
O4B	0.118 (4)	0.243 (7)	0.130 (4)	−0.024 (4)	0.014 (3)	0.059 (4)
C14'	0.104 (7)	0.121 (7)	0.085 (6)	−0.012 (6)	−0.005 (6)	0.055 (6)
C15'	0.093 (8)	0.127 (9)	0.098 (8)	0.016 (8)	−0.002 (7)	0.006 (8)
C16'	0.101 (11)	0.231 (17)	0.143 (13)	0.007 (12)	0.008 (10)	0.001 (13)
C17'	0.135 (15)	0.140 (13)	0.107 (13)	−0.017 (12)	−0.009 (11)	0.027 (11)
O3B'	0.114 (8)	0.175 (8)	0.086 (6)	−0.052 (7)	−0.019 (6)	0.036 (6)
O4B'	0.122 (9)	0.242 (14)	0.130 (10)	0.053 (9)	0.005 (8)	0.071 (9)

### 4-(5-Acetamido-3-acetyl-2-methyl-2,3-dihydro-1,3,4-thiadiazol-2-yl)phenyl 2-methylpropanoate 0.25-hydrate (II) Geometric parameters (Å, º)

**Table:**

C10B—C11B	1.375 (5)	C8A—C13A	1.387 (3)
C10B—C9B	1.381 (5)	C8A—C9A	1.391 (3)
C10B—H10B	0.9300	C9A—C10A	1.379 (4)
C11B—C12B	1.370 (5)	C9A—H9A	0.9300
C11B—O3B	1.430 (5)	C9B—H9B	0.9300
C11B—O3B'	1.476 (11)	C10A—C11A	1.357 (4)
C12B—C13B	1.374 (4)	C10A—H10A	0.9300
C12B—H12B	0.9300	C11A—C12A	1.373 (4)
C13B—C8B	1.382 (4)	C11A—O3A	1.401 (3)
C13B—H13B	0.9300	C12A—C13A	1.381 (3)
C1B—C2B	1.491 (3)	C12A—H12A	0.9300
C1B—H1B1	0.9600	C13A—H13A	0.9300
C1B—H1B2	0.9600	C14A—O4A	1.195 (4)
C1B—H1B3	0.9600	C14A—O3A	1.348 (4)
C1A—C2A	1.498 (3)	C14A—C15A	1.507 (4)
C1A—H1A1	0.9600	C15A—C17A	1.501 (6)
C1A—H1A2	0.9600	C15A—C16A	1.505 (6)
C1A—H1A3	0.9600	C15A—H15A	0.9800
C2A—O1A	1.228 (3)	C17A—H17D	0.9600
C2A—N1A	1.354 (3)	C17A—H17E	0.9600
C2B—O1B	1.228 (3)	C17A—H17F	0.9600
C2B—N1B	1.353 (3)	C16A—H16D	0.9600
C3A—N2A	1.281 (3)	C16A—H16E	0.9600
C3A—N3A	1.368 (3)	C16A—H16F	0.9600
C3A—S1A	1.745 (2)	N1B—N2B	1.402 (2)
C3B—N2B	1.278 (3)	N1A—N2A	1.401 (2)
C3B—N3B	1.374 (3)	N3B—H3B	0.8600
C3B—S1B	1.745 (2)	N3A—H3A	0.8600
C4A—O2A	1.206 (3)	C14B—O4B	1.209 (8)
C4A—N3A	1.373 (3)	C14B—O3B	1.266 (7)
C4A—C5A	1.487 (4)	C14B—C15B	1.482 (8)
C4B—O2B	1.212 (3)	C15B—C17B	1.487 (9)
C4B—N3B	1.372 (3)	C15B—C16B	1.511 (9)
C4B—C5B	1.495 (4)	C15B—H15B	0.9800
C5A—H5A1	0.9600	C16B—H16A	0.9600
C5A—H5A2	0.9600	C16B—H16B	0.9600
C5A—H5A3	0.9600	C16B—H16C	0.9600
C5B—H5B1	0.9600	C17B—H17A	0.9600
C5B—H5B2	0.9600	C17B—H17B	0.9600
C5B—H5B3	0.9600	C17B—H17C	0.9600
C6B—N1B	1.487 (3)	C14'—O4B'	1.212 (15)
C6B—C8B	1.514 (3)	C14'—O3B'	1.256 (14)
C6B—C7B	1.527 (3)	C14'—C15'	1.522 (16)
C6B—S1B	1.846 (2)	C15'—C17'	1.491 (15)
C6A—N1A	1.497 (3)	C15'—C16'	1.544 (16)
C6A—C7A	1.524 (3)	C15'—H15'	0.9800
C6A—C8A	1.529 (3)	C16'—H16G	0.9600
C6A—S1A	1.850 (2)	C16'—H16H	0.9600
C7A—H7A1	0.9600	C16'—H16I	0.9600
C7A—H7A2	0.9600	C17'—H17G	0.9600
C7A—H7A3	0.9600	C17'—H17H	0.9600
C7B—H7B1	0.9600	C17'—H17I	0.9600
C7B—H7B2	0.9600	O1—H1A	0.87 (2)
C7B—H7B3	0.9600	O1—H1B	0.88 (2)
C8B—C9B	1.386 (4)		
			
C11B—C10B—C9B	118.8 (3)	C9A—C10A—H10A	119.9
C11B—C10B—H10B	120.6	C10A—C11A—C12A	120.8 (2)
C9B—C10B—H10B	120.6	C10A—C11A—O3A	117.9 (3)
C12B—C11B—C10B	121.3 (3)	C12A—C11A—O3A	121.2 (3)
C12B—C11B—O3B	119.4 (4)	C11A—C12A—C13A	119.1 (3)
C10B—C11B—O3B	117.8 (4)	C11A—C12A—H12A	120.4
C12B—C11B—O3B'	110.3 (7)	C13A—C12A—H12A	120.4
C10B—C11B—O3B'	122.6 (8)	C12A—C13A—C8A	121.5 (2)
C11B—C12B—C13B	118.9 (3)	C12A—C13A—H13A	119.3
C11B—C12B—H12B	120.5	C8A—C13A—H13A	119.3
C13B—C12B—H12B	120.5	O4A—C14A—O3A	122.5 (3)
C12B—C13B—C8B	121.5 (3)	O4A—C14A—C15A	126.2 (3)
C12B—C13B—H13B	119.2	O3A—C14A—C15A	111.3 (3)
C8B—C13B—H13B	119.2	C17A—C15A—C16A	112.9 (4)
C2B—C1B—H1B1	109.5	C17A—C15A—C14A	109.7 (3)
C2B—C1B—H1B2	109.5	C16A—C15A—C14A	110.0 (3)
H1B1—C1B—H1B2	109.5	C17A—C15A—H15A	108.0
C2B—C1B—H1B3	109.5	C16A—C15A—H15A	108.0
H1B1—C1B—H1B3	109.5	C14A—C15A—H15A	108.0
H1B2—C1B—H1B3	109.5	C15A—C17A—H17D	109.5
C2A—C1A—H1A1	109.5	C15A—C17A—H17E	109.5
C2A—C1A—H1A2	109.5	H17D—C17A—H17E	109.5
H1A1—C1A—H1A2	109.5	C15A—C17A—H17F	109.5
C2A—C1A—H1A3	109.5	H17D—C17A—H17F	109.5
H1A1—C1A—H1A3	109.5	H17E—C17A—H17F	109.5
H1A2—C1A—H1A3	109.5	C15A—C16A—H16D	109.5
O1A—C2A—N1A	120.2 (2)	C15A—C16A—H16E	109.5
O1A—C2A—C1A	122.3 (2)	H16D—C16A—H16E	109.5
N1A—C2A—C1A	117.5 (2)	C15A—C16A—H16F	109.5
O1B—C2B—N1B	120.3 (2)	H16D—C16A—H16F	109.5
O1B—C2B—C1B	122.2 (2)	H16E—C16A—H16F	109.5
N1B—C2B—C1B	117.5 (2)	C2B—N1B—N2B	119.31 (18)
N2A—C3A—N3A	119.36 (19)	C2B—N1B—C6B	122.96 (18)
N2A—C3A—S1A	118.46 (17)	N2B—N1B—C6B	116.47 (16)
N3A—C3A—S1A	122.18 (15)	C2A—N1A—N2A	119.05 (17)
N2B—C3B—N3B	120.21 (19)	C2A—N1A—C6A	123.88 (18)
N2B—C3B—S1B	118.48 (16)	N2A—N1A—C6A	116.36 (16)
N3B—C3B—S1B	121.29 (16)	C3A—N2A—N1A	110.01 (17)
O2A—C4A—N3A	121.3 (2)	C3B—N2B—N1B	109.86 (17)
O2A—C4A—C5A	123.4 (2)	C4B—N3B—C3B	123.7 (2)
N3A—C4A—C5A	115.3 (2)	C4B—N3B—H3B	118.1
O2B—C4B—N3B	121.1 (2)	C3B—N3B—H3B	118.1
O2B—C4B—C5B	123.8 (2)	C3A—N3A—C4A	124.8 (2)
N3B—C4B—C5B	115.2 (2)	C3A—N3A—H3A	117.6
C4A—C5A—H5A1	109.5	C4A—N3A—H3A	117.6
C4A—C5A—H5A2	109.5	C14A—O3A—C11A	119.9 (2)
H5A1—C5A—H5A2	109.5	C3A—S1A—C6A	89.58 (10)
C4A—C5A—H5A3	109.5	C3B—S1B—C6B	89.29 (10)
H5A1—C5A—H5A3	109.5	O4B—C14B—O3B	119.7 (6)
H5A2—C5A—H5A3	109.5	O4B—C14B—C15B	122.5 (7)
C4B—C5B—H5B1	109.5	O3B—C14B—C15B	116.8 (6)
C4B—C5B—H5B2	109.5	C14B—C15B—C17B	115.7 (6)
H5B1—C5B—H5B2	109.5	C14B—C15B—C16B	102.7 (6)
C4B—C5B—H5B3	109.5	C17B—C15B—C16B	113.7 (7)
H5B1—C5B—H5B3	109.5	C14B—C15B—H15B	108.1
H5B2—C5B—H5B3	109.5	C17B—C15B—H15B	108.1
N1B—C6B—C8B	111.11 (18)	C16B—C15B—H15B	108.1
N1B—C6B—C7B	112.30 (19)	C15B—C16B—H16A	109.5
C8B—C6B—C7B	114.0 (2)	C15B—C16B—H16B	109.5
N1B—C6B—S1B	101.72 (14)	H16A—C16B—H16B	109.5
C8B—C6B—S1B	109.87 (16)	C15B—C16B—H16C	109.5
C7B—C6B—S1B	107.00 (18)	H16A—C16B—H16C	109.5
N1A—C6A—C7A	112.66 (18)	H16B—C16B—H16C	109.5
N1A—C6A—C8A	108.50 (16)	C15B—C17B—H17A	109.5
C7A—C6A—C8A	114.9 (2)	C15B—C17B—H17B	109.5
N1A—C6A—S1A	101.53 (14)	H17A—C17B—H17B	109.5
C7A—C6A—S1A	106.51 (15)	C15B—C17B—H17C	109.5
C8A—C6A—S1A	111.94 (15)	H17A—C17B—H17C	109.5
C6A—C7A—H7A1	109.5	H17B—C17B—H17C	109.5
C6A—C7A—H7A2	109.5	C14B—O3B—C11B	115.8 (5)
H7A1—C7A—H7A2	109.5	O4B'—C14'—O3B'	117.3 (15)
C6A—C7A—H7A3	109.5	O4B'—C14'—C15'	122.0 (15)
H7A1—C7A—H7A3	109.5	O3B'—C14'—C15'	120.7 (16)
H7A2—C7A—H7A3	109.5	C17'—C15'—C14'	115.4 (17)
C6B—C7B—H7B1	109.5	C17'—C15'—C16'	95.1 (17)
C6B—C7B—H7B2	109.5	C14'—C15'—C16'	134 (2)
H7B1—C7B—H7B2	109.5	C17'—C15'—H15'	102.9
C6B—C7B—H7B3	109.5	C14'—C15'—H15'	102.9
H7B1—C7B—H7B3	109.5	C16'—C15'—H15'	102.9
H7B2—C7B—H7B3	109.5	C15'—C16'—H16G	109.5
C13B—C8B—C9B	118.0 (3)	C15'—C16'—H16H	109.5
C13B—C8B—C6B	121.6 (2)	H16G—C16'—H16H	109.5
C9B—C8B—C6B	120.0 (2)	C15'—C16'—H16I	109.5
C13A—C8A—C9A	117.7 (2)	H16G—C16'—H16I	109.5
C13A—C8A—C6A	121.2 (2)	H16H—C16'—H16I	109.5
C9A—C8A—C6A	120.9 (2)	C15'—C17'—H17G	109.5
C10A—C9A—C8A	120.6 (3)	C15'—C17'—H17H	109.5
C10A—C9A—H9A	119.7	H17G—C17'—H17H	109.5
C8A—C9A—H9A	119.7	C15'—C17'—H17I	109.5
C10B—C9B—C8B	121.1 (3)	H17G—C17'—H17I	109.5
C10B—C9B—H9B	119.5	H17H—C17'—H17I	109.5
C8B—C9B—H9B	119.5	C14'—O3B'—C11B	111.7 (12)
C11A—C10A—C9A	120.3 (3)	H1A—O1—H1B	105 (3)
C11A—C10A—H10A	119.9		
			
C9B—C10B—C11B—C12B	4.3 (7)	C7A—C6A—N1A—C2A	−54.8 (3)
C9B—C10B—C11B—O3B	170.4 (4)	C8A—C6A—N1A—C2A	73.6 (2)
C9B—C10B—C11B—O3B'	−146.4 (7)	S1A—C6A—N1A—C2A	−168.36 (17)
C10B—C11B—C12B—C13B	−3.3 (7)	C7A—C6A—N1A—N2A	134.9 (2)
O3B—C11B—C12B—C13B	−169.1 (4)	C8A—C6A—N1A—N2A	−96.7 (2)
O3B'—C11B—C12B—C13B	150.6 (7)	S1A—C6A—N1A—N2A	21.4 (2)
C11B—C12B—C13B—C8B	−2.4 (5)	N3A—C3A—N2A—N1A	−177.87 (17)
C12B—C13B—C8B—C9B	6.7 (5)	S1A—C3A—N2A—N1A	1.7 (2)
C12B—C13B—C8B—C6B	179.5 (3)	C2A—N1A—N2A—C3A	172.95 (19)
N1B—C6B—C8B—C13B	18.3 (3)	C6A—N1A—N2A—C3A	−16.3 (2)
C7B—C6B—C8B—C13B	146.5 (3)	N3B—C3B—N2B—N1B	178.38 (18)
S1B—C6B—C8B—C13B	−93.5 (3)	S1B—C3B—N2B—N1B	−0.3 (2)
N1B—C6B—C8B—C9B	−169.0 (3)	C2B—N1B—N2B—C3B	177.4 (2)
C7B—C6B—C8B—C9B	−40.9 (3)	C6B—N1B—N2B—C3B	−15.1 (3)
S1B—C6B—C8B—C9B	79.2 (3)	O2B—C4B—N3B—C3B	−5.9 (4)
N1A—C6A—C8A—C13A	80.1 (2)	C5B—C4B—N3B—C3B	173.3 (2)
C7A—C6A—C8A—C13A	−152.8 (2)	N2B—C3B—N3B—C4B	171.3 (2)
S1A—C6A—C8A—C13A	−31.1 (2)	S1B—C3B—N3B—C4B	−10.1 (3)
N1A—C6A—C8A—C9A	−94.9 (2)	N2A—C3A—N3A—C4A	177.7 (2)
C7A—C6A—C8A—C9A	32.2 (3)	S1A—C3A—N3A—C4A	−1.9 (3)
S1A—C6A—C8A—C9A	153.89 (19)	O2A—C4A—N3A—C3A	−4.3 (4)
C13A—C8A—C9A—C10A	1.2 (4)	C5A—C4A—N3A—C3A	174.5 (2)
C6A—C8A—C9A—C10A	176.4 (2)	O4A—C14A—O3A—C11A	2.6 (5)
C11B—C10B—C9B—C8B	0.3 (6)	C15A—C14A—O3A—C11A	−178.1 (3)
C13B—C8B—C9B—C10B	−5.6 (5)	C10A—C11A—O3A—C14A	121.9 (3)
C6B—C8B—C9B—C10B	−178.5 (3)	C12A—C11A—O3A—C14A	−62.3 (4)
C8A—C9A—C10A—C11A	−0.7 (4)	N2A—C3A—S1A—C6A	9.51 (18)
C9A—C10A—C11A—C12A	0.1 (4)	N3A—C3A—S1A—C6A	−170.93 (18)
C9A—C10A—C11A—O3A	175.8 (2)	N1A—C6A—S1A—C3A	−15.74 (14)
C10A—C11A—C12A—C13A	0.1 (4)	C7A—C6A—S1A—C3A	−133.82 (17)
O3A—C11A—C12A—C13A	−175.5 (2)	C8A—C6A—S1A—C3A	99.82 (15)
C11A—C12A—C13A—C8A	0.5 (4)	N2B—C3B—S1B—C6B	11.17 (18)
C9A—C8A—C13A—C12A	−1.1 (3)	N3B—C3B—S1B—C6B	−167.47 (18)
C6A—C8A—C13A—C12A	−176.2 (2)	N1B—C6B—S1B—C3B	−16.53 (14)
O4A—C14A—C15A—C17A	−101.7 (5)	C8B—C6B—S1B—C3B	101.25 (16)
O3A—C14A—C15A—C17A	79.1 (4)	C7B—C6B—S1B—C3B	−134.49 (17)
O4A—C14A—C15A—C16A	23.1 (6)	O4B—C14B—C15B—C17B	−139.6 (8)
O3A—C14A—C15A—C16A	−156.1 (3)	O3B—C14B—C15B—C17B	29.2 (10)
O1B—C2B—N1B—N2B	173.7 (2)	O4B—C14B—C15B—C16B	95.9 (9)
C1B—C2B—N1B—N2B	−6.5 (3)	O3B—C14B—C15B—C16B	−95.3 (8)
O1B—C2B—N1B—C6B	7.0 (4)	O4B—C14B—O3B—C11B	−6.2 (10)
C1B—C2B—N1B—C6B	−173.2 (2)	C15B—C14B—O3B—C11B	−175.3 (6)
C8B—C6B—N1B—C2B	71.5 (3)	C12B—C11B—O3B—C14B	−88.4 (7)
C7B—C6B—N1B—C2B	−57.5 (3)	C10B—C11B—O3B—C14B	105.3 (6)
S1B—C6B—N1B—C2B	−171.57 (18)	O4B'—C14'—C15'—C17'	13 (3)
C8B—C6B—N1B—N2B	−95.5 (2)	O3B'—C14'—C15'—C17'	−167 (2)
C7B—C6B—N1B—N2B	135.4 (2)	O4B'—C14'—C15'—C16'	139 (3)
S1B—C6B—N1B—N2B	21.4 (2)	O3B'—C14'—C15'—C16'	−41 (4)
O1A—C2A—N1A—N2A	175.0 (2)	O4B'—C14'—O3B'—C11B	22 (3)
C1A—C2A—N1A—N2A	−5.1 (3)	C15'—C14'—O3B'—C11B	−158.1 (18)
O1A—C2A—N1A—C6A	5.0 (3)	C12B—C11B—O3B'—C14'	125.5 (14)
C1A—C2A—N1A—C6A	−175.1 (2)	C10B—C11B—O3B'—C14'	−81.1 (17)

### 4-(5-Acetamido-3-acetyl-2-methyl-2,3-dihydro-1,3,4-thiadiazol-2-yl)phenyl 2-methylpropanoate 0.25-hydrate (II) Hydrogen-bond geometry (Å, º)

*Cg*2 and *Cg*4 are the centroids of the C8*B*–C13*B* and C8*A*–C13*A* rings, respectively.

**Table:**

*D*—H···*A*	*D*—H	H···*A*	*D*···*A*	*D*—H···*A*
O1—H1*B*···O4*B*^i^	0.88 (11)	2.32 (10)	3.111 (16)	149 (12)
N3*A*—H3*A*···O1*B*^ii^	0.86	1.99	2.842 (3)	171
N3*B*—H3*B*···O1*A*	0.86	1.94	2.792 (3)	171
C15*B*—H15*B*···O1	0.98	2.46	3.368 (19)	154
C7*B*—H7*B*2···O1^iii^	0.96	2.49	3.434 (19)	168
C15*A*—H15*A*···*Cg*2^iv^	0.98	2.99	3.959 (4)	168
C17*B*—H17*B*···*Cg*4^iv^	0.96	2.98	3.864 (9)	153
C17′—H17*H*···*Cg*4^iv^	0.96	2.93	3.81 (3)	154

Symmetry codes: (i) −*x*, −*y*, −*z*; (ii) −*x*+1, −*y*+1, −*z*+1; (iii) −*x*+1, −*y*, −*z*; (iv) −*x*+1, −*y*+1, −*z*.

### 4-(5-Acetamido-3-acetyl-2-methyl-2,3-dihydro-1,3,4-thiadiazol-2-yl)phenyl propionate (III) Crystal data

**Table:**

C_16_H_19_N_3_O_4_S	*Z* = 4
*M**_r_* = 349.40	*F*(000) = 736
Triclinic, *P*1	*D*_x_ = 1.313 Mg m^−^^3^
*a* = 11.4150 (3) Å	Mo *K*α radiation, λ = 0.71073 Å
*b* = 12.4021 (3) Å	Cell parameters from 7257 reflections
*c* = 13.2305 (3) Å	θ = 1.8–26.9°
α = 71.982 (1)°	µ = 0.21 mm^−^^1^
β = 89.829 (1)°	*T* = 293 K
γ = 83.114 (1)°	Block, colourless
*V* = 1767.18 (8) Å^3^	0.25 × 0.24 × 0.20 mm

### 4-(5-Acetamido-3-acetyl-2-methyl-2,3-dihydro-1,3,4-thiadiazol-2-yl)phenyl propionate (III) Data collection

**Table:**

Bruker Kappa APEXII CCD diffractometer	5869 reflections with *I* > 2σ(*I*)
ω and φ scans	*R*_int_ = 0.022
Absorption correction: multi-scan (*SADABS*; Bruker, 2008)	θ_max_ = 26.4°, θ_min_ = 1.6°
*T*_min_ = 0.756, *T*_max_ = 0.824	*h* = −14→14
26933 measured reflections	*k* = −15→15
7257 independent reflections	*l* = −16→16

### 4-(5-Acetamido-3-acetyl-2-methyl-2,3-dihydro-1,3,4-thiadiazol-2-yl)phenyl propionate (III) Refinement

**Table:**

Refinement on *F*^2^	Primary atom site location: structure-invariant direct methods
Least-squares matrix: full	Secondary atom site location: difference Fourier map
*R*[*F*^2^ > 2σ(*F*^2^)] = 0.037	Hydrogen site location: inferred from neighbouring sites
*wR*(*F*^2^) = 0.106	H-atom parameters constrained
*S* = 1.03	*w* = 1/[σ^2^(*F*_o_^2^) + (0.0486*P*)^2^ + 0.4775*P*] where *P* = (*F*_o_^2^ + 2*F*_c_^2^)/3
7257 reflections	(Δ/σ)_max_ = 0.012
451 parameters	Δρ_max_ = 0.24 e Å^−^^3^
0 restraints	Δρ_min_ = −0.33 e Å^−^^3^

### 4-(5-Acetamido-3-acetyl-2-methyl-2,3-dihydro-1,3,4-thiadiazol-2-yl)phenyl propionate (III) Special details

**Table:**

Geometry. All esds (except the esd in the dihedral angle between two l.s. planes) are estimated using the full covariance matrix. The cell esds are taken into account individually in the estimation of esds in distances, angles and torsion angles; correlations between esds in cell parameters are only used when they are defined by crystal symmetry. An approximate (isotropic) treatment of cell esds is used for estimating esds involving l.s. planes.

### 4-(5-Acetamido-3-acetyl-2-methyl-2,3-dihydro-1,3,4-thiadiazol-2-yl)phenyl propionate (III) Fractional atomic coordinates and isotropic or equivalent isotropic displacement parameters (Å^2^)

**Table:**

	*x*	*y*	*z*	*U*_iso_*/*U*_eq_	Occ. (<1)
N1A	0.57800 (12)	1.34448 (11)	0.74760 (12)	0.0525 (3)	
H1A	0.620681	1.370276	0.786145	0.063*	
O1B	0.30213 (13)	0.50078 (14)	0.84275 (14)	0.0854 (5)	
O2A	0.73259 (12)	0.86411 (11)	0.91134 (11)	0.0707 (4)	
C5A	0.51016 (19)	1.54430 (15)	0.65971 (18)	0.0696 (5)	
H5A1	0.557477	1.576433	0.600418	0.104*	
H5A2	0.545055	1.550089	0.723459	0.104*	
H5A3	0.432011	1.585300	0.647905	0.104*	
C5B	0.41647 (18)	0.3577 (2)	0.9822 (2)	0.0847 (7)	
H5B1	0.440346	0.286150	0.969805	0.127*	
H5B2	0.402035	0.344392	1.056366	0.127*	
H5B3	0.477912	0.405929	0.961867	0.127*	
C4B	0.30600 (16)	0.41497 (16)	0.91784 (16)	0.0595 (4)	
C4A	0.50385 (16)	1.42199 (14)	0.67108 (15)	0.0545 (4)	
C3B	0.09508 (14)	0.40749 (12)	0.90740 (12)	0.0430 (3)	
C3A	0.58882 (13)	1.22853 (13)	0.76694 (12)	0.0443 (3)	
C2B	−0.20340 (15)	0.38900 (14)	0.93581 (13)	0.0475 (4)	
C2A	0.74122 (16)	0.96458 (16)	0.90086 (14)	0.0557 (4)	
C1A	0.8399 (2)	1.0004 (2)	0.95195 (19)	0.0819 (7)	
H1A1	0.894461	0.934423	0.987754	0.123*	
H1A2	0.808622	1.036605	1.002461	0.123*	
H1A3	0.879963	1.053137	0.898454	0.123*	
C1B	−0.20176 (18)	0.30017 (18)	1.04232 (15)	0.0661 (5)	
H1B1	−0.281171	0.286795	1.061371	0.099*	
H1B2	−0.165756	0.326230	1.094501	0.099*	
H1B3	−0.157297	0.230441	1.039480	0.099*	
C6A	0.56363 (14)	1.02464 (13)	0.77881 (13)	0.0480 (4)	
C6B	−0.08741 (14)	0.49848 (13)	0.78791 (12)	0.0439 (3)	
C7B	−0.15459 (18)	0.61708 (14)	0.77098 (15)	0.0599 (5)	
H7B1	−0.237776	0.613673	0.765099	0.090*	
H7B2	−0.129831	0.669363	0.706902	0.090*	
H7B3	−0.138429	0.642805	0.830298	0.090*	
C7A	0.46982 (17)	0.97282 (17)	0.85428 (16)	0.0631 (5)	
H7A1	0.498929	0.895550	0.894576	0.095*	
H7A2	0.399801	0.973411	0.814005	0.095*	
H7A3	0.451600	1.016824	0.901843	0.095*	
C8A	0.60797 (14)	0.95904 (13)	0.70392 (13)	0.0467 (4)	
C8B	−0.11842 (13)	0.44221 (13)	0.70592 (12)	0.0427 (3)	
C13B	−0.11426 (16)	0.32545 (14)	0.73185 (13)	0.0504 (4)	
H13B	−0.093842	0.280152	0.801309	0.060*	
C9A	0.53636 (16)	0.89412 (15)	0.66959 (16)	0.0593 (4)	
H9A	0.461151	0.886891	0.696048	0.071*	
C12B	−0.13966 (17)	0.27416 (15)	0.65726 (14)	0.0555 (4)	
H12B	−0.136280	0.195077	0.676341	0.067*	
C10A	0.57469 (17)	0.83983 (15)	0.59663 (16)	0.0616 (5)	
H10A	0.525298	0.797237	0.573355	0.074*	
C11B	−0.16969 (15)	0.33979 (16)	0.55560 (14)	0.0545 (4)	
C11A	0.68586 (16)	0.84921 (13)	0.55888 (14)	0.0520 (4)	
C10B	−0.1753 (2)	0.45512 (18)	0.52740 (16)	0.0848 (7)	
H10B	−0.196507	0.499665	0.457871	0.102*	
C12A	0.75876 (17)	0.91397 (17)	0.59031 (16)	0.0606 (4)	
H12A	0.834058	0.920341	0.563862	0.073*	
C13A	0.71888 (16)	0.96928 (16)	0.66141 (15)	0.0588 (4)	
H13A	0.767410	1.014739	0.681596	0.071*	
C9B	−0.1494 (2)	0.50628 (16)	0.60244 (15)	0.0772 (7)	
H9B	−0.152876	0.585410	0.582606	0.093*	
C14B	−0.11985 (18)	0.23156 (17)	0.43963 (15)	0.0599 (4)	
C14A	0.75675 (15)	0.84105 (14)	0.39311 (14)	0.0518 (4)	
C15B	−0.1704 (2)	0.18925 (18)	0.35733 (16)	0.0677 (5)	
H15A	−0.210289	0.253823	0.301650	0.081*	
H15B	−0.228848	0.139518	0.389471	0.081*	
C15A	0.81032 (18)	0.75962 (16)	0.33897 (16)	0.0637 (5)	
H15C	0.759433	0.700728	0.345461	0.076*	
H15D	0.885816	0.722948	0.373824	0.076*	
C16A	0.8284 (2)	0.8183 (2)	0.22260 (18)	0.0888 (7)	
H16A	0.753154	0.849116	0.186584	0.133*	
H16B	0.867850	0.764059	0.191567	0.133*	
H16C	0.875623	0.879079	0.215732	0.133*	
C16B	−0.0785 (2)	0.1249 (2)	0.30835 (19)	0.0837 (7)	
H16D	−0.022136	0.174521	0.273753	0.126*	
H16E	−0.115993	0.098469	0.257153	0.126*	
H16F	−0.038825	0.060581	0.362960	0.126*	
N1B	0.20675 (12)	0.36478 (12)	0.95143 (11)	0.0508 (3)	
H1B	0.214485	0.302221	1.003664	0.061*	
N2B	0.00570 (12)	0.36683 (11)	0.95615 (10)	0.0462 (3)	
N2A	0.66403 (12)	1.16162 (11)	0.83704 (11)	0.0489 (3)	
N3A	0.66139 (12)	1.04853 (11)	0.83936 (11)	0.0495 (3)	
N3B	−0.09860 (11)	0.42081 (11)	0.89767 (10)	0.0440 (3)	
O1A	0.43966 (13)	1.39102 (11)	0.61613 (12)	0.0764 (4)	
O2B	−0.29535 (10)	0.43215 (11)	0.88346 (10)	0.0582 (3)	
O3A	0.72748 (12)	0.78457 (10)	0.49319 (10)	0.0604 (3)	
O3B	−0.20361 (12)	0.29134 (13)	0.47883 (11)	0.0701 (4)	
O4A	0.73852 (12)	0.94268 (10)	0.35619 (10)	0.0626 (3)	
S1B	0.07220 (4)	0.51371 (4)	0.78475 (3)	0.05286 (12)	
S1A	0.50357 (4)	1.16990 (4)	0.69416 (4)	0.05734 (14)	
O4B	−0.0175 (6)	0.231 (2)	0.4588 (16)	0.081 (3)	0.68 (6)
O4B'	−0.030 (3)	0.184 (4)	0.491 (3)	0.081 (7)	0.32 (6)

### 4-(5-Acetamido-3-acetyl-2-methyl-2,3-dihydro-1,3,4-thiadiazol-2-yl)phenyl propionate (III) Atomic displacement parameters (Å^2^)

**Table:**

	*U*^11^	*U*^22^	*U*^33^	*U*^12^	*U*^13^	*U*^23^
N1A	0.0511 (8)	0.0467 (7)	0.0612 (8)	−0.0057 (6)	−0.0134 (7)	−0.0190 (6)
O1B	0.0618 (9)	0.0804 (10)	0.0964 (11)	−0.0195 (7)	0.0056 (8)	0.0021 (9)
O2A	0.0677 (8)	0.0523 (7)	0.0770 (9)	0.0030 (6)	−0.0193 (7)	−0.0022 (6)
C5A	0.0723 (13)	0.0466 (10)	0.0855 (14)	−0.0058 (9)	−0.0078 (11)	−0.0147 (9)
C5B	0.0452 (11)	0.0925 (16)	0.1061 (18)	−0.0056 (10)	−0.0050 (11)	−0.0169 (14)
C4B	0.0485 (10)	0.0601 (11)	0.0697 (12)	−0.0076 (8)	0.0031 (9)	−0.0199 (9)
C4A	0.0515 (10)	0.0487 (9)	0.0608 (10)	−0.0051 (7)	−0.0054 (8)	−0.0140 (8)
C3B	0.0461 (9)	0.0391 (7)	0.0438 (8)	−0.0035 (6)	−0.0026 (7)	−0.0134 (6)
C3A	0.0400 (8)	0.0479 (8)	0.0456 (8)	−0.0048 (6)	−0.0033 (7)	−0.0156 (7)
C2B	0.0488 (9)	0.0551 (9)	0.0449 (8)	−0.0095 (7)	0.0018 (7)	−0.0236 (7)
C2A	0.0506 (10)	0.0570 (10)	0.0493 (9)	−0.0006 (8)	−0.0095 (8)	−0.0041 (8)
C1A	0.0676 (13)	0.0804 (14)	0.0819 (14)	−0.0057 (11)	−0.0356 (11)	−0.0035 (11)
C1B	0.0649 (12)	0.0773 (13)	0.0548 (10)	−0.0212 (10)	0.0086 (9)	−0.0141 (9)
C6A	0.0451 (9)	0.0445 (8)	0.0513 (9)	−0.0009 (7)	−0.0111 (7)	−0.0119 (7)
C6B	0.0473 (9)	0.0409 (8)	0.0405 (8)	−0.0045 (6)	−0.0030 (7)	−0.0085 (6)
C7B	0.0708 (12)	0.0451 (9)	0.0607 (11)	0.0037 (8)	−0.0053 (9)	−0.0161 (8)
C7A	0.0535 (10)	0.0682 (11)	0.0631 (11)	−0.0028 (9)	−0.0014 (9)	−0.0158 (9)
C8A	0.0465 (9)	0.0371 (7)	0.0527 (9)	−0.0011 (6)	−0.0079 (7)	−0.0100 (7)
C8B	0.0425 (8)	0.0427 (8)	0.0402 (8)	−0.0020 (6)	−0.0041 (6)	−0.0105 (6)
C13B	0.0623 (10)	0.0449 (8)	0.0412 (8)	−0.0051 (7)	0.0019 (7)	−0.0102 (7)
C9A	0.0492 (10)	0.0549 (10)	0.0800 (13)	−0.0093 (8)	0.0005 (9)	−0.0288 (9)
C12B	0.0661 (11)	0.0473 (9)	0.0572 (10)	−0.0131 (8)	0.0092 (8)	−0.0198 (8)
C10A	0.0603 (11)	0.0510 (10)	0.0809 (13)	−0.0124 (8)	−0.0052 (10)	−0.0292 (9)
C11B	0.0507 (9)	0.0642 (10)	0.0547 (10)	−0.0053 (8)	−0.0077 (8)	−0.0281 (8)
C11A	0.0603 (10)	0.0378 (8)	0.0555 (9)	0.0034 (7)	−0.0078 (8)	−0.0147 (7)
C10B	0.137 (2)	0.0612 (12)	0.0497 (11)	0.0032 (12)	−0.0350 (12)	−0.0127 (9)
C12A	0.0523 (10)	0.0676 (11)	0.0669 (11)	−0.0107 (8)	0.0035 (9)	−0.0269 (9)
C13A	0.0569 (10)	0.0612 (10)	0.0673 (11)	−0.0190 (8)	0.0005 (9)	−0.0286 (9)
C9B	0.130 (2)	0.0439 (9)	0.0515 (10)	−0.0020 (11)	−0.0264 (11)	−0.0090 (8)
C14B	0.0666 (12)	0.0665 (11)	0.0524 (10)	−0.0142 (10)	−0.0012 (9)	−0.0247 (9)
C14A	0.0477 (9)	0.0474 (9)	0.0609 (10)	0.0000 (7)	−0.0114 (8)	−0.0201 (8)
C15B	0.0867 (14)	0.0679 (12)	0.0568 (11)	−0.0235 (10)	−0.0046 (10)	−0.0265 (9)
C15A	0.0602 (11)	0.0600 (11)	0.0729 (12)	0.0093 (9)	−0.0081 (9)	−0.0296 (9)
C16A	0.0977 (18)	0.0957 (17)	0.0725 (14)	0.0254 (14)	−0.0056 (12)	−0.0390 (13)
C16B	0.1012 (18)	0.0925 (16)	0.0759 (14)	−0.0311 (14)	0.0079 (13)	−0.0458 (13)
N1B	0.0448 (7)	0.0487 (7)	0.0536 (8)	−0.0030 (6)	−0.0044 (6)	−0.0091 (6)
N2B	0.0455 (7)	0.0470 (7)	0.0415 (7)	−0.0025 (6)	−0.0039 (6)	−0.0083 (6)
N2A	0.0479 (8)	0.0493 (7)	0.0483 (7)	−0.0059 (6)	−0.0089 (6)	−0.0136 (6)
N3A	0.0488 (8)	0.0472 (7)	0.0497 (7)	0.0006 (6)	−0.0136 (6)	−0.0133 (6)
N3B	0.0425 (7)	0.0482 (7)	0.0390 (6)	−0.0044 (5)	−0.0033 (5)	−0.0108 (5)
O1A	0.0831 (10)	0.0533 (7)	0.0862 (10)	−0.0041 (7)	−0.0394 (8)	−0.0135 (7)
O2B	0.0444 (6)	0.0747 (8)	0.0602 (7)	−0.0063 (6)	−0.0045 (6)	−0.0281 (6)
O3A	0.0765 (8)	0.0419 (6)	0.0612 (7)	0.0042 (6)	−0.0027 (6)	−0.0177 (5)
O3B	0.0641 (8)	0.0866 (9)	0.0729 (9)	−0.0045 (7)	−0.0138 (7)	−0.0458 (8)
O4A	0.0758 (9)	0.0461 (7)	0.0658 (8)	−0.0045 (6)	−0.0038 (6)	−0.0182 (6)
S1B	0.0526 (2)	0.0518 (2)	0.0476 (2)	−0.01414 (18)	−0.00393 (18)	−0.00294 (18)
S1A	0.0614 (3)	0.0439 (2)	0.0653 (3)	0.00323 (18)	−0.0282 (2)	−0.01838 (19)
O4B	0.0605 (18)	0.116 (7)	0.089 (5)	−0.015 (3)	0.001 (2)	−0.064 (5)
O4B'	0.075 (7)	0.105 (13)	0.078 (8)	0.010 (8)	−0.011 (6)	−0.058 (9)

### 4-(5-Acetamido-3-acetyl-2-methyl-2,3-dihydro-1,3,4-thiadiazol-2-yl)phenyl propionate (III) Geometric parameters (Å, º)

**Table:**

N1A—C4A	1.369 (2)	C8A—C9A	1.382 (2)
N1A—C3A	1.371 (2)	C8A—C13A	1.388 (2)
N1A—H1A	0.8600	C8B—C9B	1.376 (2)
O1B—C4B	1.207 (2)	C8B—C13B	1.376 (2)
O2A—C2A	1.226 (2)	C13B—C12B	1.377 (2)
C5A—C4A	1.488 (2)	C13B—H13B	0.9300
C5A—H5A1	0.9600	C9A—C10A	1.380 (3)
C5A—H5A2	0.9600	C9A—H9A	0.9300
C5A—H5A3	0.9600	C12B—C11B	1.359 (2)
C5B—C4B	1.496 (3)	C12B—H12B	0.9300
C5B—H5B1	0.9600	C10A—C11A	1.367 (3)
C5B—H5B2	0.9600	C10A—H10A	0.9300
C5B—H5B3	0.9600	C11B—C10B	1.355 (3)
C4B—N1B	1.365 (2)	C11B—O3B	1.406 (2)
C4A—O1A	1.207 (2)	C11A—C12A	1.371 (3)
C3B—N2B	1.277 (2)	C11A—O3A	1.400 (2)
C3B—N1B	1.377 (2)	C10B—C9B	1.383 (3)
C3B—S1B	1.7420 (15)	C10B—H10B	0.9300
C3A—N2A	1.2821 (19)	C12A—C13A	1.373 (3)
C3A—S1A	1.7353 (16)	C12A—H12A	0.9300
C2B—O2B	1.228 (2)	C13A—H13A	0.9300
C2B—N3B	1.352 (2)	C9B—H9B	0.9300
C2B—C1B	1.495 (2)	C14B—O4B	1.196 (7)
C2A—N3A	1.352 (2)	C14B—O4B'	1.218 (14)
C2A—C1A	1.494 (3)	C14B—O3B	1.341 (2)
C1A—H1A1	0.9600	C14B—C15B	1.491 (3)
C1A—H1A2	0.9600	C14A—O4A	1.195 (2)
C1A—H1A3	0.9600	C14A—O3A	1.355 (2)
C1B—H1B1	0.9600	C14A—C15A	1.484 (2)
C1B—H1B2	0.9600	C15B—C16B	1.508 (3)
C1B—H1B3	0.9600	C15B—H15A	0.9700
C6A—N3A	1.485 (2)	C15B—H15B	0.9700
C6A—C8A	1.515 (2)	C15A—C16A	1.511 (3)
C6A—C7A	1.523 (3)	C15A—H15C	0.9700
C6A—S1A	1.8520 (16)	C15A—H15D	0.9700
C6B—N3B	1.4887 (19)	C16A—H16A	0.9600
C6B—C8B	1.523 (2)	C16A—H16B	0.9600
C6B—C7B	1.526 (2)	C16A—H16C	0.9600
C6B—S1B	1.8531 (16)	C16B—H16D	0.9600
C7B—H7B1	0.9600	C16B—H16E	0.9600
C7B—H7B2	0.9600	C16B—H16F	0.9600
C7B—H7B3	0.9600	N1B—H1B	0.8600
C7A—H7A1	0.9600	N2B—N3B	1.3993 (17)
C7A—H7A2	0.9600	N2A—N3A	1.3973 (19)
C7A—H7A3	0.9600		
			
C4A—N1A—C3A	124.42 (14)	C8B—C13B—H13B	119.3
C4A—N1A—H1A	117.8	C12B—C13B—H13B	119.3
C3A—N1A—H1A	117.8	C10A—C9A—C8A	121.04 (17)
C4A—C5A—H5A1	109.5	C10A—C9A—H9A	119.5
C4A—C5A—H5A2	109.5	C8A—C9A—H9A	119.5
H5A1—C5A—H5A2	109.5	C11B—C12B—C13B	119.62 (16)
C4A—C5A—H5A3	109.5	C11B—C12B—H12B	120.2
H5A1—C5A—H5A3	109.5	C13B—C12B—H12B	120.2
H5A2—C5A—H5A3	109.5	C11A—C10A—C9A	119.44 (17)
C4B—C5B—H5B1	109.5	C11A—C10A—H10A	120.3
C4B—C5B—H5B2	109.5	C9A—C10A—H10A	120.3
H5B1—C5B—H5B2	109.5	C10B—C11B—C12B	120.48 (16)
C4B—C5B—H5B3	109.5	C10B—C11B—O3B	118.06 (16)
H5B1—C5B—H5B3	109.5	C12B—C11B—O3B	121.31 (16)
H5B2—C5B—H5B3	109.5	C10A—C11A—C12A	121.05 (17)
O1B—C4B—N1B	121.23 (17)	C10A—C11A—O3A	118.62 (16)
O1B—C4B—C5B	123.57 (18)	C12A—C11A—O3A	120.17 (17)
N1B—C4B—C5B	115.18 (17)	C11B—C10B—C9B	119.73 (17)
O1A—C4A—N1A	120.96 (16)	C11B—C10B—H10B	120.1
O1A—C4A—C5A	123.29 (17)	C9B—C10B—H10B	120.1
N1A—C4A—C5A	115.74 (16)	C11A—C12A—C13A	118.98 (18)
N2B—C3B—N1B	119.51 (14)	C11A—C12A—H12A	120.5
N2B—C3B—S1B	118.75 (12)	C13A—C12A—H12A	120.5
N1B—C3B—S1B	121.73 (12)	C12A—C13A—C8A	121.63 (16)
N2A—C3A—N1A	120.77 (14)	C12A—C13A—H13A	119.2
N2A—C3A—S1A	118.74 (12)	C8A—C13A—H13A	119.2
N1A—C3A—S1A	120.46 (11)	C8B—C9B—C10B	121.19 (17)
O2B—C2B—N3B	120.27 (15)	C8B—C9B—H9B	119.4
O2B—C2B—C1B	122.29 (16)	C10B—C9B—H9B	119.4
N3B—C2B—C1B	117.44 (15)	O4B—C14B—O3B	120.9 (4)
O2A—C2A—N3A	120.37 (16)	O4B'—C14B—O3B	121.3 (8)
O2A—C2A—C1A	122.52 (16)	O4B—C14B—C15B	126.8 (4)
N3A—C2A—C1A	117.10 (17)	O4B'—C14B—C15B	122.2 (11)
C2A—C1A—H1A1	109.5	O3B—C14B—C15B	111.26 (17)
C2A—C1A—H1A2	109.5	O4A—C14A—O3A	122.58 (16)
H1A1—C1A—H1A2	109.5	O4A—C14A—C15A	126.69 (18)
C2A—C1A—H1A3	109.5	O3A—C14A—C15A	110.72 (15)
H1A1—C1A—H1A3	109.5	C14B—C15B—C16B	113.05 (19)
H1A2—C1A—H1A3	109.5	C14B—C15B—H15A	109.0
C2B—C1B—H1B1	109.5	C16B—C15B—H15A	109.0
C2B—C1B—H1B2	109.5	C14B—C15B—H15B	109.0
H1B1—C1B—H1B2	109.5	C16B—C15B—H15B	109.0
C2B—C1B—H1B3	109.5	H15A—C15B—H15B	107.8
H1B1—C1B—H1B3	109.5	C14A—C15A—C16A	111.95 (17)
H1B2—C1B—H1B3	109.5	C14A—C15A—H15C	109.2
N3A—C6A—C8A	112.33 (13)	C16A—C15A—H15C	109.2
N3A—C6A—C7A	110.08 (14)	C14A—C15A—H15D	109.2
C8A—C6A—C7A	115.48 (14)	C16A—C15A—H15D	109.2
N3A—C6A—S1A	102.03 (10)	H15C—C15A—H15D	107.9
C8A—C6A—S1A	106.50 (11)	C15A—C16A—H16A	109.5
C7A—C6A—S1A	109.47 (12)	C15A—C16A—H16B	109.5
N3B—C6B—C8B	110.60 (12)	H16A—C16A—H16B	109.5
N3B—C6B—C7B	112.08 (13)	C15A—C16A—H16C	109.5
C8B—C6B—C7B	114.21 (13)	H16A—C16A—H16C	109.5
N3B—C6B—S1B	102.21 (10)	H16B—C16A—H16C	109.5
C8B—C6B—S1B	109.71 (11)	C15B—C16B—H16D	109.5
C7B—C6B—S1B	107.27 (12)	C15B—C16B—H16E	109.5
C6B—C7B—H7B1	109.5	H16D—C16B—H16E	109.5
C6B—C7B—H7B2	109.5	C15B—C16B—H16F	109.5
H7B1—C7B—H7B2	109.5	H16D—C16B—H16F	109.5
C6B—C7B—H7B3	109.5	H16E—C16B—H16F	109.5
H7B1—C7B—H7B3	109.5	C4B—N1B—C3B	124.47 (15)
H7B2—C7B—H7B3	109.5	C4B—N1B—H1B	117.8
C6A—C7A—H7A1	109.5	C3B—N1B—H1B	117.8
C6A—C7A—H7A2	109.5	C3B—N2B—N3B	110.39 (12)
H7A1—C7A—H7A2	109.5	C3A—N2A—N3A	110.07 (13)
C6A—C7A—H7A3	109.5	C2A—N3A—N2A	120.02 (14)
H7A1—C7A—H7A3	109.5	C2A—N3A—C6A	122.34 (14)
H7A2—C7A—H7A3	109.5	N2A—N3A—C6A	117.49 (12)
C9A—C8A—C13A	117.81 (16)	C2B—N3B—N2B	119.44 (13)
C9A—C8A—C6A	121.21 (15)	C2B—N3B—C6B	122.35 (13)
C13A—C8A—C6A	120.81 (15)	N2B—N3B—C6B	117.53 (12)
C9B—C8B—C13B	117.51 (15)	C14A—O3A—C11A	118.09 (13)
C9B—C8B—C6B	121.12 (14)	C14B—O3B—C11B	118.32 (15)
C13B—C8B—C6B	121.36 (13)	C3B—S1B—C6B	89.96 (7)
C8B—C13B—C12B	121.48 (15)	C3A—S1A—C6A	89.90 (7)
			
C3A—N1A—C4A—O1A	1.8 (3)	S1B—C3B—N2B—N3B	−0.89 (18)
C3A—N1A—C4A—C5A	−177.04 (17)	N1A—C3A—N2A—N3A	−177.85 (14)
C4A—N1A—C3A—N2A	176.96 (17)	S1A—C3A—N2A—N3A	0.39 (19)
C4A—N1A—C3A—S1A	−1.2 (2)	O2A—C2A—N3A—N2A	172.94 (16)
N3A—C6A—C8A—C9A	156.77 (15)	C1A—C2A—N3A—N2A	−8.5 (3)
C7A—C6A—C8A—C9A	29.4 (2)	O2A—C2A—N3A—C6A	−2.5 (3)
S1A—C6A—C8A—C9A	−92.34 (16)	C1A—C2A—N3A—C6A	176.07 (18)
N3A—C6A—C8A—C13A	−28.1 (2)	C3A—N2A—N3A—C2A	173.99 (16)
C7A—C6A—C8A—C13A	−155.50 (16)	C3A—N2A—N3A—C6A	−10.4 (2)
S1A—C6A—C8A—C13A	82.75 (16)	C8A—C6A—N3A—C2A	−56.7 (2)
N3B—C6B—C8B—C9B	160.12 (18)	C7A—C6A—N3A—C2A	73.5 (2)
C7B—C6B—C8B—C9B	32.6 (2)	S1A—C6A—N3A—C2A	−170.36 (14)
S1B—C6B—C8B—C9B	−87.87 (19)	C8A—C6A—N3A—N2A	127.76 (14)
N3B—C6B—C8B—C13B	−21.2 (2)	C7A—C6A—N3A—N2A	−102.04 (16)
C7B—C6B—C8B—C13B	−148.76 (16)	S1A—C6A—N3A—N2A	14.10 (17)
S1B—C6B—C8B—C13B	90.79 (16)	O2B—C2B—N3B—N2B	−176.98 (14)
C9B—C8B—C13B—C12B	0.2 (3)	C1B—C2B—N3B—N2B	2.7 (2)
C6B—C8B—C13B—C12B	−178.50 (16)	O2B—C2B—N3B—C6B	−6.7 (2)
C13A—C8A—C9A—C10A	1.0 (3)	C1B—C2B—N3B—C6B	172.99 (15)
C6A—C8A—C9A—C10A	176.22 (16)	C3B—N2B—N3B—C2B	179.54 (14)
C8B—C13B—C12B—C11B	−0.1 (3)	C3B—N2B—N3B—C6B	8.79 (19)
C8A—C9A—C10A—C11A	0.9 (3)	C8B—C6B—N3B—C2B	−65.22 (18)
C13B—C12B—C11B—C10B	−0.3 (3)	C7B—C6B—N3B—C2B	63.48 (19)
C13B—C12B—C11B—O3B	−175.85 (17)	S1B—C6B—N3B—C2B	178.04 (12)
C9A—C10A—C11A—C12A	−1.6 (3)	C8B—C6B—N3B—N2B	105.25 (15)
C9A—C10A—C11A—O3A	173.75 (16)	C7B—C6B—N3B—N2B	−126.05 (15)
C12B—C11B—C10B—C9B	0.5 (4)	S1B—C6B—N3B—N2B	−11.50 (15)
O3B—C11B—C10B—C9B	176.2 (2)	O4A—C14A—O3A—C11A	−6.9 (2)
C10A—C11A—C12A—C13A	0.5 (3)	C15A—C14A—O3A—C11A	174.20 (15)
O3A—C11A—C12A—C13A	−174.87 (16)	C10A—C11A—O3A—C14A	119.16 (18)
C11A—C12A—C13A—C8A	1.5 (3)	C12A—C11A—O3A—C14A	−65.4 (2)
C9A—C8A—C13A—C12A	−2.2 (3)	O4B—C14B—O3B—C11B	−9.4 (16)
C6A—C8A—C13A—C12A	−177.47 (16)	O4B'—C14B—O3B—C11B	27 (3)
C13B—C8B—C9B—C10B	0.0 (3)	C15B—C14B—O3B—C11B	−178.40 (16)
C6B—C8B—C9B—C10B	178.7 (2)	C10B—C11B—O3B—C14B	110.6 (2)
C11B—C10B—C9B—C8B	−0.4 (4)	C12B—C11B—O3B—C14B	−73.7 (2)
O4B—C14B—C15B—C16B	9.9 (17)	N2B—C3B—S1B—C6B	−5.09 (13)
O4B'—C14B—C15B—C16B	−27 (3)	N1B—C3B—S1B—C6B	173.34 (14)
O3B—C14B—C15B—C16B	178.04 (18)	N3B—C6B—S1B—C3B	8.44 (10)
O4A—C14A—C15A—C16A	−6.8 (3)	C8B—C6B—S1B—C3B	−108.95 (11)
O3A—C14A—C15A—C16A	172.10 (18)	C7B—C6B—S1B—C3B	126.48 (12)
O1B—C4B—N1B—C3B	−2.5 (3)	N2A—C3A—S1A—C6A	6.85 (15)
C5B—C4B—N1B—C3B	175.98 (18)	N1A—C3A—S1A—C6A	−174.91 (14)
N2B—C3B—N1B—C4B	−167.01 (16)	N3A—C6A—S1A—C3A	−10.64 (11)
S1B—C3B—N1B—C4B	14.6 (2)	C8A—C6A—S1A—C3A	−128.55 (11)
N1B—C3B—N2B—N3B	−179.36 (13)	C7A—C6A—S1A—C3A	105.95 (13)

### 4-(5-Acetamido-3-acetyl-2-methyl-2,3-dihydro-1,3,4-thiadiazol-2-yl)phenyl propionate (III) Hydrogen-bond geometry (Å, º)

**Table:**

*D*—H···*A*	*D*—H	H···*A*	*D*···*A*	*D*—H···*A*
N1*A*—H1*A*···O2*B*^i^	0.86	1.99	2.8469 (19)	174
N1*B*—H1*B*···O2*A*^ii^	0.86	2.04	2.860 (2)	160
C9*B*—H9*B*···O3*A*^iii^	0.93	2.60	3.426 (2)	148

Symmetry codes: (i) *x*+1, *y*+1, *z*; (ii) −*x*+1, −*y*+1, −*z*+2; (iii) *x*−1, *y*, *z*.

### 4-(5-Acetamido-3-acetyl-2-methyl-2,3-dihydro-1,3,4-thiadiazol-2-yl)phenyl cinnamate chloroform hemisolvate (IV) Crystal data

**Table:**

C_22_H_21_N_3_O_4_S·0.5CHCl_3_	*Z* = 4
*M**_r_* = 483.16	*F*(000) = 1004
Triclinic, *P*1	*D*_x_ = 1.350 Mg m^−^^3^
*a* = 10.7427 (1) Å	Mo *K*α radiation, λ = 0.71073 Å
*b* = 11.0828 (2) Å	Cell parameters from 8335 reflections
*c* = 20.8969 (3) Å	θ = 1.8–26.9°
α = 93.186 (1)°	µ = 0.34 mm^−^^1^
β = 103.945 (4)°	*T* = 293 K
γ = 98.489 (2)°	Block, colourless
*V* = 2377.39 (7) Å^3^	0.30 × 0.25 × 0.20 mm

### 4-(5-Acetamido-3-acetyl-2-methyl-2,3-dihydro-1,3,4-thiadiazol-2-yl)phenyl cinnamate chloroform hemisolvate (IV) Data collection

**Table:**

Bruker Kappa APEXII CCD diffractometer	6495 reflections with *I* > 2σ(*I*)
ω and φ scans	*R*_int_ = 0.027
Absorption correction: multi-scan (*SADABS*; Bruker, 2008)	θ_max_ = 25.0°, θ_min_ = 1.9°
*T*_min_ = 0.741, *T*_max_ = 0.856	*h* = −12→12
31719 measured reflections	*k* = −13→13
8335 independent reflections	*l* = −24→24

### 4-(5-Acetamido-3-acetyl-2-methyl-2,3-dihydro-1,3,4-thiadiazol-2-yl)phenyl cinnamate chloroform hemisolvate (IV) Refinement

**Table:**

Refinement on *F*^2^	Primary atom site location: structure-invariant direct methods
Least-squares matrix: full	Secondary atom site location: difference Fourier map
*R*[*F*^2^ > 2σ(*F*^2^)] = 0.058	Hydrogen site location: inferred from neighbouring sites
*wR*(*F*^2^) = 0.195	H-atom parameters constrained
*S* = 1.09	*w* = 1/[σ^2^(*F*_o_^2^) + (0.1202*P*)^2^ + 0.801*P*] where *P* = (*F*_o_^2^ + 2*F*_c_^2^)/3
8335 reflections	(Δ/σ)_max_ = 0.001
583 parameters	Δρ_max_ = 0.54 e Å^−^^3^
0 restraints	Δρ_min_ = −0.60 e Å^−^^3^

### 4-(5-Acetamido-3-acetyl-2-methyl-2,3-dihydro-1,3,4-thiadiazol-2-yl)phenyl cinnamate chloroform hemisolvate (IV) Special details

**Table:**

Geometry. All esds (except the esd in the dihedral angle between two l.s. planes) are estimated using the full covariance matrix. The cell esds are taken into account individually in the estimation of esds in distances, angles and torsion angles; correlations between esds in cell parameters are only used when they are defined by crystal symmetry. An approximate (isotropic) treatment of cell esds is used for estimating esds involving l.s. planes.

### 4-(5-Acetamido-3-acetyl-2-methyl-2,3-dihydro-1,3,4-thiadiazol-2-yl)phenyl cinnamate chloroform hemisolvate (IV) Fractional atomic coordinates and isotropic or equivalent isotropic displacement parameters (Å^2^)

**Table:**

	*x*	*y*	*z*	*U*_iso_*/*U*_eq_	
C1A	0.7194 (4)	0.1544 (3)	0.06419 (17)	0.0762 (10)	
H1A1	0.679416	0.205916	0.032712	0.114*	
H1A2	0.656947	0.117324	0.086277	0.114*	
H1A3	0.749989	0.091555	0.041580	0.114*	
C1B	0.6139 (3)	0.7996 (3)	0.05275 (15)	0.0639 (8)	
H1B1	0.695480	0.854537	0.064442	0.096*	
H1B2	0.544321	0.845480	0.051555	0.096*	
H1B3	0.602589	0.757019	0.009951	0.096*	
C2A	0.8309 (3)	0.2293 (3)	0.11376 (14)	0.0533 (7)	
C2B	0.6126 (3)	0.7094 (2)	0.10287 (13)	0.0484 (6)	
C3A	0.9511 (2)	0.0017 (2)	0.20325 (12)	0.0428 (5)	
C3B	0.3223 (2)	0.5173 (2)	0.03235 (12)	0.0427 (5)	
C4A	1.0210 (3)	−0.1768 (3)	0.25439 (14)	0.0513 (6)	
C4B	0.1169 (3)	0.3955 (3)	−0.02802 (15)	0.0583 (7)	
C5A	0.9753 (3)	−0.3054 (3)	0.26544 (17)	0.0680 (8)	
H5A1	1.045685	−0.351101	0.269622	0.102*	
H5A2	0.905121	−0.341904	0.228557	0.102*	
H5A3	0.945710	−0.306228	0.305263	0.102*	
C5B	0.0036 (3)	0.3916 (4)	−0.08641 (18)	0.0799 (10)	
H5B1	−0.051581	0.313263	−0.092202	0.120*	
H5B2	0.034438	0.403920	−0.125414	0.120*	
H5B3	−0.044694	0.455046	−0.079113	0.120*	
C6A	1.0106 (2)	0.2332 (2)	0.21533 (13)	0.0465 (6)	
C6B	0.4970 (2)	0.5235 (2)	0.13959 (12)	0.0432 (5)	
C7A	1.1137 (3)	0.3185 (3)	0.19223 (17)	0.0632 (8)	
H7A1	1.128809	0.280281	0.153057	0.095*	
H7A2	1.193202	0.334094	0.226558	0.095*	
H7A3	1.083562	0.394438	0.182604	0.095*	
C7B	0.6069 (3)	0.4474 (3)	0.14865 (15)	0.0574 (7)	
H7B1	0.611552	0.413704	0.106132	0.086*	
H7B2	0.590135	0.382120	0.175304	0.086*	
H7B3	0.687998	0.498827	0.170196	0.086*	
C8A	0.9573 (2)	0.2918 (2)	0.26755 (13)	0.0439 (6)	
C8B	0.4791 (2)	0.5838 (2)	0.20332 (12)	0.0430 (5)	
C9A	0.8302 (3)	0.2560 (3)	0.27116 (15)	0.0579 (7)	
H9A	0.774920	0.197301	0.239339	0.069*	
C9B	0.5678 (3)	0.5896 (3)	0.26339 (14)	0.0604 (7)	
H9B	0.642341	0.554829	0.266019	0.072*	
C10A	0.7844 (3)	0.3056 (3)	0.32095 (16)	0.0636 (8)	
H10A	0.698835	0.280633	0.322681	0.076*	
C10B	0.5487 (3)	0.6453 (3)	0.31927 (15)	0.0672 (8)	
H10B	0.609121	0.646886	0.359678	0.081*	
C11A	0.8654 (3)	0.3919 (3)	0.36804 (14)	0.0549 (7)	
C11B	0.4411 (3)	0.6985 (3)	0.31586 (14)	0.0543 (7)	
C12A	0.9920 (3)	0.4299 (3)	0.36570 (15)	0.0574 (7)	
H12A	1.046685	0.488670	0.397663	0.069*	
C12B	0.3529 (3)	0.6951 (4)	0.25795 (16)	0.0733 (9)	
H12B	0.279542	0.731560	0.255771	0.088*	
C13A	1.0363 (3)	0.3802 (3)	0.31574 (14)	0.0530 (7)	
H13A	1.121732	0.406325	0.314039	0.064*	
C13B	0.3712 (3)	0.6372 (4)	0.20168 (15)	0.0702 (9)	
H13B	0.309114	0.634359	0.161743	0.084*	
C14A	0.8162 (3)	0.5525 (3)	0.43336 (15)	0.0570 (7)	
C14B	0.3303 (3)	0.7229 (3)	0.39933 (14)	0.0551 (7)	
C15A	0.7524 (3)	0.5734 (3)	0.48581 (14)	0.0606 (7)	
H15A	0.722788	0.507454	0.506914	0.073*	
C15B	0.3246 (3)	0.8066 (3)	0.45431 (14)	0.0590 (7)	
H15B	0.395847	0.867181	0.473069	0.071*	
C16A	0.7357 (3)	0.6851 (3)	0.50409 (14)	0.0582 (7)	
H16A	0.767123	0.747609	0.481341	0.070*	
C16B	0.2218 (3)	0.7990 (3)	0.47811 (14)	0.0563 (7)	
H16B	0.154112	0.735313	0.458827	0.068*	
C17A	0.6744 (3)	0.7222 (3)	0.55536 (14)	0.0571 (7)	
C17B	0.2007 (3)	0.8783 (3)	0.53116 (13)	0.0527 (6)	
C18A	0.6129 (4)	0.6396 (3)	0.5895 (2)	0.0805 (10)	
H18A	0.607888	0.556028	0.579260	0.097*	
C18B	0.0791 (3)	0.8648 (4)	0.54331 (18)	0.0796 (10)	
H18B	0.012376	0.805620	0.517842	0.096*	
C19A	0.5588 (4)	0.6798 (4)	0.6385 (2)	0.0973 (13)	
H19A	0.516647	0.623096	0.660692	0.117*	
C19B	0.0559 (4)	0.9382 (5)	0.5928 (2)	0.1002 (14)	
H19B	−0.026287	0.927828	0.600861	0.120*	
C20A	0.5667 (4)	0.8028 (4)	0.6549 (2)	0.0883 (12)	
H20A	0.530013	0.829440	0.687996	0.106*	
C20B	0.1510 (5)	1.0252 (4)	0.6299 (2)	0.0951 (13)	
H20B	0.133699	1.075734	0.662592	0.114*	
C21A	0.6284 (3)	0.8853 (4)	0.62237 (18)	0.0754 (10)	
H21A	0.634720	0.968781	0.633653	0.090*	
C21B	0.2717 (5)	1.0388 (4)	0.61940 (19)	0.0928 (13)	
H21B	0.337669	1.098062	0.645371	0.111*	
C22A	0.6817 (3)	0.8462 (3)	0.57277 (16)	0.0656 (8)	
H22A	0.723142	0.903687	0.550683	0.079*	
C22B	0.2968 (3)	0.9659 (3)	0.57086 (16)	0.0704 (9)	
H22B	0.380255	0.975483	0.564433	0.085*	
C24	1.3645 (7)	0.9979 (5)	0.1756 (3)	0.1254 (18)	
H24	1.320200	0.999896	0.211398	0.150*	
N1A	0.9043 (2)	0.1694 (2)	0.15926 (10)	0.0479 (5)	
N1B	0.5106 (2)	0.61660 (19)	0.09130 (10)	0.0433 (5)	
N2A	0.9315 (2)	−0.1208 (2)	0.21358 (11)	0.0470 (5)	
H2A	0.858413	−0.164752	0.193076	0.056*	
N2B	0.2116 (2)	0.4957 (2)	−0.01916 (11)	0.0502 (5)	
H2B	0.201657	0.548981	−0.047555	0.060*	
N3A	0.8666 (2)	0.04364 (19)	0.16030 (11)	0.0469 (5)	
N3B	0.4087 (2)	0.61029 (19)	0.03472 (10)	0.0445 (5)	
O1A	0.8567 (2)	0.34125 (19)	0.11497 (11)	0.0623 (5)	
O1B	0.70071 (19)	0.7159 (2)	0.15333 (10)	0.0637 (5)	
O2A	1.1291 (2)	−0.1226 (2)	0.28060 (13)	0.0768 (7)	
O2B	0.1249 (3)	0.3165 (2)	0.00948 (13)	0.0913 (8)	
O3A	0.8151 (2)	0.4318 (2)	0.42002 (11)	0.0691 (6)	
O3B	0.4300 (2)	0.7646 (2)	0.37297 (10)	0.0688 (6)	
O4A	0.8612 (3)	0.6288 (2)	0.40394 (13)	0.0848 (8)	
O4B	0.2575 (2)	0.6284 (2)	0.37835 (12)	0.0732 (6)	
Cl1	1.5222 (2)	0.9672 (2)	0.20900 (13)	0.1808 (8)	
Cl2	1.2776 (3)	0.8889 (2)	0.11689 (11)	0.2206 (13)	
Cl3	1.3765 (3)	1.14052 (19)	0.14707 (17)	0.2181 (12)	
S1A	1.08765 (6)	0.10243 (6)	0.24908 (4)	0.0508 (2)	
S1B	0.34553 (7)	0.42085 (6)	0.09567 (3)	0.0545 (2)	

### 4-(5-Acetamido-3-acetyl-2-methyl-2,3-dihydro-1,3,4-thiadiazol-2-yl)phenyl cinnamate chloroform hemisolvate (IV) Atomic displacement parameters (Å^2^)

**Table:**

	*U*^11^	*U*^22^	*U*^33^	*U*^12^	*U*^13^	*U*^23^
C1A	0.082 (2)	0.065 (2)	0.067 (2)	0.0200 (17)	−0.0145 (17)	0.0092 (16)
C1B	0.0673 (18)	0.0550 (17)	0.0610 (17)	−0.0091 (14)	0.0098 (14)	0.0149 (14)
C2A	0.0551 (15)	0.0531 (17)	0.0535 (15)	0.0188 (13)	0.0101 (13)	0.0093 (12)
C2B	0.0463 (14)	0.0460 (14)	0.0510 (15)	0.0035 (11)	0.0110 (12)	0.0059 (11)
C3A	0.0395 (12)	0.0463 (14)	0.0437 (13)	0.0122 (10)	0.0097 (10)	0.0046 (11)
C3B	0.0470 (13)	0.0409 (13)	0.0413 (13)	0.0067 (11)	0.0136 (10)	0.0038 (10)
C4A	0.0485 (15)	0.0518 (15)	0.0544 (15)	0.0179 (12)	0.0084 (12)	0.0055 (12)
C4B	0.0587 (16)	0.0536 (17)	0.0563 (16)	−0.0040 (13)	0.0114 (13)	0.0007 (14)
C5A	0.0688 (19)	0.0624 (19)	0.071 (2)	0.0157 (15)	0.0074 (16)	0.0210 (15)
C5B	0.068 (2)	0.076 (2)	0.075 (2)	−0.0125 (17)	−0.0051 (17)	−0.0012 (18)
C6A	0.0375 (12)	0.0462 (14)	0.0553 (15)	0.0080 (10)	0.0090 (11)	0.0094 (11)
C6B	0.0468 (13)	0.0433 (13)	0.0422 (13)	0.0083 (10)	0.0143 (10)	0.0106 (10)
C7A	0.0517 (16)	0.0670 (19)	0.0725 (19)	0.0017 (14)	0.0215 (14)	0.0158 (15)
C7B	0.0640 (17)	0.0566 (17)	0.0588 (16)	0.0224 (14)	0.0210 (14)	0.0104 (13)
C8A	0.0391 (12)	0.0391 (13)	0.0522 (14)	0.0072 (10)	0.0072 (11)	0.0106 (11)
C8B	0.0404 (12)	0.0481 (14)	0.0416 (13)	0.0088 (10)	0.0104 (10)	0.0097 (10)
C9A	0.0432 (14)	0.0574 (17)	0.0686 (18)	−0.0024 (12)	0.0151 (13)	−0.0080 (14)
C9B	0.0535 (16)	0.079 (2)	0.0504 (16)	0.0248 (14)	0.0077 (13)	0.0047 (14)
C10A	0.0484 (15)	0.0632 (19)	0.080 (2)	0.0017 (13)	0.0239 (15)	−0.0034 (16)
C10B	0.0640 (18)	0.089 (2)	0.0446 (15)	0.0199 (16)	0.0038 (13)	−0.0036 (15)
C11A	0.0644 (17)	0.0493 (15)	0.0547 (16)	0.0140 (13)	0.0188 (13)	0.0063 (12)
C11B	0.0523 (15)	0.0601 (17)	0.0498 (15)	−0.0003 (13)	0.0195 (12)	−0.0050 (13)
C12A	0.0552 (16)	0.0505 (16)	0.0596 (17)	0.0053 (13)	0.0051 (13)	−0.0037 (13)
C12B	0.0629 (18)	0.103 (3)	0.0615 (18)	0.0410 (18)	0.0159 (15)	−0.0015 (18)
C13A	0.0397 (13)	0.0475 (15)	0.0659 (17)	0.0024 (11)	0.0054 (12)	0.0017 (13)
C13B	0.0597 (18)	0.106 (3)	0.0482 (16)	0.0386 (18)	0.0066 (14)	0.0016 (16)
C14A	0.0629 (17)	0.0595 (18)	0.0515 (15)	0.0194 (14)	0.0134 (13)	0.0090 (14)
C14B	0.0583 (16)	0.0581 (17)	0.0510 (15)	0.0077 (14)	0.0195 (13)	0.0038 (13)
C15A	0.0626 (17)	0.070 (2)	0.0518 (16)	0.0162 (15)	0.0148 (14)	0.0086 (14)
C15B	0.0604 (17)	0.0621 (18)	0.0525 (16)	0.0006 (14)	0.0185 (13)	−0.0034 (13)
C16A	0.0579 (16)	0.0639 (18)	0.0537 (16)	0.0156 (14)	0.0118 (13)	0.0082 (13)
C16B	0.0610 (17)	0.0563 (17)	0.0521 (15)	0.0044 (13)	0.0188 (13)	0.0036 (13)
C17A	0.0501 (15)	0.0641 (18)	0.0551 (16)	0.0129 (13)	0.0084 (12)	0.0005 (14)
C17B	0.0589 (16)	0.0563 (16)	0.0472 (14)	0.0114 (13)	0.0196 (12)	0.0082 (12)
C18A	0.087 (2)	0.065 (2)	0.100 (3)	0.0131 (18)	0.046 (2)	−0.0006 (19)
C18B	0.0610 (19)	0.103 (3)	0.076 (2)	0.0076 (18)	0.0262 (17)	−0.005 (2)
C19A	0.104 (3)	0.091 (3)	0.115 (3)	0.009 (2)	0.066 (3)	0.008 (2)
C19B	0.079 (3)	0.144 (4)	0.093 (3)	0.034 (3)	0.046 (2)	0.001 (3)
C20A	0.078 (2)	0.098 (3)	0.093 (3)	0.008 (2)	0.038 (2)	−0.020 (2)
C20B	0.126 (4)	0.099 (3)	0.074 (2)	0.031 (3)	0.047 (3)	−0.009 (2)
C21A	0.0651 (19)	0.074 (2)	0.080 (2)	0.0074 (17)	0.0121 (17)	−0.0206 (18)
C21B	0.116 (3)	0.087 (3)	0.069 (2)	−0.013 (2)	0.035 (2)	−0.017 (2)
C22A	0.0594 (17)	0.069 (2)	0.0665 (19)	0.0123 (15)	0.0124 (15)	0.0028 (15)
C22B	0.0693 (19)	0.078 (2)	0.0634 (19)	−0.0020 (16)	0.0272 (16)	−0.0027 (16)
C24	0.168 (5)	0.093 (3)	0.119 (4)	0.023 (3)	0.043 (4)	0.006 (3)
N1A	0.0458 (11)	0.0486 (12)	0.0466 (12)	0.0108 (9)	0.0037 (9)	0.0088 (9)
N1B	0.0444 (11)	0.0436 (11)	0.0404 (11)	0.0034 (9)	0.0091 (9)	0.0086 (9)
N2A	0.0415 (11)	0.0471 (12)	0.0500 (12)	0.0090 (9)	0.0055 (9)	0.0059 (9)
N2B	0.0505 (12)	0.0498 (13)	0.0457 (12)	0.0005 (10)	0.0067 (10)	0.0097 (10)
N3A	0.0456 (11)	0.0440 (12)	0.0479 (12)	0.0093 (9)	0.0045 (9)	0.0055 (9)
N3B	0.0440 (11)	0.0464 (12)	0.0399 (11)	0.0032 (9)	0.0065 (9)	0.0059 (9)
O1A	0.0632 (12)	0.0530 (12)	0.0688 (13)	0.0158 (9)	0.0061 (10)	0.0190 (10)
O1B	0.0515 (11)	0.0662 (13)	0.0604 (12)	−0.0058 (9)	−0.0027 (9)	0.0112 (10)
O2A	0.0540 (12)	0.0581 (13)	0.1058 (18)	0.0173 (10)	−0.0092 (12)	0.0100 (12)
O2B	0.0909 (18)	0.0728 (16)	0.0852 (17)	−0.0283 (13)	−0.0044 (14)	0.0229 (14)
O3A	0.0904 (16)	0.0576 (13)	0.0693 (13)	0.0136 (11)	0.0388 (12)	0.0051 (10)
O3B	0.0651 (12)	0.0771 (15)	0.0625 (12)	−0.0080 (11)	0.0302 (10)	−0.0173 (11)
O4A	0.121 (2)	0.0660 (15)	0.0900 (17)	0.0323 (14)	0.0583 (16)	0.0181 (13)
O4B	0.0861 (15)	0.0571 (13)	0.0781 (15)	−0.0089 (12)	0.0397 (12)	−0.0078 (11)
Cl1	0.1491 (16)	0.1571 (16)	0.220 (2)	0.0242 (13)	0.0127 (15)	0.0345 (15)
Cl2	0.292 (3)	0.1581 (18)	0.1448 (16)	0.0026 (19)	−0.0530 (18)	0.0216 (13)
Cl3	0.261 (3)	0.1176 (13)	0.342 (3)	0.0661 (16)	0.164 (3)	0.1005 (18)
S1A	0.0372 (3)	0.0504 (4)	0.0610 (4)	0.0115 (3)	0.0027 (3)	0.0053 (3)
S1B	0.0620 (4)	0.0483 (4)	0.0473 (4)	−0.0053 (3)	0.0097 (3)	0.0128 (3)

### 4-(5-Acetamido-3-acetyl-2-methyl-2,3-dihydro-1,3,4-thiadiazol-2-yl)phenyl cinnamate chloroform hemisolvate (IV) Geometric parameters (Å, º)

**Table:**

C1A—C2A	1.487 (4)	C10B—H10B	0.9300
C1A—H1A1	0.9600	C11A—C12A	1.376 (4)
C1A—H1A2	0.9600	C11A—O3A	1.403 (3)
C1A—H1A3	0.9600	C11B—C12B	1.340 (4)
C1B—C2B	1.489 (4)	C11B—O3B	1.404 (3)
C1B—H1B1	0.9600	C12A—C13A	1.369 (4)
C1B—H1B2	0.9600	C12A—H12A	0.9300
C1B—H1B3	0.9600	C12B—C13B	1.377 (4)
C2A—O1A	1.228 (3)	C12B—H12B	0.9300
C2A—N1A	1.353 (3)	C13A—H13A	0.9300
C2B—O1B	1.226 (3)	C13B—H13B	0.9300
C2B—N1B	1.352 (3)	C14A—O4A	1.187 (4)
C3A—N3A	1.275 (3)	C14A—O3A	1.350 (4)
C3A—N2A	1.379 (3)	C14A—C15A	1.451 (4)
C3A—S1A	1.742 (3)	C14B—O4B	1.202 (4)
C3B—N3B	1.271 (3)	C14B—O3B	1.353 (4)
C3B—N2B	1.379 (3)	C14B—C15B	1.455 (4)
C3B—S1B	1.741 (3)	C15A—C16A	1.325 (4)
C4A—O2A	1.209 (3)	C15A—H15A	0.9300
C4A—N2A	1.370 (3)	C15B—C16B	1.310 (4)
C4A—C5A	1.486 (4)	C15B—H15B	0.9300
C4B—O2B	1.206 (4)	C16A—C17A	1.457 (4)
C4B—N2B	1.362 (4)	C16A—H16A	0.9300
C4B—C5B	1.495 (4)	C16B—C17B	1.457 (4)
C5A—H5A1	0.9600	C16B—H16B	0.9300
C5A—H5A2	0.9600	C17A—C18A	1.379 (5)
C5A—H5A3	0.9600	C17A—C22A	1.388 (4)
C5B—H5B1	0.9600	C17B—C22B	1.378 (4)
C5B—H5B2	0.9600	C17B—C18B	1.379 (4)
C5B—H5B3	0.9600	C18A—C19A	1.378 (5)
C6A—N1A	1.484 (3)	C18A—H18A	0.9300
C6A—C8A	1.508 (4)	C18B—C19B	1.373 (5)
C6A—C7A	1.533 (4)	C18B—H18B	0.9300
C6A—S1A	1.863 (3)	C19A—C20A	1.372 (6)
C6B—N1B	1.499 (3)	C19A—H19A	0.9300
C6B—C8B	1.523 (3)	C19B—C20B	1.348 (7)
C6B—C7B	1.531 (4)	C19B—H19B	0.9300
C6B—S1B	1.845 (3)	C20A—C21A	1.358 (6)
C7A—H7A1	0.9600	C20A—H20A	0.9300
C7A—H7A2	0.9600	C20B—C21B	1.355 (6)
C7A—H7A3	0.9600	C20B—H20B	0.9300
C7B—H7B1	0.9600	C21A—C22A	1.378 (5)
C7B—H7B2	0.9600	C21A—H21A	0.9300
C7B—H7B3	0.9600	C21B—C22B	1.364 (5)
C8A—C9A	1.386 (4)	C21B—H21B	0.9300
C8A—C13A	1.388 (4)	C22A—H22A	0.9300
C8B—C13B	1.371 (4)	C22B—H22B	0.9300
C8B—C9B	1.373 (4)	C24—Cl2	1.669 (6)
C9A—C10A	1.375 (4)	C24—Cl3	1.718 (6)
C9A—H9A	0.9300	C24—Cl1	1.761 (7)
C9B—C10B	1.363 (4)	C24—H24	0.9800
C9B—H9B	0.9300	N1A—N3A	1.396 (3)
C10A—C11A	1.370 (4)	N1B—N3B	1.395 (3)
C10A—H10A	0.9300	N2A—H2A	0.8600
C10B—C11B	1.362 (4)	N2B—H2B	0.8600
			
C2A—C1A—H1A1	109.5	C13A—C12A—C11A	119.2 (3)
C2A—C1A—H1A2	109.5	C13A—C12A—H12A	120.4
H1A1—C1A—H1A2	109.5	C11A—C12A—H12A	120.4
C2A—C1A—H1A3	109.5	C11B—C12B—C13B	119.7 (3)
H1A1—C1A—H1A3	109.5	C11B—C12B—H12B	120.1
H1A2—C1A—H1A3	109.5	C13B—C12B—H12B	120.1
C2B—C1B—H1B1	109.5	C12A—C13A—C8A	121.8 (3)
C2B—C1B—H1B2	109.5	C12A—C13A—H13A	119.1
H1B1—C1B—H1B2	109.5	C8A—C13A—H13A	119.1
C2B—C1B—H1B3	109.5	C8B—C13B—C12B	121.4 (3)
H1B1—C1B—H1B3	109.5	C8B—C13B—H13B	119.3
H1B2—C1B—H1B3	109.5	C12B—C13B—H13B	119.3
O1A—C2A—N1A	120.1 (3)	O4A—C14A—O3A	122.9 (3)
O1A—C2A—C1A	122.7 (2)	O4A—C14A—C15A	126.1 (3)
N1A—C2A—C1A	117.2 (3)	O3A—C14A—C15A	111.0 (3)
O1B—C2B—N1B	119.5 (2)	O4B—C14B—O3B	122.5 (3)
O1B—C2B—C1B	122.4 (2)	O4B—C14B—C15B	126.1 (3)
N1B—C2B—C1B	118.1 (2)	O3B—C14B—C15B	111.4 (3)
N3A—C3A—N2A	119.8 (2)	C16A—C15A—C14A	121.0 (3)
N3A—C3A—S1A	118.7 (2)	C16A—C15A—H15A	119.5
N2A—C3A—S1A	121.56 (18)	C14A—C15A—H15A	119.5
N3B—C3B—N2B	119.9 (2)	C16B—C15B—C14B	121.8 (3)
N3B—C3B—S1B	118.86 (19)	C16B—C15B—H15B	119.1
N2B—C3B—S1B	121.26 (19)	C14B—C15B—H15B	119.1
O2A—C4A—N2A	121.2 (3)	C15A—C16A—C17A	128.1 (3)
O2A—C4A—C5A	122.9 (3)	C15A—C16A—H16A	115.9
N2A—C4A—C5A	115.9 (2)	C17A—C16A—H16A	115.9
O2B—C4B—N2B	121.5 (3)	C15B—C16B—C17B	127.8 (3)
O2B—C4B—C5B	123.0 (3)	C15B—C16B—H16B	116.1
N2B—C4B—C5B	115.5 (3)	C17B—C16B—H16B	116.1
C4A—C5A—H5A1	109.5	C18A—C17A—C22A	118.0 (3)
C4A—C5A—H5A2	109.5	C18A—C17A—C16A	123.0 (3)
H5A1—C5A—H5A2	109.5	C22A—C17A—C16A	119.0 (3)
C4A—C5A—H5A3	109.5	C22B—C17B—C18B	117.7 (3)
H5A1—C5A—H5A3	109.5	C22B—C17B—C16B	123.5 (3)
H5A2—C5A—H5A3	109.5	C18B—C17B—C16B	118.9 (3)
C4B—C5B—H5B1	109.5	C19A—C18A—C17A	120.6 (4)
C4B—C5B—H5B2	109.5	C19A—C18A—H18A	119.7
H5B1—C5B—H5B2	109.5	C17A—C18A—H18A	119.7
C4B—C5B—H5B3	109.5	C19B—C18B—C17B	120.3 (4)
H5B1—C5B—H5B3	109.5	C19B—C18B—H18B	119.8
H5B2—C5B—H5B3	109.5	C17B—C18B—H18B	119.8
N1A—C6A—C8A	111.2 (2)	C20A—C19A—C18A	120.5 (4)
N1A—C6A—C7A	112.5 (2)	C20A—C19A—H19A	119.7
C8A—C6A—C7A	114.9 (2)	C18A—C19A—H19A	119.7
N1A—C6A—S1A	101.33 (16)	C20B—C19B—C18B	120.7 (4)
C8A—C6A—S1A	108.81 (17)	C20B—C19B—H19B	119.6
C7A—C6A—S1A	107.09 (18)	C18B—C19B—H19B	119.6
N1B—C6B—C8B	110.5 (2)	C21A—C20A—C19A	119.6 (4)
N1B—C6B—C7B	111.1 (2)	C21A—C20A—H20A	120.2
C8B—C6B—C7B	115.2 (2)	C19A—C20A—H20A	120.2
N1B—C6B—S1B	102.28 (15)	C19B—C20B—C21B	119.9 (4)
C8B—C6B—S1B	109.63 (16)	C19B—C20B—H20B	120.1
C7B—C6B—S1B	107.21 (19)	C21B—C20B—H20B	120.1
C6A—C7A—H7A1	109.5	C20A—C21A—C22A	120.4 (3)
C6A—C7A—H7A2	109.5	C20A—C21A—H21A	119.8
H7A1—C7A—H7A2	109.5	C22A—C21A—H21A	119.8
C6A—C7A—H7A3	109.5	C20B—C21B—C22B	120.1 (4)
H7A1—C7A—H7A3	109.5	C20B—C21B—H21B	119.9
H7A2—C7A—H7A3	109.5	C22B—C21B—H21B	119.9
C6B—C7B—H7B1	109.5	C21A—C22A—C17A	120.9 (3)
C6B—C7B—H7B2	109.5	C21A—C22A—H22A	119.6
H7B1—C7B—H7B2	109.5	C17A—C22A—H22A	119.6
C6B—C7B—H7B3	109.5	C21B—C22B—C17B	121.2 (3)
H7B1—C7B—H7B3	109.5	C21B—C22B—H22B	119.4
H7B2—C7B—H7B3	109.5	C17B—C22B—H22B	119.4
C9A—C8A—C13A	117.6 (3)	Cl2—C24—Cl3	112.3 (4)
C9A—C8A—C6A	121.6 (2)	Cl2—C24—Cl1	111.7 (3)
C13A—C8A—C6A	120.7 (2)	Cl3—C24—Cl1	108.8 (4)
C13B—C8B—C9B	117.3 (2)	Cl2—C24—H24	107.9
C13B—C8B—C6B	119.6 (2)	Cl3—C24—H24	107.9
C9B—C8B—C6B	123.0 (2)	Cl1—C24—H24	107.9
C10A—C9A—C8A	121.1 (3)	C2A—N1A—N3A	118.9 (2)
C10A—C9A—H9A	119.4	C2A—N1A—C6A	123.1 (2)
C8A—C9A—H9A	119.4	N3A—N1A—C6A	117.22 (19)
C10B—C9B—C8B	121.3 (3)	C2B—N1B—N3B	119.7 (2)
C10B—C9B—H9B	119.4	C2B—N1B—C6B	122.5 (2)
C8B—C9B—H9B	119.4	N3B—N1B—C6B	117.59 (19)
C11A—C10A—C9A	119.7 (3)	C4A—N2A—C3A	124.2 (2)
C11A—C10A—H10A	120.1	C4A—N2A—H2A	117.9
C9A—C10A—H10A	120.1	C3A—N2A—H2A	117.9
C11B—C10B—C9B	119.9 (3)	C4B—N2B—C3B	124.3 (2)
C11B—C10B—H10B	120.0	C4B—N2B—H2B	117.8
C9B—C10B—H10B	120.0	C3B—N2B—H2B	117.8
C10A—C11A—C12A	120.6 (3)	C3A—N3A—N1A	110.1 (2)
C10A—C11A—O3A	116.6 (3)	C3B—N3B—N1B	110.6 (2)
C12A—C11A—O3A	122.7 (3)	C14A—O3A—C11A	119.8 (2)
C12B—C11B—C10B	120.3 (3)	C14B—O3B—C11B	117.8 (2)
C12B—C11B—O3B	120.9 (3)	C3A—S1A—C6A	89.27 (11)
C10B—C11B—O3B	118.6 (3)	C3B—S1B—C6B	90.27 (11)
			
N1A—C6A—C8A—C9A	18.9 (3)	C18B—C17B—C22B—C21B	−1.6 (5)
C7A—C6A—C8A—C9A	148.1 (3)	C16B—C17B—C22B—C21B	179.2 (3)
S1A—C6A—C8A—C9A	−91.9 (3)	O1A—C2A—N1A—N3A	174.7 (2)
N1A—C6A—C8A—C13A	−164.1 (2)	C1A—C2A—N1A—N3A	−4.5 (4)
C7A—C6A—C8A—C13A	−34.9 (3)	O1A—C2A—N1A—C6A	5.1 (4)
S1A—C6A—C8A—C13A	85.1 (3)	C1A—C2A—N1A—C6A	−174.1 (3)
N1B—C6B—C8B—C13B	63.4 (3)	C8A—C6A—N1A—C2A	73.6 (3)
C7B—C6B—C8B—C13B	−169.6 (3)	C7A—C6A—N1A—C2A	−56.8 (3)
S1B—C6B—C8B—C13B	−48.6 (3)	S1A—C6A—N1A—C2A	−170.9 (2)
N1B—C6B—C8B—C9B	−115.5 (3)	C8A—C6A—N1A—N3A	−96.1 (2)
C7B—C6B—C8B—C9B	11.5 (4)	C7A—C6A—N1A—N3A	133.4 (2)
S1B—C6B—C8B—C9B	132.5 (3)	S1A—C6A—N1A—N3A	19.4 (2)
C13A—C8A—C9A—C10A	−0.4 (4)	O1B—C2B—N1B—N3B	178.5 (2)
C6A—C8A—C9A—C10A	176.7 (3)	C1B—C2B—N1B—N3B	−2.1 (4)
C13B—C8B—C9B—C10B	0.6 (5)	O1B—C2B—N1B—C6B	3.1 (4)
C6B—C8B—C9B—C10B	179.5 (3)	C1B—C2B—N1B—C6B	−177.6 (2)
C8A—C9A—C10A—C11A	−0.1 (5)	C8B—C6B—N1B—C2B	65.2 (3)
C8B—C9B—C10B—C11B	−1.2 (5)	C7B—C6B—N1B—C2B	−64.0 (3)
C9A—C10A—C11A—C12A	0.4 (5)	S1B—C6B—N1B—C2B	−178.2 (2)
C9A—C10A—C11A—O3A	−175.4 (3)	C8B—C6B—N1B—N3B	−110.4 (2)
C9B—C10B—C11B—C12B	0.9 (5)	C7B—C6B—N1B—N3B	120.4 (2)
C9B—C10B—C11B—O3B	−174.3 (3)	S1B—C6B—N1B—N3B	6.3 (2)
C10A—C11A—C12A—C13A	−0.1 (5)	O2A—C4A—N2A—C3A	−5.8 (4)
O3A—C11A—C12A—C13A	175.4 (3)	C5A—C4A—N2A—C3A	172.6 (3)
C10B—C11B—C12B—C13B	0.1 (6)	N3A—C3A—N2A—C4A	174.3 (2)
O3B—C11B—C12B—C13B	175.2 (3)	S1A—C3A—N2A—C4A	−7.2 (4)
C11A—C12A—C13A—C8A	−0.4 (4)	O2B—C4B—N2B—C3B	−0.7 (5)
C9A—C8A—C13A—C12A	0.7 (4)	C5B—C4B—N2B—C3B	178.8 (3)
C6A—C8A—C13A—C12A	−176.5 (3)	N3B—C3B—N2B—C4B	176.4 (3)
C9B—C8B—C13B—C12B	0.4 (5)	S1B—C3B—N2B—C4B	−4.9 (4)
C6B—C8B—C13B—C12B	−178.5 (3)	N2A—C3A—N3A—N1A	178.5 (2)
C11B—C12B—C13B—C8B	−0.7 (6)	S1A—C3A—N3A—N1A	0.0 (3)
O4A—C14A—C15A—C16A	2.2 (5)	C2A—N1A—N3A—C3A	175.8 (2)
O3A—C14A—C15A—C16A	−176.0 (3)	C6A—N1A—N3A—C3A	−14.0 (3)
O4B—C14B—C15B—C16B	−14.4 (5)	N2B—C3B—N3B—N1B	178.6 (2)
O3B—C14B—C15B—C16B	165.3 (3)	S1B—C3B—N3B—N1B	−0.2 (3)
C14A—C15A—C16A—C17A	−180.0 (3)	C2B—N1B—N3B—C3B	179.9 (2)
C14B—C15B—C16B—C17B	−177.9 (3)	C6B—N1B—N3B—C3B	−4.4 (3)
C15A—C16A—C17A—C18A	−5.9 (5)	O4A—C14A—O3A—C11A	−2.0 (5)
C15A—C16A—C17A—C22A	171.7 (3)	C15A—C14A—O3A—C11A	176.3 (2)
C15B—C16B—C17B—C22B	−9.2 (5)	C10A—C11A—O3A—C14A	−125.4 (3)
C15B—C16B—C17B—C18B	171.7 (3)	C12A—C11A—O3A—C14A	58.9 (4)
C22A—C17A—C18A—C19A	1.0 (6)	O4B—C14B—O3B—C11B	5.0 (5)
C16A—C17A—C18A—C19A	178.6 (4)	C15B—C14B—O3B—C11B	−174.7 (3)
C22B—C17B—C18B—C19B	0.9 (6)	C12B—C11B—O3B—C14B	68.0 (4)
C16B—C17B—C18B—C19B	−179.8 (4)	C10B—C11B—O3B—C14B	−116.7 (3)
C17A—C18A—C19A—C20A	−0.9 (7)	N3A—C3A—S1A—C6A	9.8 (2)
C17B—C18B—C19B—C20B	0.6 (7)	N2A—C3A—S1A—C6A	−168.6 (2)
C18A—C19A—C20A—C21A	0.0 (7)	N1A—C6A—S1A—C3A	−14.77 (17)
C18B—C19B—C20B—C21B	−1.5 (8)	C8A—C6A—S1A—C3A	102.52 (18)
C19A—C20A—C21A—C22A	0.7 (6)	C7A—C6A—S1A—C3A	−132.8 (2)
C19B—C20B—C21B—C22B	0.9 (7)	N3B—C3B—S1B—C6B	3.4 (2)
C20A—C21A—C22A—C17A	−0.5 (5)	N2B—C3B—S1B—C6B	−175.3 (2)
C18A—C17A—C22A—C21A	−0.4 (5)	N1B—C6B—S1B—C3B	−4.89 (16)
C16A—C17A—C22A—C21A	−178.1 (3)	C8B—C6B—S1B—C3B	112.41 (18)
C20B—C21B—C22B—C17B	0.7 (7)	C7B—C6B—S1B—C3B	−121.86 (19)

### 4-(5-Acetamido-3-acetyl-2-methyl-2,3-dihydro-1,3,4-thiadiazol-2-yl)phenyl cinnamate chloroform hemisolvate (IV) Hydrogen-bond geometry (Å, º)

**Table:**

*D*—H···*A*	*D*—H	H···*A*	*D*···*A*	*D*—H···*A*
N2*A*—H2*A*···O1*B*^i^	0.86	1.96	2.815 (3)	172
N2*B*—H2*B*···O1*A*^ii^	0.86	1.96	2.810 (3)	169
C5*A*—H5*A*2···O1*B*^i^	0.96	2.56	3.344 (4)	139
C5*A*—H5*A*3···O4*A*^i^	0.96	2.54	3.477 (4)	164
C12*B*—H12*B*···O2*A*^iii^	0.93	2.56	3.459 (4)	161

Symmetry codes: (i) *x*, *y*−1, *z*; (ii) −*x*+1, −*y*+1, −*z*; (iii) *x*−1, *y*+1, *z*.
